# Toward Integrated Multi-Omics Intervention: Rice Trait Improvement and Stress Management

**DOI:** 10.3389/fpls.2021.741419

**Published:** 2021-10-15

**Authors:** Zahra Iqbal, Mohammed Shariq Iqbal, M. Iqbal R. Khan, Mohammad Israil Ansari

**Affiliations:** ^1^Molecular Crop Research Unit, Department of Biochemistry, Chulalongkorn University, Bangkok, Thailand; ^2^Amity Institute of Biotechnology, Amity University Uttar Pradesh, Lucknow, India; ^3^Department of Botany, Jamia Hamdard, New Delhi, India; ^4^Department of Botany, University of Lucknow, Lucknow, India

**Keywords:** genomics, metabolomics, omics, proteomics, rice, stress, transcriptomics

## Abstract

Rice (*Oryza sativa*) is an imperative staple crop for nearly half of the world’s population. Challenging environmental conditions encompassing abiotic and biotic stresses negatively impact the quality and yield of rice. To assure food supply for the unprecedented ever-growing world population, the improvement of rice as a crop is of utmost importance. In this era, “omics” techniques have been comprehensively utilized to decipher the regulatory mechanisms and cellular intricacies in rice. Advancements in omics technologies have provided a strong platform for the reliable exploration of genetic resources involved in rice trait development. Omics disciplines like genomics, transcriptomics, proteomics, and metabolomics have significantly contributed toward the achievement of desired improvements in rice under optimal and stressful environments. The present review recapitulates the basic and applied multi-omics technologies in providing new orchestration toward the improvement of rice desirable traits. The article also provides a catalog of current scenario of omics applications in comprehending this imperative crop in relation to yield enhancement and various environmental stresses. Further, the appropriate databases in the field of data science to analyze big data, and retrieve relevant information *vis-à-vis* rice trait improvement and stress management are described.

## Introduction

Rice (*Oryza sativa*) is a staple crop for billions of the world population. World agriculture faces a daunting task to proportionally ramp-up rice production for meeting the enormous demand of human consumption (Khan et al., 2015). Concomitantly, adverse environmental conditions negatively impact rice production and cause significant yield loss. Biotic and abiotic stresses either in combination or individually prevent the attainment of full genetic potential for optimal rice growth and yield (Raza et al., 2019). According to the 2021 data of FAOSTAT (Food and Agricultural organization), rice is one of the highest globally harvested crops^1^. According to the latest census by FAOSTAT in the year 2019, around 160 million hectares of land is planted with rice which cumulatively produces approximately 750 million tons of rice worldwide (see text footnote 1). Asia is the leading producer of rice and contributes to about 90.6% of the production share (see text footnote 1).

Apart from the immense economic importance, rice has also emerged as a model crop (genome size = 4.3MB) for monocots (Izawa and Shimamoto, 1996). The simple genome of rice led to easy and early genome sequencing of rice (Goff et al., 2002; Yu et al., 2002; Sasaki, 2005; Huang et al., 2013; Stein et al., 2018). For the two popular rice sub-species namely, *O. sativa* ssp. *Japonica* and *O. sativa* ssp. *Indica*, the pioneer draft genome was released in the year 2002 (Goff et al., 2002; Yu et al., 2002). Following the release of the draft genome, high-throughput technologies were employed to assemble the complete reference genome of rice (Sabot et al., 2011; Gao et al., 2013). Much recently, the genome availability of 13 domesticated and wild rice varieties has highlighted the genetic conservation across the genus *Oryza* (Stein et al., 2018).

In molecular biology and data science, the word “ome” refers to the study of special, temporal, and global changes occurring in an organism. Omics is a branch of science to gauge the functions and extract relevant biological information in a single or bunch of cells, tissues, or organs. The easy accessibility of whole-genome sequences from rice provides a platform for several omic studies like genomics, transcriptome, proteome, and metabolome (Delseny et al., 2001; Komatsu and Tanaka, 2005; Agrawal and Rakwal, 2011; Kyndt et al., 2012; Zheng et al., 2013; Chen et al., 2014; Lin et al., 2017; Li et al., 2018; Song S. et al., 2018; Zarei et al., 2018; Zhang et al., 2019; Peng Yuan et al., 2020). These techniques form the core components of omics technology. Over the years, substantial progress has been made in these methods in relation to almost all organelles, cells, tissues, and organs of rice. Rice genomics led to the discovery and functional characterization of pivotal genes that play crucial roles in improving rice productivity (Yano et al., 2016; Tang et al., 2019; Volante et al., 2020). The application of transcriptomics to rice has widened the understanding of complex molecular responsive mechanisms, differential gene expression, and regulatory pathways under varying conditions (Takehisa et al., 2012; Kumar and Dash, 2019; Sun et al., 2019). This information can be successfully processed for rice crop improvement. Similarly, proteomics and metabolomics has also contributed drastically for rice trait improvements (Oikawa et al., 2008; Agrawal and Rakwal, 2011; Calingacion et al., 2012; Kim S.T. et al., 2014; Baslam and Mitsui, 2020). The application of secretome for the identification of various novel secreted proteins and global mapping of phosphorylation sites is also worth mentioning (Cho and Kim, 2009; Agrawal et al., 2010; Que et al., 2012; Chen et al., 2016). Additionally, well-recognized proteomes have abetted in re-annotating the rice genome to unravel the proteins of unidentified functions. These relevant findings are implemented for genetic improvement in relation to agronomic traits and response to biotic/abiotic stresses. The major challenge ahead for functional genomics and system biology is to integrate genomics, transcriptomics, proteomics, and metabolomic information for a better understanding of cellular biology. The present review recapitulates the core omics techniques *viz.*, genomics, transcriptomics, proteomics, and metabolomics to emphasize the advances achieved in rice omics research. Further, this review is aimed to reiterate the existing rice-omics scenario and how the implication of data science is gaining significance for rice trait improvement and stress management across the scientific community.

## Genomics and Transcriptomics: An Overview

**FIGURE 1:**
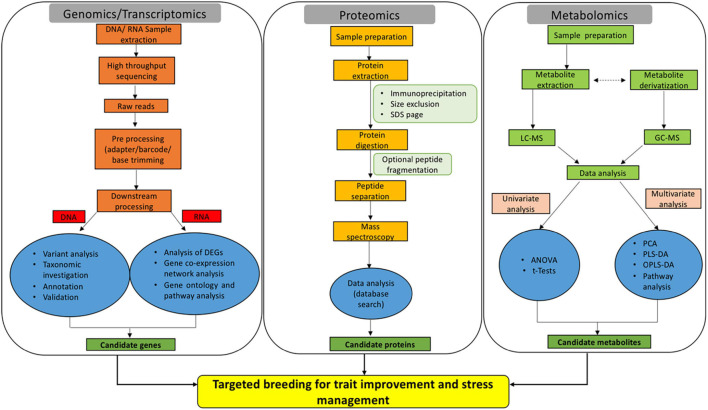
Omics-based approaches are emerging as efficient tools for dissecting the key genes, proteins, and metabolites implicated in rice trait improvement and stress acclimation responses.

Genomics is defined as the study of structure, function, evolution, and interaction of genes which provides complete information about the genetic make-up of an organism. The core components of genomics include genetic engineering, DNA sequencing, and deep analysis of the functions of genome. Genetic code is considered the foundation of biological life. The prime resources for understanding the genome involve the sequencing of DNA code and studying the gene expression patterns. The complete genome sequencing of *Arabidopsis thaliana* (Kaul et al., 2000) ushered to the post-genomic era in plant research. In the year 2005, the rice genome was sequenced under International Rice Genome Sequencing Project (Sasaki, 2005). The neoteric advances in the DNA marker technologies for identifying Single Nucleotide Polymorphism (SNP) have resulted in uncovering desirable traits. Massive parallel sequencing commonly referred to as next generation sequencing (NGS) has revolutionized the research underlying plant sciences (Figure 1). NGS utilizing Illumina/Solexa, Ion Torrent Personal Genome Machine (PGM) and Pacific Biosciences (PacBio) techniques have completely transformed the genomic and transcriptomic studies through their accuracy and robustness (Dhondt et al., 2013; Heather and Chain, 2016). Genome-wide association studies (GWAS) and quantitative trait loci (QTL) mapping to comprehend the genetic variance and inheritance of complex quantitative traits have also gained considerable significance in the recent past (Bekele et al., 2013; Bao, 2014; Reig-Valiente et al., 2018). Thus, genomics delivers fast and accurate approaches for crop biotechnology by enabling methods for marker-assisted selection and molecular breeding.

Next, transcriptomics deals with the study of the entire transcriptome (sum of all RNA transcript) of an organism at a particular developmental stage or under a specific physiological condition (Blumenberg, 2019). Studying the transcriptomes of a variety of diverse populations aid in linking the genotype to a particular phenotype. Enormous population-wide transcriptome studies have been conducted on rice and other agronomic crops to understand the underlying mesh of networks in crop improvement (Kremling et al., 2018; Groen et al., 2020; Iqbal et al., 2020b). Nonetheless, due to the limitations associated with the sampling of below-ground tissues, the majority of transcriptomic studies are focused on above-ground tissues (Yoshino et al., 2019). In crux, transcriptomic studies allow the identification of mRNA, long non-coding RNAs, and small RNAs as well as the understanding of gene organizations and expression profiles (Figure 1; Wang et al., 2009). Generally, transcriptomic methods rely on sequencing [serial analysis of gene expression- SAGE (Moustafa and Cross, 2016), expressed sequence tags- ESTs (Parkinson and Blaxter, 2009) and RNA-seq (Wang et al., 2009)] or hybridization [suppression subtractive hybridization-SSH (Sahebi et al., 2015) and microarray (Hrdlickova et al., 2017)]. RNA-seq is considered as the best approach in comparison to other hybridization or sequencing-based methods, as far as the coverage and resolution are considered. ESTs (Zhou et al., 2003), SAGE (Bao et al., 2005), SSH (Chang et al., 2019), microarray (Wang et al., 2016), and RNA-seq (Ereful et al., 2020; Zainal-Abidin et al., 2020; Divya et al., 2021) have been linked extensively in the elucidation of complex mechanisms for rice trait improvement and stress management.

### Genomics and Transcriptomics in Rice Trait Improvement

The major challenge ahead of rice breeders is to enhance rice productivity and improve related agronomic traits. This task becomes difficult to accomplish using traditional breeding techniques. The difficulty is further aggravated due to epistatic interactions of yield contributing genes (Mei et al., 2006). Taking into account the quality of rice, genomics and transcriptomics have offered several breakthroughs. Generally, the characteristics linked with rice quality include taste, gel consistency, amylose content, texture, aroma, nutritional value, gelatinization temperature, and resistant storage. Genetically modified methods have been frequently utilized to improve the above-mentioned characteristics. One of the early examples includes the generation of golden rice that contains significant levels of beta-carotene (Ye et al., 2000). Golden rice was produced by transforming the exogenous genes *psy*, *crtl*, and *lcy*, along with their upstream elements. Beta-carotene is a known precursor in vitamin A synthesis and hence golden rice is considered nutritionally rich in comparison to white rice. Nonetheless, efficient technologies such as DNA markers for marker-assisted selection (MAS) of agronomic traits are pivotal to yield and quality improvement (Das et al., 2017). Minor and major QTLs for some yield components, *viz.*, plant height, spikelets per panicle, length of panicle, length of grain, weight of grain, yield per grain, and harvest index have been identified (Table 1; Septiningsih et al., 2003; Bernier et al., 2007). The QTLs contributing for grain length (*qGL3*), grain width and weight (*qGW2*), grain weight (*qgw3*), grain number (*qGn1*), grain length and weight (*qGS3*), and plant height (*Ph1*) are based on Mendelian factors (Li et al., 2004; Fan et al., 2006; Wan et al., 2006; Ashikari et al., 2007; Song et al., 2007; Qi et al., 2017). For example, the *GW2* gene (RING-type protein) is localized on chromosome 2 that modulates the width and weight of rice grains (Song et al., 2007). *GS3* (putative transmembrane protein) is localized on chromosome 3 that modulates grain width, weight, thickness, and length (Fan et al., 2006). Such important alleles and genes linked to DNA markers can be potentially utilized in MAS for improving rice yield and quality. The accessibility of sequenced rice genome accelerated the identification of polymorphic markers (Feltus et al., 2004; Shen et al., 2004). Centromeres of rice chromosomes have also been successfully sequenced and assembled. This information can be applied for the construction of artificial rice chromosomes (Nagaki et al., 2004; Wu et al., 2004). Such data can be directly linked to phenotypic traits and successfully used for functional analysis. In recent times, gene cloning and functional analysis linked to yield and quality in important rice cultivars have been significantly improved by various scrupulous approaches. These encompass mutant screening, comparative genome analysis, production of cross populations, and identification of wild variates with better qualitative and quantitative traits (Shomura et al., 2008; Jiang et al., 2012).

**TABLE 1:** List of QTLs in rice trait improvement and stress management.

QTL	Trait/stress	References
*qYLD3-1, qPW3-1, qPH12-1, qFLW4-1, and qPL3-1*	total grain yield, panicle weight, plant height, flag leaf width, panicle length	Kulkarni et al., 2020
*qtl12.1/qDTY12.1*	decreased number of days to flowering, higher harvest index, increased biomass and plant height	Bernier et al., 2007
*qDTY1.1*	Grain yield under drought and plant height under drought	Ghimire et al., 2012 Venuprasad et al., 2012b Vikram et al., 2016
*qDTY2.1, qDTY3.1, qDTY2.2, qDTY9.1*, and *qDTY12.1*	grain yield under drought stress	Bernier et al., 2007; Venuprasad et al., 2009; Swamy et al., 2011; Mishra et al., 2013
*qDTY1.1, qDTY1.3*, and *qDTY8.1*	grain yield under drought stress	Catolos et al., 2017
*qRT9.1* and *qRT5.1*	Root trait under drought stress	Catolos et al., 2017
*qDTY1*.*1*, *qDTY3*.*3*, and *qDTY6*.*3*	grain yield across the seasons under severe and moderate drought	Yadav et al., 2019
*Saltol*	Salt stress	Gregorio, 1997; Bonilla et al., 2002; Niones, 2004; Thomson et al., 2010; Alam et al., 2011
*qSKC-1* and *qSNC-1*	Shoot potassium concentration and shoot sodium concentration	Zhou et al., 2013; Deng et al., 2015b; Jing et al., 2017
*qSL2*, *qRL2.1, qSIS2 qSDW2*, and *qRL2.2*	shoot length, root length, salt injury score, and shoot dry weight	Amoah et al., 2020
*qCT3.12, qCT6.7, and qCT9.6*	QTL affecting cold tolerance associated with spikelet fertility (%)	Liang et al., 2018
*qLTGR4d-9-1, qLTGR4d-9-2, qLTGR2d-9-1, qLTGI-9-1, qLTGR2d-9-2, and qLTGI-9-2*	QTLs associated with seed germination under cold stress in the RIL population of rice	Yang et al., 2020
*qLTSR-9-2, qLTSR-9-1, qLTSR-9-1, qLTNSR-9, and qLTNSR-9*	QTLs associated with cold tolerance of the RIL population of rice at the bud stage	Yang et al., 2020
*MQTL2.5, MQTL8.1, and MQTL9.1*	significant count of *R*-genes contributing to sheath blight, rice blast, and bacterial blight resistance	Kumar and Nadarajah, 2020
*qBL3*	Leaf and neck blast resistance	Devi et al., 2020
*SUB1*	Submergence stress	Xu et al., 2006; Fukao and Bailey-Serres, 2008; Chakraborty et al., 2021
*AG1* and *AG2*	anaerobic germination under flooding	Mondal et al., 2020

One of the early examples from QTL mapping involves dense and erect panicle 1 (DEP1) accountable for governing the number, weight, and size of rice grain (Ashikari et al., 2005; Huang, Feng et al., 2009). Furthermore, QTLs are also linked to ideal plant architecture (IPA) and wealthy farmer’s panicle (WFP) (Jiao et al., 2010; Miura et al., 2010). IPA and WFP contribute to a greater number of panicles branching and higher grain yield in rice. Later, recombinant inbred lines (RILs) population was used to detect 27 QTLs on 10 rice chromosomes (Yan et al., 2014). The RILs in this study were obtained from a cross of Huahui 3 (*Bt/Xa21*) and Zhongguoxiangdao. 12 of these QTLs contributed to rice grain shape and yield. Intriguingly, the two already known genes, *Bt* gene (insect-resistant) and *Xa21* gene (disease-resistant) were closely linked to QTLs responsible for grain shape and weight. In the Huahui 3 rice cultivar, *Bt* fragment insertion localized on chromosome 10. The *Bt* fragment insertion might disrupt grain-related QTLs which resulted in compromised yields in transgenics (Yan et al., 2014). The introgression of *Xa21* gene into Minghui 63 rice cultivar contained a donor linkage drag and affected QTL alleles to regulate the shape and yield of grain. This information can be utilized for breeding applications to recuperate rice grain shape and yield (Yan et al., 2014). Recently, another RIL population obtained from KRH-2 (IR58025A/KMR3R) was utilized to identify QTLs governing crop yield (Kulkarni et al., 2020). A genetic map of 294.2 cM with 126 simple sequence repeats (SSR) was made. Overall, 22 QTLs were recognized with phenotyping and genotyping data. The study reported a novel QTL linked to panicle length (*qPL3-1*). The other QTLs identified were total grain yield/plant (*qYLD3-1*), panicle weight (*qPW3-1)*, plant height (*qPH12-1)*, and flag leaf width (*qFLW4-1)*. Moreso, considerable epistatic interactions were detected for the length of panicle and grain yield per plant. *In silico* analysis of the QTLs highlighted the functions of candidate genes linked with preferred traits (Kulkarni et al., 2020). The high-yielding RILs harboring the yield associated QTLs were recognized as restorers. This indicates their probable deployment in the generation of excellent rice hybrids. Further, single-nucleotide polymorphism (SNP) data provides a strong foundation for exploring rice diversity and gene–trait relationships that can be successfully implemented in crop improvement through linkage mapping (McNally et al., 2009; McCouch et al., 2010; Roy and Lachagari, 2017; Zainal-Abidin et al., 2019). SNP markers linked with rice grain yield have been well documented recently. GWAS was used for genotyping of 541 rice accessions by 167470 SNPs. The study concluded 15 SNPs to be significantly related to grain yield (Pantalião et al., 2020). Additionally, trait-linked simple sequence repeat (SSR) markers were deployed to study an important rice agronomic trait-aroma (Jasim Aljumaili et al., 2018). The study quantified the genetic divergence using SSR markers in aromatic rice accessions. This led to the identification of promising accessions for introgression (Jasim Aljumaili et al., 2018). SSRs were also utilized to study colored rice germplasm (Black-Purple and Red Pericarp Color) (Park et al., 2019). Taking into account the nutritional quality, the genetic diversity of rice grain iron and zinc levels in the representative groups of local and exotic rice accessions was evaluated by SSR markers. Aromatic rice fine grain accessions contained high iron and zinc levels in brown rice in comparison to coarse grain accessions (Raza et al., 2020). Neoterically, GWAS and functional analysis of 520 rice accessions identified *OsZIP18* as the prime genetic determinant for regulating branched-chain amino acid levels (Sun et al., 2020). Thus, *OsZIP18* can be considered a potential gene for enhancing rice nutritional value. *OsZIP18* can be of significant importance as humans are unable to synthesize branched-chain amino acids.

Plant breeders have suggested the IPA that comprises many important agronomic traits such as low tiller counts, more grains per panicle, few or no unproductive tillers, and thick and strong stems (Jiao et al., 2010; Li et al., 2012). Back in 2010, semi-dominant QTL, *IPA1* (ideal plant architecture 1) was cloned and characterized. This QTL encodes a squamosa-promoter binding protein-like transcription factor (TF) named *OsSPL14*. *OsSPL14* can regulate few relevant genes such as *OsTB1* (negative regulator of lateral branching) (Takeda et al., 2003) and *DEP1* (grain yield-related protein) (Huang, Qian et al., 2009). Moreover, *OsSPL14* at the reproductive stage can facilitate higher grain yield and panicle branching at the reproductive stage (Jiao et al., 2010; Miura et al., 2010). Nonetheless, microRNA (*miR156*) negatively regulates *OsSPL14* (Xie et al., 2006). The *OsSPL14* mRNA is cleaved by *miR156* to suppress it functions. Transgenic rice with IPA characteristics was generated by incorporating point mutation at the *OsmiR156*-targeted site in *OsSPL14* (Jiao et al., 2010; Miura et al., 2010). Over-expressing *miR156* also leads to fast leaf/tiller initiation and advanced leaf maturation in rice (Xie et al., 2012). *GW8* gene positively regulates the yield and width of rice grains (Wang et al., 2012). A mutation in the promoter of *GW8* gene was found in the *indica* Basmati rice varieties. This mutation lowered the *GW8* expression and resulted in slender grain with a better appearance. Interestingly, *GW8* encodes OsSPL16 protein which is a target of *miR156*. In this case also, MAS was implemented to concomitantly upgrade the appearance and enhance the grain yield (Wang et al., 2012). Additionally, *OsmiR156* was reported to regulate the tillering-associated genes (*TB1, LAX1, and DWARF 53*) (Liu Q. et al., 2019). These studies demonstrated that *miR156* is a crucial regulator in rice. Another miRNA, *miR172* is associated with reduced seed weight, floral defects, and delayed transition of spikelet meristem to floral meristem in rice (Zhu et al., 2009). Furthermore, a miRNA/MADS/TCP/D14 (miMTD) regulatory system has also been reported to regulate tillering in rice (Guo et al., 2013). The expression of *OsMADS57* is negatively regulated by *OsMIR444a.* This in-turn negatively modulates the expression of *D14* to affect rice tillering. This mechanistic outline can be focused for high grain yield in rice breeding programs (Guo et al., 2013). *OsmiR397* is an endogenous rice miRNA that is expressed in seeds (Xue et al., 2009; Chen et al., 2011), undifferentiated, and differentiated calli (Luo et al., 2006). Over-expression of miRNA gene-*OsmiR397* is linked with grain size and panicle branching, eventually leading to increased rice grain production (Zhang et al., 2013). The *miR397* targets *OsLAC* gene (linked to brassinosteroid sensitivity) to cleave its mRNA and disrupt the overall function (Zhang et al., 2013). On similar lines, *miR159* targets *OsGAMYB and OsGAMYBL1* (*GAMYB-LIKE 1*) genes. The activity of mature *miR159* was hindered by STTM (Short Tandem Target Mimic). This resulted in enhanced expression of *OsGAMYB and OsGAMYBL1* with reduced size of organ, diameter of stem, length of flag leaf, size of grain panicle, and spikelet hulls (Zhao et al., 2017). On similar grounds, miRNA microarray profiling identified *miR319* expression has a suppressive effect on rice plant height (Liu et al., 2017). In a recent study, transgenic rice with disrupted *miR396*-targeting site in *OsGRF8*; and knockout of *miR396e* and *miR396f* exhibited improved panicle branching and grain size (Zhang J. et al., 2020). Likewise, down-regulation of *OsmiR1432* resulted in enhanced expression of *Acyl-CoA thioesterase (OsACOT)* to promote grain filling. The disruption of *OsmiR1432* lead to heavier grains with improved yield by atleast 17% (Zhao et al., 2019).

Early efforts to decipher the entire transcriptome began in the 1990s (Lowe et al., 2017). During the last decade, RNA-seq method that uses deep-sequencing technologies has gained huge popularity for various crop improvement programs. Generally, in RNA-seq, total or fractionated RNA is made into a library of cDNAs fragments. Either one or both the cDNA ends are ligated with adapters. Each molecule is then sequenced in a high-throughput manner to obtain a short stretch of sequences either from one end (single-end sequencing) or both ends (pair-end sequencing) (Figure 1). The read length may vary from 30 to 400 bp depending upon the sequencing technology. The sequenced reads are finally aligned either to a reference genome or assembled *de novo* to generate meaningful information (expression profile and transcriptional structure). As already discussed, transcriptomics aids in deciphering un-annotated genes and analyzing gene expression patterns (Lowe et al., 2017). RNA-seq was performed to gain insights into the genome-wide transcription patterns of *O. sativa japonica* and *indica* subspecies (Lu et al., 2010). Whilst most of the RNA-seq studies in rice are largely focused on stress management, few have also been conducted for improving agronomic traits. In this context, the RNA-seq approach was utilized to establish the involvement of alternative splicing in rice mineral nutrient homeostasis (Dong et al., 2018). This was further extended to large-scale GWAS and transcriptome studies to identify genes affecting the rice glycemic index (Anacleto et al., 2019). The glycemic index in rice is an important parameter for a large population of society suffering from Type II diabetes, obesity, and hypertension (Mohan et al., 2014). In a recent study, the rice genome annotation was improvised by RNA-seq experiments. The study resulted in the identification of 1584 new peptides and 101 new loci matched to novel peptides (Ren et al., 2019). The identification of these novel peptides and loci in the near future can be linked to traits of agronomic importance.

### Genomics and Transcriptomics in Rice Stress Management

The prime objective of rice research is to improve crop yield and acclimatization to unfavorable environmental conditions. The rice genome had been sequenced years back, but high-quality genome annotation of rice is necessary for the researchers working in this arena. In this direction, the accomplishment of Rice Annotation Project (RAP) database^2^ established on the new chromosome pseudomolecule Os-Nipponbare-Reference-IRGSP-1.0 (a joint version of IRGSP and MSU pseudomolecules) (Kawahara et al., 2013) was imperative. The preliminary response of plants toward stress is the induction of signal transduction pathways. Generally, the second messenger molecules in signal transduction cascades are responsible for the regulation of stress-responsive genes. The induction or suppression of stress-responsive genes in-turn generates an appropriate response.

### Abiotic Stress

The generation of transgenic plants for functional validation of genes associated with a particular trait heavily relies on genomics and transcriptomics. For instance, it had been shown that 5000 genes were upregulated and 6000 genes were downregulated upon drought exposure to rice (Bin Rahman and Zhang, 2016; Joshi et al., 2016). These genes are grouped into three main categories: membrane transport genes, signaling-related genes, and transcriptional regulatory genes (Upadhyaya and Panda, 2019; Kim et al., 2020). The expression of these genes in rice governs the biochemical, physiological, and molecular mechanisms under drought stress (Dash et al., 2018; Gupta et al., 2020). Further, considering the transgenic approach, numerous genes in rice are identified to be differentially expressed upon drought exposure (Kumar et al., 2017; Upadhyaya and Panda, 2019). The mode of regulation may be either ABA-dependent or ABA-independent (Du et al., 2018; Gupta et al., 2020). In this regard, *OsJAZ1* in an ABA-dependent manner attenuates drought tolerance in rice (Fu et al., 2017). Similarly, LEA proteins and osmoregulatory genes confer drought tolerance to rice plants (Dash et al., 2018; Upadhyaya and Panda, 2019). *OsPYL/RCAR5*, *EcNAC67* (Kim H. et al., 2014; Rahman et al., 2016), *OsDREB2B*, *CYP735A*, and *OsDREB1F* (Kim et al., 2020) are also involved in morphological adjustments of rice upon drought exposures. Additionally, the DREB2-like gene *OsDRAP1* has been reported in modulating drought tolerance (Huang et al., 2018). Recently, an allele of the flowering gene *OsMADS18* was shown to be a potential candidate in drought tolerance during breeding (Groen et al., 2020). An increase in rice grain yield upon drought exposure is also accomplished by transgenic approaches. This includes generation of transgenics with genes namely, *OsLEA3-1* (Xiao et al., 2007), *OsbZIP71* (Liu C. et al., 2014), *OsWRKY47* (Raineri et al., 2015), *OsbZIP46* (Tang et al., 2012), and *OsNAC10* (Jeong et al., 2010). In a similar vein, in response to salinity stress, *OsCOIN*, *OsDREB2A*, *OsMYB2*, *OsbZIP71*, *OsbZIP23* are reported as key players in the accretion of osmoprotectants and antioxidants, enhanced transporter activity for sodium and potassium ions (Liu et al., 2007; Xu et al., 2008; Sun et al., 2010; Takasaki et al., 2010; Yang et al., 2012; Gumi et al., 2018), regulation of other salt-responsive genes (Nakashima et al., 2007; Wang et al., 2008; Jan et al., 2013; Liu C. et al., 2014), improved fresh weight (Huang et al., 2007), stomatal closure (Hu et al., 2006), and high seedling survival (Hu et al., 2008; Mallikarjuna et al., 2011). The gain of function of these salt-responsive genes permits the transgenic rice plants to have adequate osmoregulation and less oxidative damage. A recent study advocates that *OsSTAP1* is an AP2/ERF transcriptional activator that positively controls salt tolerance. *OsSTAP1* works by reducing the sodium/potassium ratio and sustaining cellular redox homeostasis (Wang et al., 2020). Taking cold stress into consideration, *OsbHLH1* (Wang et al., 2003), *OsDREB1G* (Moon et al., 2019), *OsCTZFP8* (Jin et al., 2018), *OsICE1* and *OsICE2* (Deng et al., 2017) are few of the many rice genes implicated in cold acclimatization and tolerance. Furthermore in rice, methylation profiles and transcriptional responses to cold at the seedling stage have also been reported in the recent past (Guo et al., 2019).

As discussed in the previous section DNA markers and MAS are indispensable components of plant breeding (Das et al., 2017). Grain yield upon stress exposure is the chief trait associated with breeding programs (Bernier et al., 2007; Venuprasad et al., 2007; Kumar et al., 2008). Identifying QTLs linked with stress tolerance or susceptibility can assist breeders to choose desired genotypes with less yield compensation (Table 1; Shanmugavadivel et al., 2017). Grain yield itself is a complex trait and in combination with stress, becomes enormously challenging. Thus, the selection and determination of traits for QTL mapping under unfavorable environmental cues is crucial. A major QTL for grain yield upon drought exposure was identified in 2007 (Bernier et al., 2007). Under drought conditions, a sum total of 436 F3 derived lines from Vandana and Way Rarem were QTL mapped. A major QTL (*qtl12.1/qDTY12.1*) was identified between SSR markers, namely RM28048 and RM511. This QTL was linked with decreased number of days to flowering, higher harvest index, increased biomass, and plant height (Bernier et al., 2007). Later, in 2009 the influence of *qtl12.1* was evaluated under varied target population of environments (Bernier et al., 2009). The results were consistent with the same effect on grain yield upon drought across various environments. Nonetheless, the uniformity of major yield QTL under adverse conditions in different genetic backgrounds is equally important. Eventually, 3 rice populations (N22/IR64, N22/MTU1010, and N22/Swarna) were evaluated and mapped for a major grain yield QTL, *qDTY1.1* (Vikram et al., 2011). Across all the 3 populations, *qDTY1.1* was mapped on chromosome 1 and was considered appropriate for marker-assisted breeding. Moreso, bulk segregant analysis identified *qDTY1.1* in the genetic background of Swarna and IR64 rice cultivars (Ghimire et al., 2012). Upon drought exposure, *qDTY1.1* accounted for 32 and 9.3% of the phenotypic variation in Swarna and IR64 respectively for grain yield (Ghimire et al., 2012). Additionally, *qDTY1.1* was found to be associated with plant height (*sd1*) in Vandana/IR64 populations (Venuprasad et al., 2012b). Consequently, in large segregating populations recombinant alleles with un-associated *sd1* and *qDTY1.1* might generate drought-tolerant varieties with shorter height (Vikram et al., 2016). Similarly, *qDTY2.1, qDTY3.1, qDTY2.2, qDTY9.1*, and *qDTY12.1* are also reported for grain yield under drought stress (Bernier et al., 2007; Venuprasad et al., 2009; Swamy et al., 2011; Mishra et al., 2013). Another QTL, *qDTY6.1* mapped on chromosome 6 in the genetic backgrounds of Apo/Swarna, Apo/IR72, and Vandana/IR72. *qDTY6.1* explains the genetic variance (40–66%) for grain yield under aerobic conditions and enhanced the performance of Swarna and IR72 (drought-susceptible cultivars) under aerobic conditions (Venuprasad et al., 2012a). Catolos et al. (2017), further identified 3 major QTLs contributing to grain yield, namely *qDTY1.1, qDTY1.3*, and *qDTY8.1* as well as 2 major QTLs for root trait, namely *qRT9.1* and *qRT5.1*. The mapping population was produced by crossing Dular (drought-tolerant) and IR 64_21 (drought-sensitive). Neoterically, high-density linkage map of rice was constructed by genotyping-by-sequencing (Yadav et al., 2019). The linkage map was generated by employing two BC_1_F_3_ mapping populations namely Swarna^∗^2/Dular and IR11N121^∗^2/Aus196. The study identified six *qDTY* QTLs (three consistent effect QTLs) in Swarna^∗^2/Dular and eight *qDTY* QTLs (two consistent effect QTLs) in IR11N121^∗^2/Aus 196 mapping population. The relative analysis further identified four stable new QTLs, namely *qDTY2*.*4*, *qDTY3*.*3*, *qDTY6*.*3*, and *qDTY11*.*2* accounting for 8.62 to 14.92% phenotypic variance. Three QTLs (*qDTY1*.*1*, *qDTY3*.*3*, and *qDTY6*.*3*) were linked to grain yield across the seasons under severe and moderate drought (Yadav et al., 2019). Contrary to drought, submergence stress is a phenomenon associated with exposure of plants to excessive water for longer periods. An important QTL associated with submergence tolerance is *SUB1* (*submergence 1*) (Xu et al., 2006; Fukao and Bailey-Serres, 2008). A recent study affirmed that *SUB1* influences concomitant leaf gas film thickness and surface hydrophobicity (Chakraborty et al., 2021). Leaf gas film provides improved ethylene dissipation and decreased *in-planta* accumulation. This eventually results in the delay of ethylene-induced leaf senescence upon submergence stress (Chakraborty et al., 2021). Another flooding stress-related condition involves the exposure of plants to hypoxia. QTLs for hypoxia tolerance in rice were identified during the germination stage (Kim and Reinke, 2018). Genotypic data from Illumina 6K SNP chip was used to identify QTLs related to tolerance of anaerobic germination (AG). Rice lines with *qAG1b* + *qAG1a* + *qAG8* possessed 50%, *qAG1b* + *qAG1a lines possessed 36%, while qAG1b* + *qAG8* possessed 32% of survival rate under anaerobic conditions (Kim and Reinke, 2018). In yet another study, responses of *AG1* and *AG2* QTL ILs were assessed during anaerobic germination under flooding. The study revealed that genotypes with *AG1* and *AG2* had greater seedling emergence and faster elongation in flooded soils (Mondal et al., 2020).

Much alike drought, salinity tolerance is a genetically and physiologically complex trait that is governed by a distinctive set of QTLs (Moradi et al., 2003). It is well established that salinity tolerance is autonomous at the seedling stage and reproductive stage (Mohammadinezhad et al., 2010). The major salt-tolerant QTL identified is *Saltol* QTL which has been extensively deployed worldwide to generate better performing rice cultivars (Gregorio, 1997; Krishnamurthy et al., 2020; Yadav et al., 2020). *Saltol* QTL was identified in IR29 (sensitive variety) and Pokkali (tolerant variety) RIL population which mapped on chromosome 1. AFLP markers (P3/M9-8 and P1/M9-3) flanks the *Saltol* QTL resulting in 64.3–80.2% of the phenotypic variance. This QTL is associated with low sodium levels in plants. *Saltol* QTL has been further fine mapped between the SSR markers RM1287 and RM7075 (10.71 and 15.12 Mb) that comprise the SKC1 locus (Bonilla et al., 2002; Niones, 2004; Thomson et al., 2010; Alam et al., 2011). Several other QTLs had been identified for traits such as shoot sodium concentration (SNC), shoot potassium concentration (SKC), and shoot sodium/potassium ratio (Lin et al., 2004; Ren et al., 2005; Haq et al., 2010; Pandit et al., 2010; Zheng et al., 2015). Bimpong laboratory (Bimpong et al., 2014a, b) fine mapped QTLs for salinity stress tolerance deploying Hasawi as a salt-tolerant donor parent. They used SNPs for genotyping and linkage map preparation. Furthermore, the QTLs namely, *qSKC-1* and *qSNC-1* were mapped in F2 mapping populations derived from *rss2* and *rss4* (Nipponbare) as well as Zhaiyeqing8 (*indica*) (Zhou et al., 2013; Deng et al., 2015b). Later in 2017, *qSKC-1* was finely mapped between the markers RM578 and IM8854 within 45 kb region in F2 populations derived from Nipponbare/ZYQ8 and rss4/ZYQ8 (Jing et al., 2017). Similarly, *rst1* mutant (*rice salt-tolerant 1*) was used to reveal that *rst1* is regulated by a recessive gene (Deng et al., 2015a). QTL mapping was performed between *rst1* and Peiai 64 to identify the possible loci of the *rst1* gene, which was found on chromosome 6 (Deng et al., 2015a). Additionally, RILs obtained from IR29 (salt-sensitive) and Hasawi (salt-tolerant) were used by Bizimana et al. (2017) to identify the QTLs on chromosomes 1, 2, 4,6, 8, 9, and 12. None of the *Saltol* or QTLs were found near this position. This indicated that tolerance in the cultivar Hasawi is attributed to new QTLs which are different from *Saltol/SKC1* (Bizimana et al., 2017). Apart from QTLs/genomic regions linked to salt tolerance based on bi-parental mapping populations, an association panel following GWAS approaches to study marker-trait association has also been used (Emon et al., 2015; Kumar et al., 2015). 20 SNPs were identified to be significantly linked with sodium/potassium ratio (Kumar et al., 2015). Also, this study could identify the *Saltol* region, which accounts for salinity tolerance as a prime link with sodium/potassium ratio (Kumar et al., 2015). In an identical manner, Wn11463, an STS marker for *SKC1*, and RM22418 on chromosome 8 were identified at the seedling stage to be linked with salinity tolerance (Emon et al., 2015). Very recently, 308 F_4_ families from Sahel 317/Madina Koyo were evaluated using SNPs for salt tolerance at the early seedling stage (Amoah et al., 2020). The genotypic data were regressed on to their phenotype to detect the QTLs, and a high-density genetic map was prepared with 3698 SNPs. Multiple interval mapping revealed 13 QTLs associated with shoot length, root length, salt injury score, and shoot dry weight on chromosomes 2, 3, 4, 6, 7, 10, and 12. On chromosome 2, three QTLs (*qSL2*, *qRL2.1*, and *qSIS2*) and two QTLs (*qSDW2* and *qRL2.2*) were tightly linked, while on chromosome 7, another two QTLs (*qSDW7* and *qSL7*) were strongly associated (Amoah et al., 2020). Taking cold tolerance into account, RILs derived from Dasanbyeo (*indica*)/TR22183 (*japonica*) crosses in Yanji (high-latitude area), Kunming (high-altitude area), Chuncheon (cold water irrigation) and Suwon (normal) were used to study the influence of QTL and epistatic QTL (E-QTL) with respect to cold-related traits at the reproductive stage. In three different cold treatment locations, six QTLs for spikelet fertility were detected. Furthermore, 57 QTLs and 76 E-QTLs were identified for nine cold-associated traits; out of them 19 QTLs and E-QTLs had substantial interaction of QTLs with environments (QEIs). This study illustrated that epistatic effects and QEIs are imperative for QTLs linked with cold tolerance (Jiang et al., 2011). QTLs controlling cold tolerance were also studied at germination and early seedling stages with RILs derived from crosses between *japonica* and *indica* sub-species. Composite interval mapping revealed five QTLs at the germination stage with 5.7–9.3% phenotypic variance explained, while nine QTLs were found at the early seedling stage with 5.8–35.6% phenotypic variance explained. The study reported only one common QTL, probably indicative of growth-stage specificity of cold tolerance (Ranawake et al., 2014). Another study performed at the reproductive stage in rice involved 84 BC_2_ cold tolerance introgression lines (ILs) that were generated through backcrossing. These cold tolerance ILs along with 310 random ILs were deployed for studying genetic networks fundamental to cold tolerance in rice. The segregation distortion method revealed seventeen major QTLs for cold tolerance in five selective introgression populations (Liang et al., 2018). Recently, RILs obtained from *indica* rice H335 (low temperature-tolerant) and *indica* rice CHA-1 (low temperature-sensitive) were used to detect QTLs linked with low-temperature tolerance at bud and germination stages. A high-density genetic map revealed 11 QTLs; among which six QTLs accounted for 5.13–9.42% phenotypic variation explained at the germination stage, while five QTLs accounted for 4.17–6.42% phenotypic variation explained at the bud stage (Yang et al., 2020).

Next generation sequencing that can robustly ascertain approximately all the RNAs in cells has been extensively deployed for miRNA analysis, particularly in identifying new or rice-specific stress-responsive miRNAs. A number of rice miRNAs are expressed upon encountering biotic and abiotic stresses (Table 2). The majority of stress responsive miRNAs are conserved and possess an analogous effect among rice and other plant species. For example, rice *miR398* modulate the expression of *Os-CSD1* and *Os-CSD2* (similar to its targets in *Arabidopsis thaliana* -Cu or Zn superoxide dismutases) as well as responses to abiotic and biotic stresses (Li et al., 2011). A prominent report of drought-induced miRNA in rice involves *miR169g*. *miR169g* is notably up-regulated upon drought exposure (Zhao et al., 2007; Jian et al., 2010; Zhou et al., 2010). Apart from the established role of *miR169g* in drought tolerance, it is also reported to be induced by salt stress to cleave mRNA of the NF-YA TF (Zhao et al., 2009). Moreso, *miR169g* negatively regulates rice immune responses against the blast fungus (Li et al., 2017). Similarly, *miR393* is also induced by both, salinity and drought conditions (Gao et al., 2011; Xia et al., 2012; Lu et al., 2018). In addition, *miR319* is down-regulated upon cold stress (Lv et al., 2010), however, when over-expressed it could increase cold tolerance after chilling acclimation in rice (Yang et al., 2013; Wang et al., 2014). Furthermore, the reproductive tissues of rice treated with drought, salt, and cold stresses were used to prepare small RNA libraries. The RNA libraries were sequenced to gain insights into the involvement of miRNAs is stress responses (Barrera-Figueroa et al., 2012). A number of stress-modulated miRNAs were identified by matching the expression patterns under control and stress conditions. This paved the discovery of new miRNAs that might play important roles in stress responses associated with rice (Barrera-Figueroa et al., 2012). Thus, a single miRNA can regulate the signaling cross-talk between various pathways related to environmental stresses and can be linked with several traits, indicating a pleiotropic effect. Contrary to the pleiotropic effect, distinct miRNAs might contribute to a common function. For instance, *miR169*, *miR397*, *miR528*, *miR827*, *miR1425*, *miR319a.2*, and *miR408-5p* are all linked with H_2_O_2_-oxidative stress (Li et al., 2011). In an identical manner, Illumina sequencing revealed 29 known and 32 novel miRNAs to be differentially expressed upon salt stress in *Oryza glaberrima* (Mondal et al., 2018). Nonetheless, small RNA libraries sequenced from rice seedlings subjected to cadmium stress revealed a set of miRNAs, all of which contributed to stress regulation (Huang S.Q. et al., 2009; Ding et al., 2011). A report by Zhang et al. (2018) suggested that the *miRNA166* knockout rice mutants exhibited higher drought tolerance and smaller xylem diameter (Zhang et al., 2018). A recent study also used small RNA sequencing to identify *osa-miR12477* (Parmar et al., 2020). The *osa-miR12477* regulates the expression of *LAO* (L-ascorbate oxidase) for salt tolerance in the plant.

**TABLE 2:** List of miRNAs in rice trait improvement and stress management.

miRNA	Function	References
*miR156*	fast leaf/tiller initiation and advanced leaf maturation in rice; regulate the tillering-associated genes (*TB1, LAX1 and DWARF 53*)	Xie et al., 2012; Liu Q. et al., 2019
*miR172*	reduced seed weight, floral defects and delayed transition of spikelet meristem to floral meristem in rice	Zhu et al., 2009
*miR397*	grain size and panicle branching, eventually leading to increased rice grain production	Zhang et al., 2013
*miR159*	reduced size of organ, diameter of stem, length of flag leaf, size of grain panicle, and spikelet hulls	Zhao et al., 2017
*miR319*	suppressive effect on rice plant height	Liu et al., 2017
*miR396*	improved panicle branching and grain size	Zhang J. et al., 2020
*miR1432*	enhanced expression of *OsACOT* to promote grain filling	Zhao et al., 2019
*miR398*	modulate the expression of *Os-CSD1* and *Os-CSD2* as well as responses to abiotic and biotic stresses	Li et al., 2011
*miR169g*	Drought, salt, and blast fungus	Zhao et al., 2007, 2009; Jian et al., 2010; Zhou et al., 2010; Li et al., 2017
*miR393*	Drought, salinity, and cold	Lv et al., 2010; Gao et al., 2011; Xia et al., 2012; Lu et al., 2018
*miR169*, *miR397*, *miR528*, *miR827*, *miR1425*, *miR319a.2* and *miR408-5p*	H_2_O_2_-oxidative stress	Li et al., 2011
*miRNA166*	drought tolerance and smaller xylem diameter	Zhang et al., 2018
*miR12477*	regulates the expression of *LAO* (L-ascorbate oxidase) for salt tolerance	Parmar et al., 2020
*miR7695*	negatively controls an alternatively spliced transcript of *OsNRAMP6*, while its over-expression improves the resistance to *M. oryzae*	Campo et al., 2013
*miRNA169*	decreased rice immunity against *M. oryzae*	Li et al., 2017
*miRNA164a*	negatively regulates rice immunity against *M. oryzae*	Wang Z. et al., 2018

### Biotic Stress

Taking biotic stress into consideration, 13 and 16 blast resistance QTLs were recognized in Jin23B/CR071 and Jin23B/QingGuAi3 rice populations, respectively. The study revealed major and minor QTLs interactions as the basic genetic mechanism for blast resistance in CR071 and QingGuAi3 rice lines (Jiang et al., 2020). Lately, *pi 66(t)* was recognized as one of the recessive genes governing rice blast (Liang et al., 2016). Furthermore, the status and diversity of 12 major blast resistance genes were studied amongst 80 different rice varieties (Yadav et al., 2017). Molecular markers for genes *Pi54, Pib, Piz, Piz-t, Pik, Pi-kh, Pik-p, PikmPik-h, Pita/Pita-2, Pi2, Pi9, Pi1*, and *Pi5* were utilized in this investigation. Recently in *Oryza glumaepatula*, characterization of a wide effect QTL showed *Pi68(t)* as a potential gene for field resistance and neck blast in rice (Devi et al., 2020). Another very recent study focused on the meta-analysis of QTL with multiple disease resistance in rice (Kumar and Nadarajah, 2020). The study revealed *MQTL2.5, MQTL8.1, and MQTL9.1* have a significant count of *R*-genes which denotes 10.21, 4.08, and 6.42% of the total genes respectively. The defense-related genes contribute approximately 3.70, 8.16, and 6.42% of the total number of genes in *MQTL2.5, MQTL8.1*, and *MQTL9.1*, respectively. The study further led to the recognition of QTL hotspots for sheath blight, rice blast, and bacterial blight resistance. The potential gene candidates within these regions might be implemented for rice crop improvement via the intervention of genetic engineering.

With the increasing advent of high-throughput technologies, researchers have used microarrays and NGS/deep sequencing to accomplish genome-wide expression analysis to identify stress-regulated miRNAs (Table 2; Baldrich and San Segundo, 2016; Nadarajah and Kumar, 2019; Kar and Raichaudhuri, 2021). Xu et al. (2014) deployed microarray to study miRNA expression profiles in black-streaked dwarf virus (SRBSDV)-infected rice. They uncovered 56 miRNAs and 24 target genes to be potentially linked with diseased conditions. NGS was used to study small RNA expression profiles of rice seedlings infested with rice dwarf virus (RDV) and rice stripe virus (RSV). Campo et al. (2013) relied on high-throughput RNA sequencing to unravel a novel *osa-miR7695*. This miRNA negatively controls an alternatively spliced transcript of *OsNRAMP6* (*natural resistance-associated macrophage protein 6*), while its over-expression improves the resistance to *Magnaporthe oryzae* (Campo et al., 2013). In yet another study for the blast fungus *M. oryzae, miRNA169* was shown to inhibit the expression of its target nuclear factor Y-A genes. This resulted in decreased rice immunity against the pathogen (Li et al., 2017). A comparable effect was detected against the blast fungus with Osa-*miRNA164a* that targets *OsNAC60* gene (Wang Z. et al., 2018).

## Proteomics and Metabolomics: An Overview

Proteomics is a robust and powerful discipline that involves large-scale identification and quantification of proteins including, their structure and physiological functions. Precisely, proteome denotes a set or the entire complement of proteins within a cell, tissue, or organism. Proteome provides a data-rich panorama of regulation of expressed proteins under specific conditions. The word proteomics is an amalgamation of two words (protein and genome) and was first coined in 1994 by Mark Wilkins (Shah and Misra, 2011). Proteomics appendages the other omics techniques *i.e.*, genomics, transcriptomics, and metabolomics to cognize the function and structure of the protein of interest. Proteomics has proven to be a forte for the rice research community. Proteogenomics (large-scale proteome information is processed for genome annotation refinement) has greatly assisted in this direction (Helmy et al., 2011). Proteomes are available for almost all rice tissues and organs under normal or stressed conditions (Agrawal and Rakwal, 2011; Kim S.T. et al., 2014). Proteomics-based techniques are used in different capacities for crop improvement and deciphering environmental stress mechanisms. Nonetheless, the field of proteomics is exceedingly dynamic in nature due to the intricate regulatory systems governing the protein expression levels. Mass spectrometry (MS) with liquid chromatography (LC-MS-MS) and matrix-assisted laser desorption/ionization (MALDI-TOF/TOF) are central to current proteomics. The classical techniques for protein purifications involve ion-exchange chromatography (IEC), affinity chromatography, and size exclusion chromatography (SEC) (Agrawal et al., 2010; Agrawal et al., 2013). Enzyme-linked immunosorbent assay (ELISA) and western blotting are used for studying selective proteins (Yang and Ma, 2009; Kim et al., 2013). Sodium dodecyl sulfate-polyacrylamide gel electrophoresis (SDS-PAGE), two-dimensional differential gel electrophoresis (2D-DIGE), and two-dimensional gel electrophoresis (2-DE) techniques are routinely utilized for separation of complex protein mixtures (Choudhary et al., 2009; Pandey et al., 2010; Jaiswal et al., 2013). These techniques can be efficiently utilized to analyze a small set of proteins and are incapable of measuring protein expression levels. The technique 2-DE allows the study of differentially expressed proteins with the simultaneous detection and quantification of several protein spot isoforms, encircling post-translational modifications. Nevertheless, 2-DE based proteomics is biased against low abundance and hydrophobic proteins. For high-throughput protein expression analysis protein microarrays or chips have been established. However, it cannot be utilized to determine the function of complete proteome (Han et al., 2014a)., Edman degradation, MS, isotope-coded affinity tag (ICAT) labeling, stable isotope labeling with amino acids in cell culture (SILAC), multidimensional protein identification technology (MudPIT), and isobaric tag for relative and absolute quantitation (iTRAQ) are the few techniques for quantitative proteomic (Dong et al., 2014; Han et al., 2014b; Li Y. et al., 2014; Liao et al., 2014; Zhang et al., 2014; Li et al., 2015). Likewise, X-ray crystallography and nuclear magnetic resonance (NMR) spectroscopy are the main high-throughput technologies to determine the 3-D structure of a protein (Liao et al., 2014; Liu C.W. et al., 2014; Zhang et al., 2014). High-throughput data yields large quantities of proteomics data which is analyzed by various bioinformatics databases (Figure 1). Proteomics analysis in rice are grouped into gel-based (1-DE, 2-DE, and 2-DIGE), gel-free (LC-MS/MS, MudPIT, iTRAQ), and a coalescence of these two methods (Agrawal et al., 2013). Thus, proteomics enables to globally decipher the protein expression profiles and their analogous post-translational modifications.

Metabolomics is the systematic analysis of chemical processes including metabolites, substrates, intermediates, and products of cellular metabolism. Precisely, metabolomics involve the characteristic fingerprints that discrete cellular processes lay down resulting in a unique metabolic profile (Daviss, 2005; Gong et al., 2013). Genomics, transcriptomics, and proteomics reveal the expression pattern or cellular function of a gene within the cell. However, metabolomics offers a straight functional read-out of the physiological state associated with an organism (Daviss, 2005). The tools and techniques deployed for metabolomic data recording and processing have been proved to be more sophisticated than ever. The studies encircling the metabolome data have been long ramification of “hypothesis generator,” which remains a subject of further evaluation (Hall, 2006). Taking rice metabolomics in particular, it is a high-throughput technique to profile metabolites implicated directly or indirectly in metabolic processes (Figure 1). Further, it is widely deployed to monitor and evaluate the cellular metabolic state and quality of rice (Okazaki and Saito, 2016). Generally, metabolomics involves optional separation of small metabolites by gas chromatography (GC), high-performance liquid chromatography (HPLC), and liquid chromatography-mass spectrometry (LC-MS); followed with MS to identify and quantify metabolites. Crude extracts are utilized to profile metabolites in a non-targeted approach; thus, chromatographic separation is often essential to analyze fractionated compounds (Fukushima and Kusano, 2014). GC-MS is particularly the method of choice for the study of low molecular-weight metabolites. The process of chemical derivatization makes low molecular-weight metabolites acquiescent to GC. The major advantage of GC-MS is capacity metabolite profiling and targeted metabolite quantitation. This aids in the study of several metabolites in a single GC-MS-MS multiple reaction monitoring (MRM) run. MS is a stand-alone technique that is highly sensitive and specific. Alternately, the sample material with no prior separation is directly infused into the mass spectrometer. MS itself imparts adequate selectivity to separate and detect metabolites. Advanced techniques such as NMR, LC-MS, GC-MS, inductively coupled plasma (ICP)-MS, HPLC, and direct flow injection (DFI)-MS have significantly contributed to metabolic profiling (Uawisetwathana and Karoonuthaisiri, 2019). Fourier transform infrared spectroscopy (FTIR) is also popular in metabolomics for its capacity to simultaneously analyze and characterize intricate building blocks (Junot and Fenaille, 2019). Shortly, integrating metabolomics with genomics and proteomics has assisted in a proficient dissection of genetic, phenotypic, and protein level information in rice. Thus, the rice metabolome generates a “fingerprint” of diverse rice samples to ascertain the varieties that are crucial to rice trait improvement and stress management (Wei et al., 2018).

### Proteomics and Metabolomics in Rice Trait Improvement

One of the major traits associated with rice is the aroma. Two genes, *betaine-aldehyde dehydrogenase* (Bradbury et al., 2008) and *glyceraldehyde-3-phosphate dehydrogenase B form* (Lin et al., 2014) are involved with fragrance in rice. Moreover, aromatic rice has a flavor compound, 2-acetyl-1-pyrroline (2AP). Proteomic analysis of two isogenic lines of Thai jasmine rice was performed to gain insights into the 2AP biosynthetic pathway. 2-DE was performed on both isogenic lines which identified aldehyde dehydrogenase, a key enzyme responsible for 2AP production (Wongpia et al., 2016). Rice grains are also known to contain low quantities of storage proteins (glutelins, prolamins, albumins, and globulins). Few of them are allergens (α-amylase/trypsin inhibitor, globulins, β-glyoxylase, and glutelins). Proteins from 4 different rice varieties were analyzed by 2D-GE. Further investigation revealed, few of the differentially abundant proteins as allergenic proteins. Particularly, a deletion in the 1000 bp upstream region of the *globulin* gene has been recognized, probably contributing to the varied abundance of the protein in the Karnak cultivar. This is useful for cultivar identification in commercial samples (Graziano et al., 2020). In another interesting study, three cytochrome P450 homoeologs (*Os03g0603100*, *Os03g0568400*, and *GL3.2*) and *OsBADH2* were edited with the CRISPR/Cas9 to produce novel rice mutants. Evidently, CRISPR/Cas9 has revolutionized the arena of plant sciences (Iqbal et al., 2020a). The mutants exhibited elevated yields and enhanced aroma. RNA-seq and proteomic analysis were done to unravel the underlying modifications. Mutants showed increased grain size, grain cell number, and high 2AP content. RNA sequencing and proteomic analysis showed the involvement of genes and proteins linked to the cytochrome P450 family, grain size and development, and cell cycle (Usman et al., 2020). Anthocyanin and proanthocyanin are flavonoids that are present in good quantities in black and red rice. To decipher the molecular pathways, a study was performed to understand the flavonoid biosynthetic pathway in red, black, and white colors rice cultivars. A comprehensive profile of mRNA and expressed proteins in diverse colored rice varieties was obtained by RNA sequencing of caryopsis and iTRAQ analysis. A total of 3417, 329, and 227 genes were distinctive for red, white, and black rice, respectively. Furthermore, the proteomes of black, white, and red rice contained 13,996 distinctive peptides corresponding to 3916 proteins. Interestingly, 32 genes were shown to be implicated in the flavonoid biosynthesis pathway. From those 32 genes, only *CHI*, *F3H*, *ANS*, and *FLS* were ascertained by iTRAQ (Chen et al., 2019). A similar study on two black rice cultivars (BALI and Pulut Hitam 9), two red rice cultivars (MRM16 and MRQ100), and two white rice cultivars (MR297 and MRQ76) using label-free liquid chromatography Triple TOF 6600 tandem mass spectrometry (LC-MS-MS) was conducted. The study profiled and ascertained the proteins associated with nutritional values (antioxidant, folate, and low glycemic index) and quality (i.e., aromatic) based on peptide-centric scoring from the Sequential Window Acquisition of All Theoretical Mass Spectra (SWATH-MS) approach (Sew et al., 2020). Recently, the effect of germination (post 24 h) on nutrition-associated proteins in 4 rice cultivars was studied using shotgun proteomics. In-gel digestion coupled with tandem mass spectrometry (GeLC-MS/MS) was performed on 4 rice cultivars to analyze the total proteins from non-germinated seeds and 24 h germinated seeds. Total phenolic content was also measured post 0, 24, and 48 h of germination by Folin-Ciocalteu assay. The study revealed that seed nutrition-related proteins, particularly phenolic proteins increased post-germination. A 2.20 – 15.90 folds increment in the expression of phenylalanine ammonia-lyase, serine carboxypeptidase-like protein, isoflavone-7-O-methyltransferase, isoflavonoid glucosyltransferase, glycosyltransferase family 61 protein, and UDP-glucose flavonoid 3-O-glucosyltransferase was observed post-germination. The study supported the notion that rice germination for 24 h influences the enhanced nutrition of brown rice and the phenolic biosynthetic pathway (Maksup et al., 2020).

Genetic engineering intervention in the generation of genetically modified rice cultivars is well illustrated by “Golden rice” (Khan et al., 2015). Wild-type rice is devoid of vitamin A or its precursor-beta-carotene. Its deficiency affects the human population that consumes rice as a staple food. The rice genome was genetically engineered with a multi-gene biochemical pathway to synthesize beta-carotene that is eventually metabolized by humans to synthesize vitamin A (Baranski, 2013; Khan et al., 2015). Pleiotropic effects, mutation, and inactivation of endogenous genes are the basis for the generation of such cultivars with unintended phenotypes (Matsaunyane and Dubery, 2018). Genetic alteration of *phytoene synthase* (*Psy*) and *phytoene desaturase* (*crtI*) that leads to metabolic regulation and adaptation of “golden rice” has been extensively studied (Gayen et al., 2016). Transgenic and non-transgenic seeds of golden rice were collected for proteomic and metabolomic studies. HPLC analysis identified significantly high levels of carotenoids in the transgenics. The higher level of carotenoid in the transgenics is attributed to *Psy* and *crtI* expressions. Also, the GC-MS approach was deployed to detect the changes in the carbohydrate metabolism pathway in the transgenics (Decourcelle et al., 2015). The transgenics accumulated higher amounts of galactose, fructo furanose, D-glucoronate, and D-sorbitol. Surprisingly, the proteomic results were found to be in correlation with the metabolomic data as greater activities for enzymes (pullulanase and UDP-glucose pyrophosphorylase) were found in the transgenics. These enzymes are imperative to carbohydrate metabolism and are linked with the biosynthesis of carotenoids (Chen et al., 2007; Gayen et al., 2016). Additionally, the activity of pyruvate phosphate dikinase implicated in pyruvate biosynthesis (precursor of carotenoid) was also found to be higher in the transgenics (Gayen et al., 2016). Song J.M. et al. (2018) conducted an interesting study on rice leaves and grains to further accentuate the role of metabolomics in rice research (Song E.H. et al., 2018). The metabolic profile of two rice cultivars (early maturing rice cultivar- EMC and late maturing rice cultivar- LMC) was assessed by an NMR- based metabolomics (^1^H NMR). Distinct metabolic profiles in leaves and grains at all growth stages of EMC and LMC were detected. For rice grains, significantly elevated levels of sucrose, amino acids, and fatty acids were observed in EMC than LMC. Thus, the nutritional value in EMC rice grains was higher than LMC rice grains (Song E.H. et al., 2018). In a recent study, phenolics, especially flavonoids and antioxidants in two rice varieties (*Oryza sativa*-Os and *Zizania latifolia*-Zl) were studied. A UHPLC-QqQ-MS-based metabolomics approach revealed that Zl possessed higher levels of phenolics, flavonoids, proanthocyanidins, and antioxidant activity. Out of 159 identified flavonoids, 78 showed differential expression (72 up-regulated and 6 down-regulated in the Zl). The majority of flavonoids in Z1 were related to anthocyanin biosynthesis owing to its better nutrition profile (Yu et al., 2021). A more holistic study on rice metabolomics involved 17 cultivars from 7 different countries (Zarei et al., 2018). The group of metabolites and metabolome significantly varied amongst the cultivars. On average, 411 metabolites per cultivar were annotated and 71 metabolites were different between them. Prior, a similar study depicting the disparities between *indica* and *japonica* sub-species had been conducted (Hu et al., 2014). Among the 92 significantly variable metabolites, 66 were up-regulated in *japonica* while 26 were up-regulated in *indica* cultivars. Asparagine had higher quantities in the *indica* sub-species and was regarded as the most variable of all the metabolites according to the Random Forest ranking. The metabolites of interest demarcating the two sub-species were associated with nitrogen metabolism, translocation, inorganic nutrition storage, and stress responses. Trait-associated metabolites with respect to biosynthetic and catabolic pathways will deepen the knowledge toward rice trait improvement (Table 3). Yet another study focused on the pathways related to the aroma in fragrant rice (Daygon et al., 2017). As stated earlier, 2AP in rice is a pivotal aroma compound. The analysis by Daygon et al. (2017) using GC × GC-TOF-MS showed 6-methyl, 5-oxo-2,3,4,5-tetrahydropyridine (6M5OTP), 2-acetylpyrrole, pyrrole and 1-pyrroline were related with the synthesis of 2AP in aromatic rice cultivars. Further, the GWAS indicated that all the above 4 compounds were linked with a single QTL that harbors the *FGR* gene linked with GABA production (Daygon et al., 2017). Recently, GC-MS based approach has also been used to assess rice grain quality through profiling of volatiles and metabolites in rice grains (Llorente et al., 2019). Thus, proteomics and metabolomics have contributed significantly in comprehending the underlying pathways and compounds associated with rice trait improvement.

**TABLE 3:** List of proteomics and metabolomics techniques in rice trait improvement and stress management.

Method	Key finding	References
2-DE	proteins related with aroma compound biosynthesis	Wongpia et al., 2016
2D-GE	Development of a molecular marker useful for the analysis of commercial products	Graziano et al., 2020
HPLC Fractionation and LC-MS/MS Analysis	cytochrome P450 family, grain size and development, and cell cycle	Usman et al., 2020
iTRAQ analysis	flavonoid biosynthesis pathway	Chen et al., 2019
LC-MS-MS and SWATH-MS	Nutritional and quality traits	Sew et al., 2020
GeLC-MS-MS	enhanced nutrition of brown rice and the phenolic biosynthetic pathway	Maksup et al., 2020
GC-MS	carbohydrate metabolism pathway	Decourcelle et al., 2015
GC-MS	endosperm specific carotenoid pathways	Gayen et al., 2016
^1^H NMR	intrinsic physiology and potential eating quality	Song E.H. et al., 2018
UHPLC-QqQ-MS	better nutrition profile	Yu et al., 2021
UPLC-MS-MS	rice gene–bran metabolite relationships	Zarei et al., 2018
UPLC-MS-MS and GC-MS	Metabolic variation between japonica and indica rice cultivars	Hu et al., 2014
GC × GC-TOF-MS	Aroma in fragrant rice	Daygon et al., 2017
GC-MS	rice grain quality through profiling of volatiles and metabolites	Llorente et al., 2019
Tandem mass tagging and Nano-LC-MS/MS	involvement of JA under drought at molecular level	Dhakarey et al., 2017
LC-MS-MS	photosynthesis-related adaptations via NADP(H) homeostasis to drought	Chintakovid et al., 2017
nLC-MS-MS	Drought stress	Hamzelou et al., 2020
LC-MS-MS	Rice growth and grain yield with heavy nitrogen application before and after drought	Du J. et al., 2020
2-DE and MALDI TOF	seed imbibition under salt stress	Xu et al., 2017
iTRAQ	early salinity response	Lakra et al., 2019
LC-MS-MS	Rice yield under salinity stress	Li et al., 2020
1D LDS-PAGE, in-gel digestion, and LC-MS-MS	cold tolerance response	Lee et al., 2017
2-DE and MALDI-TOF-MS	cold tolerance response	Ji et al., 2017
nLC-MS-MS	cold tolerance response	Wang J. et al., 2018
^1^H NMR and GC-MS	drought tolerance via GABA biosynthesis, sucrose metabolism and antioxidant defenses.	Nam et al., 2016
GC-MS	drought and heat stress in the field	Lawas et al., 2019
GC-MS	metabolic pathways associated with photosynthesis upon drought exposure	Ma et al., 2016
GC-MS	Salinity stress	Xie et al., 2020
GC-MS	Salinity stress	Gupta and De, 2017
GC-TOF-MS	Salinity stress	Wanichthanarak et al., 2020
LC-MS-MS	rice metabolic network underlying *OsDRAP1*-mediated salt tolerance	Wang et al., 2021
EESI-MS	Cold stress	Du S. et al., 2020
2D Nano LC MS-MS, 2-DE, In-gel digestion and MALDI-TOF MS	accumulation of photosynthesis and defense associated proteins by *Pseudomonas fluorescens* and *Sinorhizobium meliloti*	Kandasamy et al., 2009; Chi et al., 2010
UHPLC-DAD/ESIQTOF	metabolomics signatures such as decreased alkylresorcinol quantities and the differential induction of N-p-coumaroylputrescine and N-feruloylputrescine (antimicrobial compounds)	Valette et al., 2020
HPLC	exudation of salicylic acid	Kandaswamy et al., 2019
LC-MS/MS	*Pseudomonas aeruginosa* is linked with the synthesis of SAR related compounds such as siderophores and antibacterial compounds	Yasmin et al., 2017
iTRAQ	regulatory response to *M. oryzae* in durable resistant vs. susceptible rice genotypes	Ma Z. et al., 2020
QTOF-UPHPLC	Bayogenin 3-O-cellobioside confers non-cultivar-specific defense against *Pyricularia oryzae*	Norvienyeku et al., 2021
2-DE and MALDI-TOF-MS-MS	accumulation of novel proteins potentially involved in defense against *Rhizoctonia solani*	Karmakar et al., 2019
iTRAQ	rice immunity to infection by *R. solani*	Ma H. et al., 2020
Tandem mass tagging with LC-MS/MS	interaction between rice and *X. oryzae*	Zhang F. et al., 2020
iTRAQ	defense response to *M. oryzae* in rice with blast resistance gene *Piz-t*	Tian et al., 2018

### Proteomics and Metabolomics in Stress Management:

Proteomics and metabolomics-based studies are expected to improve rice plant responses toward fluctuating environmental conditions. In the past few years, the contribution of omics sciences has been immense in rice research for studying the pathways, metabolites, and proteins involved in combating stress. Some case studies with respect to abiotic and biotic stresses are discussed below.

### Abiotic Stress

The application of proteomics in rice stress management includes the study of physiological and proteomic analysis of the rice mutant *coleoptile photomorphogenesis 2* (*cpm2-*disrupted in allene oxide cyclase). The study revealed negative regulation of jasmonic acid (JA) in drought tolerance (Dhakarey et al., 2017). Tandem mass tagging and Nano-LC-MS-MS was performed to comprehend the involvement of JA under drought at the molecular level. The histological, metabolite and proteome-based transcript analysis revealed the favorable adaptations and responses against drought stress, mainly coordinated by the absence of JA in the *cpm2* roots (Dhakarey et al., 2017). In a similar vein, proteomic analysis of drought-responsive proteins by LC-MS-MS revealed photosynthesis-related adaptations via NADP(H) homeostasis to drought (Chintakovid et al., 2017). Recently, 8 genotypes of *japonica* and *indica* sub-species at the late vegetative stage were studied with nano LC-MS-MS (nanoflow liquid chromatography-tandem mass spectrometry) for drought stress (Hamzelou et al., 2020). Label-free quantitative shotgun proteomic analysis of 8 rice genotypes subjected to drought unraveled 1253 non-redundant proteins under well-watered and drought conditions. In all the 8 genotypes, 8 proteins were induced under drought stress (Hamzelou et al., 2020). A more comprehensive study by Du J. et al. (2020) involved proteomics, metabolomics, and physiological analyses upon heavy nitrogen exposure before (NBD) and after drought (NAD) on rice (Du J. et al., 2020). The proteomic experiments were carried by tandem mass tagging of rice leaves subjected to NBD and NAD. The samples were analyzed by LC-MS-MS with the amount of qualitative protein and quantitative protein being 4254 and 3892 respectively. Upon drought exposure, NBD had higher chlorophyll content and photosynthetic rate, enhanced activities of antioxidant enzymes such as superoxide dismutase (SOD), peroxidase, and catalase, and declined malondialdehyde (MDA) content (Du J. et al., 2020). Next, the application of multi-omics in salinity stress involves an analysis by Xu et al. (2017) utilizing the techniques 2-DE and MALDI TOF (Xu et al., 2017). The relative proteomic analysis was performed amongst the dry and imbibed seeds of salt-tolerant *japonica* landrace Jiucaiqing with 150 mM NaCl. A total of 14 proteins were identified to be implicated in seed imbibition. Many of the identified proteins were involved in energy supply and storage. Upon analysis, 2,3-bisphosphoglycerate-independent phosphoglycerate mutase (*BPM*), glutelin (*GLU2.2* and *GLU2.3*), glucose-1-phosphate adenylyltransferase large subunit (*GAS8*), and cupin domain-containing protein (CDP3.1 and CDP3.2) were close to QTLs for seed dormancy, seed reserve utilization, and seed germination. Interestingly, *CDP3.1* co-localized with *qIR-3* for imbibition rate. The study further established the function of *CDP3.1* in regulating seed germination upon salinity stress (Xu et al., 2017). Later, iTRAQ was deployed to analyze the disparities in the proteome of salt-sensitive (IR64) and salt-tolerant (Pokkali) seedlings upon salt exposure (Lakra et al., 2019). Significantly higher levels of proteins implicated in photosynthesis (oxygen evolving enhancer proteins OEE1 and OEE3, PsbP) and stress tolerance (ascorbate peroxidase, SOD, peptidyl-prolyl cis-trans isomerases, and glyoxalase II) were found in the shoots of Pokkali. Upon salinity exposure, ribulose bisphosphate carboxylase/oxygenase activase and glutamate dehydrogenase were found to be highly induced in Pokkali (Lakra et al., 2019). Further, Li et al. (2020) performed a shotgun proteomic analysis of germinated rice under salinity conditions. Seven Thai rice cultivars (Pathumthani, Phitsanulok2, RD31- salt tolerant cultivars; RD29, RD41, Riceberry- moderately salt tolerant cultivars; and RD47- salt susceptible cultivar) were germinated under 200 mm NaCl for 96 h. Shotgun proteome analysis from all the seven cultivars identified 1339 proteins. A total of 51 proteins (involved in protein modification, signal transduction, stress response, transport, and transcription) were exclusively expressed only in salt tolerant cultivars (Li et al., 2020). Shotgun proteome analysis was also done on rice anthers from a cold-tolerant variety, Dianxi 4. Normal anthers and cold exposed anthers at the young microspore stage were compared for protein expression. A total of 3835 non-redundant proteins were detected, of which 441 proteins were expressed differentially. The study identified C2 domain proteins, and GRPs as promising signaling factors for cold tolerance response (Lee et al., 2017). A more holistic proteomic study on rice seedlings subjected to cold stress was performed using 2-DE and MALDI-TOF-MS on cold sensitive line 9311 and cold tolerant variety Fujisaka 5. In total, 59 proteins associated with cold resistance were observed in this study (Ji et al., 2017). Moreover, cold-sensitive cultivar 9311 and cold-resistant hybrid wild rice DC907 with a 9311 genetic background were utilized to perform quantitative proteomic analysis with tandem mass tags. In DC907, 366 distinct proteins were identified which were primarily implicated in ATP synthesis, photosystem, reactive oxygen species (ROS), stress response, cell growth, and integrity (Wang J. et al., 2018).

Nuclear magnetic resonance analysis was used to evaluate the metabolomic changes in watered and drought-exposed transgenic rice grains. A demarcating metabolic profile was observed under different watering conditions in transgenic and wild-type rice grains. Upon drought exposure, significantly elevated levels of GABA (244.6%), fructose (155.7%), glucose (211.0%), glycerol (57.2%), glycine (65.8%), and aminoethanol (192.4%) were found in the transgenics (Nam et al., 2016). GABA is one of the pivotal metabolites often linked to abiotic stresses in rice. It is known to induce oxidative injuries in rice arising due to various stresses such as osmotic, salinity, or senescence (Ansari et al., 2005; Sheteiwy et al., 2019). The role of GABA in stress regulation has been recently reviewed extensively (Ansari et al., 2021; Khan et al., 2021). Similar to the NMR-based approach, a GC-MS-based metabolomics approach was deployed to study the metabolite profile of rice cultivars at different developmental stages under drought and heat conditions. More than 50% of identified metabolites were different in two of the three cultivars (Anjali’, Dular, and N22). The drought, heat, and combined drought and heat susceptible-Anjali; the drought, heat, and combined drought and heat tolerant- N22; the drought tolerant, heat and combined drought and heat susceptible-Dular were analyzed for drought and heat responses (Lawas et al., 2019). A GC-MS metabolomic approach along with transcriptome analysis was also used to study the key metabolic pathways associated with photosynthesis upon drought exposure. The study was designed on drought-sensitive cultivar IRAT109 and the drought-tolerant cultivar IAC1246 to determine the transcript and metabolic responses upon long-term drought exposure (Ma et al., 2016). For recent metabolic studies encircling salt stress in rice, GC-MS was utilized to profile metabolites in five rice varieties with a comparable genetic background and varying growth performances under salt stress. The study showed enriched levels of amino acids in salt-tolerant lines (G58, G1710, and IR64) in comparison to salt sensitive lines (G45 and G52) under non-stress conditions. In all five varieties, the levels of Sorbitol, melezitose, and pipecolic acid were enhanced significantly upon salinity stress. This probably indicated that these compounds might be responsible to regulate salt stress responses in rice. Moreover, the sensitive varieties experienced more noticeable enhancement in metabolites levels during early stress treatment in comparison to the tolerant varieties (Xie et al., 2020). An analogous study by Gupta and De (2017) revealed similar results upon salt stress in rice. The study employed GC-MS for assessing the metabolic profile; and found serotonin and gentisic acid as the key metabolites (Gupta and De, 2017). In yet another study, metabolomics (GC-TOF-MS) and transcriptomics (RNA-seq) were jointly utilized to decipher pathways, metabolites, and metabolic hotspots in rice upon salinity stress (Wanichthanarak et al., 2020). A very recent report also combined metabolomic (LC-MS-MS) and transcriptomic (RNA-seq) approaches to study the rice metabolic network underlying *OsDRAP1*-mediated salt tolerance (Wang et al., 2021). Over-expressing *OsDRAP1* results in differential expression of intrinsic salt tolerance genes. Moreso, proline, valine glyceric acid, phosphoenolpyruvic acid, and ascorbic acid accumulated at higher concentrations in the over-expressing lines, depictive of their role in salinity tolerance. Much alike drought and salt stress, the implementation of metabolomics is also extended to cold stress (Zhao et al., 2013). A recent study in this context involves electrospray ionization mass spectrometry (EESI-MS) to profile the metabolic changes of Qiutianxiaoting (chilling-tolerant variety) and 93-11 (chilling-susceptible variety) under low-temperature stress (Du S. et al., 2020). The study revealed that phenylpropanoid biosynthesis, flavone, and flavonol biosynthesis pathways were activated in 93-11 upon low-temperature exposures. In Qiutianxiaoting, low-temperature exposures activated methyl jasmonate biosynthesis-associated genes, which probably mitigated the chilling damage making it the more tolerant cultivar (Du S. et al., 2020).

### Biotic Stress

Metabolomics and proteomics of rice biotic stress at their homeostasis or adverse environmental condition is used to extract system information. The underpinning mechanisms of biotic stress responses in rice are well elucidated by targeted biochemical, metabolic, and proteomic analysis of host-pathogen interactions (Ahuja et al., 2012; Liu Y. et al., 2019; Vo et al., 2021). In view of this several relevant studies have been made. Plant growth-promoting rhizobacteria (PGPR) aids plants in nutrient uptake and phytohormone synthesis. Early studies involving proteomics revealed photosynthesis and defense associated proteins accumulation by *Pseudomonas fluorescens* and *Sinorhizobium meliloti* (Kandasamy et al., 2009; Chi et al., 2010). Similarly, an early metabolomics study encircling PGPR was performed on two rice varieties infested with *Azospirillum lipoferum* 4B and *Azospirillum* sp. B510 (rice-associated *Azospirillum* species). The study found alterations in flavonoids and hydroxycinnamic derivatives which were predominantly dependent on the cultivar-PGPR strain interaction (Chamam et al., 2013). Moreover, 10 different PGPR strains inoculation of Nipponbare resulted in metabolomics signatures such as decreased alkylresorcinol [5-tridecyl resorcinol, 5-pentadecyl resorcinol, 5 (12-heptadecyl) resorcinol] quantities and the differential induction of N-p-coumaroylputrescine and N-feruloylputrescine (antimicrobial compounds) (Valette et al., 2020). Additionally, *Pseudomonas* is a known PGPR that acts as a bioagent to combat rice diseases. HPLC of rice roots infested by *Pseudomonas putida* revealed enrichment of salicylic acid (Kandaswamy et al., 2019). Likewise, *Pseudomonas aeruginosa* is linked with the synthesis of systemic acquired resistance (SAR) related compounds such as siderophores (1-hydroxy-phenazine, pyocyanin, and pyochellin) and antibacterial compounds (4-hydroxy-2-alkylquinolines and rhamnolipids) (Yasmin et al., 2017). The recent proteomics and metabolomics researches encircling rice response to disease causing pathogens have been intensively reviewed (Azizi et al., 2019; Meng et al., 2019). Metabolomics mostly highlighted the disparities of necrotrophic and biotrophic stages which included the accretion of metabolic photosynthetic compounds at biotrophic stage or phenolic compounds at necrotrophic stage (for review, see Azizi et al., 2019). A neoteric study for rice blast iTRAQ revealed that the pathogen-associated molecular pattern (PAMP)-triggered immunity might be induced at the transcriptome level but was suppressed at the protein level in susceptible rice varieties (Ma Z. et al., 2020). The study also revealed that probenazole-inducible protein 1 (PBZ1) and phenylpropanoid accumulated in both resistant and susceptible cultivars (Ma Z. et al., 2020). Intriguingly, a QTOF-UPHPLC based metabolomic study found a saponin, Bayogenin 3-O-cellobioside as a novel saponin identified in rice (Norvienyeku et al., 2021). Consequently, Bayogenin 3-O-cellobioside is well related with rice blast resistance against *Pyricularia oryzae.*

Sheath blight in rice is triggered by a necrotrophic fungus-*Rhizoctonia solani*, which is linked with cell death at the early stages of infection. Photosynthesis and sugar metabolism alters drastically upon *Rhizoctonia solani* infection (Lee et al., 2006). Further, two other metabolomic reports revealed the elevated levels of glycolysis and TCA cycle compounds (succinate, pyruvate, and aconitate), reduced levels of sugar (sucrose, glucose, fructose, glucosone, turanose, galactose, hexopyranose, maltose, and glucopyranose), accumulation of ROS, salicylic acid, jasmonic acid, aromatic aliphatic amino acids, phenylpropanoids, and suppression of myo-inositol (Suharti et al., 2016; Ghosh et al., 2017). Additionally, Karmakar et al. (2019) performed 2-DE and MALDI-TOF-MS-MS on control and *AtNPR1-*transgenics before and after *R. solani* infestation to study the proteome and metabolome profiles (Karmakar et al., 2019). Mitogen-activated protein kinase 6, probable protein phosphatase 2C1, probable trehalose-phosphate phosphatase 2, and heat shock protein were primarily recognized as the main compounds related to *R. solani* infection in rice. Moreover, the iTRAQ technique highlighted the difference in ROS modulation between the tolerant and susceptible varieties (Ma H. et al., 2020). The proteins were implicated in the regulation of glyoxylate and dicarboxylate metabolism, glycine, serine, and threonine metabolism, unsaturated fatty acid biosynthesis, and glycolysis/gluconeogenesis pathways. Several studies have investigated the differences in rice proteomes after challenging two major rice pathogens; *M. oryzae*, *Xanthomonas oryzae*, and/or their elicitors (Jha et al., 2007; Pandey and Sonti, 2010; Wu et al., 2016; Meng et al., 2019). For example, iTRAQ analysis was performed to study rice blast using *Piz-t* transgenic lines (*Piz-t*; rice blast *R* gene). Comparative proteome profiling on the *Piz-t* transgenic Nipponbare line (NPB-Piz-t) and wild-type Nipponbare (NPB) revealed differentially expressed proteins related to defense, stress, hormone, pathogenesis, and cytochrome P450 (Tian et al., 2018). Similarly, comparative proteomic profiling highlighted novel insights into the interaction between rice and *X. oryzae* (Zhang F. et al., 2020). The above examples constitute a few of the contemporary developments made by proteomics and metabolomics in response to abiotic/biotic stresses (Table 3).

## Databases for Rice Omics Research

The omics data sources include whole-genome sequencing data, RNA-sequencing data, protein-protein interaction data, and whole metabolome analysis data. Systematic accessibility, retrieval, and storage of omics data is the fundamental prerequisite for rice research. Omics-based research generates massive volumes of data that coincides with bioinformatics for meaningful processing of biological information. Accordingly, the subject of prime importance in molecular biology is how proficiently large volumes of data can be processed to retrieve meaningful information. This underlines the extreme need for molecular biology databases. Omics-based databases are not just the assembly of data in a system, but a platform from which information can be searched easily and quickly. Efficient molecular biology databases usually have the following functionality. First, data is linked to other meaningful information. For instance, sequence information linked to genetic resources can assist in genome-wide studies. Second, the search is intuitive and is key-word based. Third, large volumes of data can be downloaded easily without errors. Apart from the above features, open access is also an important requirement. Open access helps the users to browse and download the same data multiple times without any charges. Thus, data download, upload, and accessibility are essential for biological databases.

### Genomics and Transcriptomics Bioinformatics Tools and Databases

The availability of rice genome sequencing data from several species and cultivars has led to enormous research encircling the biological diversity of rice (Li J.Y. et al., 2014). Many tools and databases are established over the years to store, retrieve, and interpret big omics data. Extensive genome databases have been developed since the establishment of the Rice Genome Annotation Project (RGAP) (Ouyang et al., 2007) and Rice Annotation Project Database (Ohyanagi et al., 2006; Sakai et al., 2013). The functional genomics of rice is often studied with the OryGenesDB (Droc et al., 2006) and rice functional genomics express database (RiceGE). Both these databases utilize flanking sequence tag (FST) information for genome interpretation. Similarly, the RiceGE database provides relevant information on mutants. To gain access to genome data for various cultivars, the ricepan-genome browser (RPAN) (Sun et al., 2017) and Rice Information Gateway (RIGW) (Song J.M. et al., 2018) are prevalently used. Additionally, the Information Commons for Rice (IC4) database provides data regarding sequence variation and transcriptome profiles. For GWAS studies, HapRice- an SNP haplotype database (Yonemaru et al., 2014) and Ricebase- genome information platform for molecular markers such as SSRs (Edwards et al., 2016) are routinely deployed by bioinformaticians. Few other databases for SNP searches include OryzaGenome v2 (Ohyanagi et al., 2016), RiceVarMap (Zhao et al., 2015), and the SNP-Seek database (Alexandrov et al., 2015). GWAS data is often converted into a high-density rice array (HDRA) to cover 39,045 non-transposable elements in rice (McCouch et al., 2016). A Manhattan plot for the HDRA data is generated by GWAS viewer. This kind of analysis generally requires programming skills. However, graphic user interface (GUI) interface platforms such as Intelligent Prediction and Association Tool (iPat) (Chen and Zhang, 2018) and the rice imputation server (Wang D.R. et al., 2018) are also available for GWAS studies.

In the past few years, transcript-assembly algorithms have revolutionized the arena of rice transcriptomic research. Generally, the databases dedicated to transcriptome provides information regarding genome-wide expression profiles. An extremely important database in this context is OryzaExpress (Hamada et al., 2011). This database contains expression data from 1206 samples of 34 experimental series of GPL6864 (Agilent 4 × 44K microarray platform) and 2678 samples of 153 experimental series of GPL2025 (Affymetrix Rice Genome Array platform). In addition, Rice Oligonucleotide Array Database (ROAD) contains 1867 publicly available rice microarray data (Cao et al., 2012). Another database named Collections of Rice Expression Profiling database (CREP) provides access to data from 190 Affymetrix GeneChip Rice Genome Arrays from 39 tissues (Wang et al., 2010). The information regarding rice field/development, plant hormone, and cell/tissue type can be retrieved from the RiceXpro database (Sato et al., 2013a, b). Additionally, the uniformed viewer for integrated omics (UniVIO) database can be utilized to analyze 43 hormone-related compounds (Kudo et al., 2013). For biotic stress-related studies in rice, the plant expression database (PlexDB) (Dash et al., 2012) and EXPath database (Chien et al., 2015) are commonly used. Likewise, EXPath provides tissue/organ specific expression, gene ontology (GO), and Kyoto Encyclopedia of Genes and Genomes (KEGG) pathway analysis for six model crops, including rice. The Rice eFP browser (Winter et al., 2007) intuitively displays expression values using color gradients. Large volumes of rice mRNA sequencing data in different conditions is also accessible via the Transcriptome Encyclopedia of Rice database (Kawahara et al., 2016). Moreso, the Rice Expression Database (RED) is a reservoir of gene expression profiles from different rice tissues under varying environmental conditions (Xia et al., 2017). Together with RED, Expression Atlas is often used to access the gene expression profiles from recent researches (Papatheodorou et al., 2018). Genevestigator is yet another database that allows curation, visualization, and analysis of microarray or RNA sequencing data (Hruz et al., 2008). Nonetheless, large volumes of transcriptome data from different tissues or under different conditions also allow co-expression analysis. PlantArrayNet (Lee et al., 2009), the plant co-expression database (Yim et al., 2013), and the CoP database (Ogata et al., 2010) are useful for rice co-expression studies. These web-based tools use a standard pipeline to offer useful knowledge about the genes co-expressed with a gene of interest. Apart from these tools, the ATTED-II database provides co-regulated gene relationships to deduce gene functions (Obayashi et al., 2018). Additionally, NetMiner is a standalone tool for exploratory analysis and visualization of network data (Yu et al., 2018). The transcriptome analysis is frequently coupled with promoter analysis for identifying the *cis*-regulatory elements. The representative databases to study the motif organization include plant *cis*-acting regulatory DNA elements (PLACE) (Higo et al., 1999), plant *cis*-acting regulatory elements (PlantCARE) (Lescot et al., 2002), plant promoter database (PPDB) (Yamamoto and Obokata, 2007), and plant promoter analysis navigator (PlantPAN) (Chow et al., 2016). A rice-specific promoter analysis database is the Osiris database (Morris et al., 2008). It is a repository for promoter sequences and probable TF binding sites for 24,209 rice genes. Although it has now become obsolete. However, the MEME suite (Bailey et al., 2009) is now generally the choice of researchers for performing web-based motif identification. Further, several databases have been developed lately to dissect the gene expression patterns regulated by non-coding RNA (ncRNA). The pyrosequencing generated small-RNA sequences for rice and maize are routinely accessed via the Cereal Small RNA Database (Johnson et al., 2007). For miRNA-based studies, plant non-coding RNA database (PNRD) (Yi et al., 2015) and miRBase (Kozomara et al., 2019) are dedicated data resources. PNRD stores data from 166 plant species to generate valuable information regarding miRNAs, intronic long ncRNAs (lncRNA), and unknown ncRNAs. Likewise, the miRbase contains miRNAs information from 271 organisms. The rice miRNA information can be accessed on miRbase with a file named osa.gff3. On similar lines, annotations of 287 eukaryotic lncRNAs are provided by the Long Non-coding RNA database (lncrnadb) (Quek et al., 2015). The data for multiple miRNA variants from eight species, including rice is provided by IsomiR bank database (Zhang et al., 2016). Moreover, the plant ceRNA database (PceRBase) (Yuan et al., 2017) and plant circular RNA database (PlantcircBase) (Chu et al., 2017) cover information regarding competing endogenous RNA (ceRNA) and circular RNA (circRNA) respectively.

### Proteomics and Metabolomics Bioinformatics Tools and Databases

In context to systems biology, protein-protein interactions are pivotal to large complex networks (Rao et al., 2014). Several interactome datasets have been hosted to study the protein-protein interactions in rice. Such resources are distinct in relation to number of interactions, source of the embedded interactome, and accessible organisms. One of the methods for envisaging protein-protein interactions is the interolog approach. According to interolog approach, the function of a protein is conserved and passed through its orthologs in evolutionary-related species. Thus, the orthologs of interacting proteins in one organism conserve their interactions in a different organism. Based on the interolog approach, 37112 interactions amongst 4567 proteins are summarized approach by the Rice Interactions Viewer (RIV) database. Amongst these interactions, 1671 are self-interactions while 35441 are hetero-interactions (Ho et al., 2012). The predicted rice interactome network (PRIN) is yet another rice database that uses interolog approach (Gu et al., 2011). It annotates 76585 non-redundant rice protein interaction pairs amongst 5049 rice proteins. Meaningful interactions are validated by PRIN upon fetching the gene expression data, sub-cellular localization information, and GO annotation. Additionally, the database of interacting proteins in *Oryza sativa* (DIPOS) uses the interolog approach and domain-based predictions to depict the protein-protein interactions. This database hosts 14614067 pairwise interactions amongst 27746 proteins (Sapkota et al., 2011). Further to outspread the interactome, several approaches namely text-mining, neighborhood analysis, co-expression analysis, fusion analysis, and co-occurrence analysis are prevalently deployed (Szklarczyk et al., 2016). For extensive interactome coverage, the STRING database utilizes a broad range of sources available, from text-mining to computational predictions (Szklarczyk et al., 2016). STRING database provides both predicted and indirect interactions networks, where the nodes represent the proteins while the edges are the predicted functional association. The information for 2031 organisms is present on the STRING database. One of the latest versions of STRING (v10.5) supports network connections for 26428 *japonica* proteins and 18789 *indica* proteins. Interactions are based on combined scores which are calculated by combining the probabilities from different evidence channels. Moreso, protein-protein interactions are also deduced by the RiceNet database (Lee et al., 2015). This database offers gene prioritization based either on network direct neighborhood or context-associated hubs.

Apart from protein-protein interaction databases, the resources that host annotated proteomes are also crucial for proteome-wide studies. The protein sequences and their corresponding annotations are frequently updated by the UniProt database. For rice, annotations of 48916 *japonica* proteins are hosted on UniProt (Bateman et al., 2015; Consortium, 2019). Additionally, OryzaPG-DB based on the short-gun proteogenomics concept is a proteogenomics database for the annotation of rice proteome. It provides peptide-based expression profiles with corresponding genomic origin along with the annotation of novelty for each peptide (Helmy et al., 2012). Manually Curated Database of Rice Proteins (MCDRP) digitizes protein-related experiments. The process of digitization has overcome the limitations associated with text-based curation. MCDRP is periodically updated and currently contains data for approximately 1800 rice proteins (Gour et al., 2014). To study protein functions based on their structures, the plant protein annotation suit database (Plant-PrAS) is used. Various physio-chemical parameters, structural properties, novel functional regions, transmembrane helices, and signal peptides from the genomes of six model plants (including rice) are provided by Plant-PrAS (Kurotani et al., 2015).

Orthologous proteins provide valuable information about unannotated proteins. GreenPhyl DB v5 is a web-based tool for functional and comparative genomics for 27 reference genomes (including rice). It facilitates comparative analysis of species and protein domains. Metabolic pathway-related information can also be accessed via GreenPhyl DB. 44786 out of 60647 rice sequences present on GreenPhyl DB have an InterPro domain (Rouard et al., 2011). Another database that enables cross-species proteomic comparative analysis is the Putative Orthologous Groups 2 Database. This database supports three other species (*Arabidopsis thaliana, Zea mays*, and *Populus trichocarpa*) along with rice to integrate the data from predicted proteomes into putative orthologous groups. Interpro domain keyword or ID, gene model or transcript accessions, known or predicted intracellular location can be used to query the database. It provides information on probable protein localization, gene descriptions, and domain organizations (Tomcal et al., 2013). Similarly, the InParanoid database assesses the orthologs based on the InParanoid algorithm. For a specific protein, the orthologs can be searched by gene identifier, protein identifier, or by a blast search against InParanoid protein dataset (Sonnhammer and Östlund, 2015). Likewise, orthologous matrix (OMA) is a database to infer orthologs among complete genomes (Altenhoff et al., 2018). The PANTHER (Protein Analysis Through Evolutionary Relationships) tool allows the classification of proteins (and their corresponding genes) to facilitate high-throughput analysis. This tool classifies proteins according to family/sub-family, molecular function, biological process, or pathway (Thomas et al., 2003; Mi et al., 2012). Finally, PANTHER uses the library of trees to predict the orthologs. Moreover, an online orthology analysis and annotation visualization tool- plant orthology browser (POB) allows interactive pairwise comparison and visualization of genomic traits via gene orthology. It currently hosts 20 genomes, and syntenic blocks are recognized for a pair of genomes using strand orientation and physical mapping (Tulpan and Leger, 2017).

**FIGURE 2:**
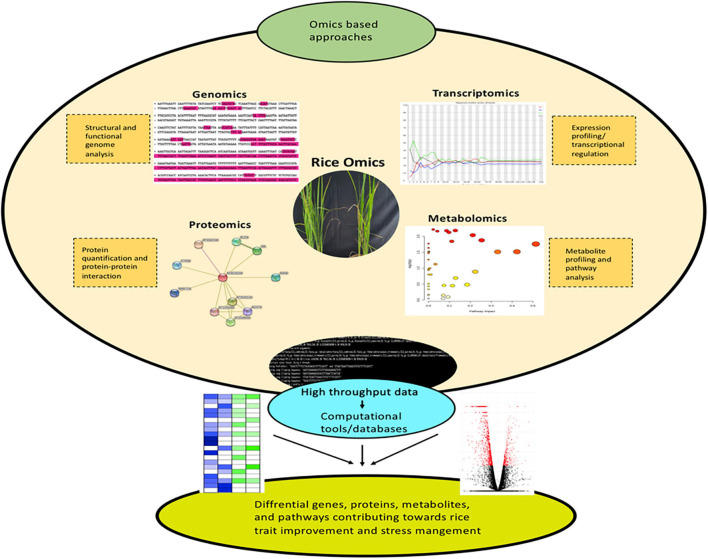
Overview of omics techniques with respective databases and tools in rice trait improvement and stress management. Databases and tools used include: genomics-PlantCARE, transcriptomics-FASTQC, proteomics-STRING, metabolomics-MetaboAnalyst 5.0. For volcano plot R was used, while MAPMAN was used to generate the heatmap.

Plants produce numerous metabolic compounds to sustain growth under normal or adverse conditions. In this direction, databases that support rice metabolome studies accelerate functional genomics research. The online platform MetaboLights hosts curated metabolite information. It offers a single access point for a number of metabolomic studies. This is a cross-species, cross-technique analysis which covers metabolite structures and their reference spectra (Haug et al., 2013). For cross-species comparative analysis, the plant metabolic network (PMN) database contains data from 22 species. It contains information related to genes, enzymes, compounds, reactions, and pathways involved in primary and secondary metabolism in plants (Schläpfer et al., 2017). The PMN hosts one multi-species reference database-PlantCyc and 126 species/taxon-specific databases. The rice metabolic database of PMN is called OryzaCyc (V 6.0). The OryzaCyc (V 6.0) houses 569 pathways consisting of 3345 reactions and 2614 compounds for 6325 enzymes. In a similar vein, RiceCyc is a catalog of known and/or predicted biochemical pathways from rice. It is developed, maintained, and curated by the Gramene database (Jaiswal et al., 2006). Gramene is an integrated data resource for comparative functional genomics which hosts 93 reference genomes, including rice (Naithani et al., 2016; Tello-Ruiz et al., 2021). Gramene provides information on metabolic networks, transport, genetic, signaling, and developmental pathways. Recently, Plant Reactome which is a comparative plant pathway knowledgebase of the Gramene project has been updated (Naithani et al., 2020; Tello-Ruiz et al., 2021). It utilizes rice as a reference plant for manual curation of pathways and currently hosts 298 reference pathways, including metabolic, transcriptional, transports, hormone, and plant developmental pathways (Naithani et al., 2020). Kyoto Encyclopedia of Genes and Genomes (KEGG) is a platform for analyzing a broad range of high-throughput datasets, including metabolome data. It helps in deciphering high-level functions and utilities of the biological system. KEGG is frequently updated (last updated 2021) and four databases (pathways, genes, compounds, and enzymes) perform the major functionalities. Small molecules and metabolite-related information can be assessed from the KEGG compounds database (Kanehisa et al., 2017). Also, the KEGG mapper is used to map a set of genes, proteins, or small molecules on network databases *viz.*, KEGG pathways and KEGG modules. The four KEGG mapping tools include reconstruct, search, color, and join. Further, the MAPMAN tool is generally used for enrichment analysis or pathway mapping in rice. The tool consists of a scavenger module, the ImageAnnotator module, and the PageMan module. Processed high-throughput datasets are fetched into the MAPMAN tool for visualizing the data (in the form of a heat map) in the context of metabolic pathways. Multiple testing correction using either benjamini hochberg, benjamini yekutieli or bonferroni is performed as a part of statistical analysis by MAPMAN (Usadel et al., 2009a, b). For GO analysis, agriGO is a popular web-based platform (Tian et al., 2017). It focuses on agricultural species and currently supports 394 species and 865 datatypes. It uses analysis tools namely Singular Enrichment Analysis (SEA), Parametric Analysis of Gene set Enrichment (PAGE), BLAST4ID (Transfer IDs by BLAST), and SEACOMPARE (Cross comparison of SEA). Custom analysis tools on agriGO include custom direct acyclic graph (DAG) tree and Scatter Plot (Tian et al., 2017). Nonetheless, it is challenging to increase the GO annotations and corresponding terms in constantly accruing datasets. Thus, the gene set enrichment analysis (GSEA) method was devised to overcome the issue of low coverage of GO-annotated genes. GSEA is a computational method that establishes the biological meaning of input genes. This is performed by measuring the overlap between an input gene list and a backend gene set. A GSEA server-PlantGSEA utilizes 20290 defined gene sets from varied resources. PlantGSEA enables the GSEA for rice and three other model plants using a unique ID (usually Affymetrix probe ID or gene locus ID) as input. The output provides enrichment analysis with statistical significance and better visualization (Yi et al., 2013). The previously discussed PANTHER tool also extends its functionality for GO analysis (Mi et al., 2017). Thus, omics tools and databases supplement in-depth rice research for a better understanding of underpinning molecular mechanisms (Figure 2). A summary of relevant omics tools and databases is provided in Table 4.

**TABLE 4:** Omics tools and databases.

Database name	Omics	Description	URL
RiceGE	Genomics	It is a rice functional genomics express database	http://signal.salk.edu/cgi-bin/RiceGE
Oryza sativa genome Database	Genomics	It provides a sequence-centered genome view for *Oryza sativa (ssp. japonica)*,	http://www.plantgdb.org/OsGDB/
MSU-RGAP	Genomics	It provides sequence and annotation data for the rice genome (Nipponbare subspecies)	http://rice.uga.edu/
RAP-DB	Genomics	It facilitates the analysis of genome structure and function of rice on the basis of the annotation.	https://rapdb.dna.affrc.go.jp/
Gramene	Comparative Genomics	It is an open-source data resource for comparative functional genomics in crops and model plants	http://www.gramene.org/
Oryzabase	Genomics	It is a comprehensive rice database providing information of classical rice genetics to recent genomics	https://shigen.nig.ac.jp/rice/oryzabase/
OryGenesDB	Genomics	It displays sequence information such as the T-DNA and Ds flanking sequence tags (FSTs)	https://orygenesdb.cirad.fr/index.html
TIGR rice genome annotation DB	Genomics	It is a group of databases for searching with the BLAST programs blastn, blastx, tblastn, or tblastx.	http://blast.jcvi.org/euk-blast/index.cgi?project=osa1
MOsDB	Genomics	It is a resource for publicly available sequences of the rice	https://pgsb.helmholtz-muenchen.de/plant/rice/index.jsp
Rice mutant DB	Genomics	It contains the information of approximate 129,000 rice T-DNA insertion (enhancer trap) lines generated by an enhancer trap system.	http://rmd.ncpgr.cn/
Information Commons for Rice (IC4R)	Genomics	It is a database contributing to rice genome sequences, updating rice gene annotations and integrating multiple omics data	http://www.ic4r.org/
RiceVarMap	Genomics	It is a database for rice genomic variation and its functional annotation.	http://ricevarmap.ncpgr.cn/
RiceFrend	Genomics	It is a gene coexpression database in rice	https://ricefrend.dna.affrc.go.jp/
Rice Transcription Factor Phylogenomics DB	Genomics	It combines various data types present in public databases encircling the structural features, orthologous relationships, availability of mutants and gene expression patterns for TF families	http://ricephylogenomics-khu.org/tf/home.php
Rice transporter database	Genomics	It contains information for all putative rice transporters	https://ricephylogenomics.ucdavis.edu/transporter/genInfo.shtml
The Rice Information GateWay (RIGW)	Genomics	It provides the sequenced genomes and related information in systematic and graphical ways	https://rice.hzau.edu.cn/rice_rs3/
Rice Pan-genome Browser	Genomics	It provides sequences and gene annotations for the rice pan-genome and Gene presence-absence variations (PAVs) of rice accessions	https://cgm.sjtu.edu.cn/3kricedb/
SNP-Seek	Genomics	Provides information related to rice SNPs	https://snp-seek.irri.org/
Ricebase	Genomics	It is a rice breeding and genetics platform integrating molecular markers, pedigrees, and whole-genome data	https://www.ricebase.org/
Rice imputation server	Genomics	It utilizes genetically and geographically diverse accessions to impute rice datasets out to 5.2M SNPs using the IMPUTE2 software.	http://rice-impute.biotech.cornell.edu/
Ensemble_rice	Comparative Genomics	It is used for genome assembly, comparative genomics, and gene annotation	http://plants.ensembl.org/Oryza_sativa/Info/Index
Rice Diversity: GWAS Viewer	Genomics	Comprehensively view GWAS data	http://rs-bt-mccouch4.biotech.cornell.edu/GWAS_Viewer/plot/
PLAZA	Comparative Genomics	It is an access point for plant comparative genomics centralizing genomic data produced by different genome sequencing programs.	https://bioinformatics.psb.ugent.be/plaza/versions/plaza_v4_monocots/
PlantGDB	Comparative Genomics	It is used for sequence assemblies and annotation	http://www.plantgdb.org/
Phytozome	Comparative Genomics	It is used for accessing, visualizing and analyzing sequenced plant genomes, as well as selected genomes and datasets	https://phytozome.jgi.doe.gov/pz/portal.html
ROAD	Genomics	It is a rice oligonucleotide array database	http://ricephylogenomics-khu.org/road/home.php
FIT-DB	Transcriptomics	It provides result of statistical modeling of transcriptomic dynamics in the field condition	https://fitdb.dna.affrc.go.jp/
EXPath 2.0	Transcriptomics	It is a database that collects and uses expression profiles derived from microarray under various conditions to infer metabolic pathways	http://expath.itps.ncku.edu.tw/
Rice eFP	Transcriptomics	One of the major functions of Rice eFP is gene expression analysis	http://bar.utoronto.ca/efprice/cgi-bin/efpWeb.cgi
RiceXPro	Transcriptomics	It is a repository of gene expression profiles derived from microarray analysis of tissues/organs	https://ricexpro.dna.affrc.go.jp/
RED	Transcriptomics	It is a repository of gene expression profiles derived from RNA-Seq data on rice tissues	http://expression.ic4r.org/
TENOR	Transcriptomics	It is the transcriptome encyclopedia of rice	https://tenor.dna.affrc.go.jp/
OryzaExpress	Transcriptomics	It is a sub-platform of PlantExpress for a single-species GEN analysis in rice.	http://plantomics.mind.meiji.ac.jp/OryzaExpress/
UniVIO	Transcriptomics	It provides a data set of hormonome and transcriptome analyses in 14 organs of rice at the reproductive stage and in gibberellin-related mutants.	http://univio.psc.riken.jp/
Genevestigator	Transcriptomics	It helps in analysis of deeply curated bulk tissue and single-cell transcriptomic data from public repositories with visualization tools	https://genevestigator.com/
Expression Atlas	Transcriptomics	It helps to deduce gene expression across species and biological conditions	https://www.ebi.ac.uk/gxa/experiments?organism=Oryza+sativa+Japonica+Group
PLACE	Transcriptomics	It is a database of motifs found in plant cis-acting regulatory DNA elements	https://www.dna.affrc.go.jp/PLACE/?action=newplace
PlantCARE	Transcriptomics	It is a database for promoter analysis	http://bioinformatics.psb.ugent.be/webtools/plantcare/html/
PPDB	Transcriptomics	It is a database for promoter analysis	http://ppdb.agr.gifu-u.ac.jp/ppdb/cgi-bin/index.cgi
PlantPAN_V2	Transcriptomics	It is an informative resource for detecting transcription factor binding sites	http://plantpan2.itps.ncku.edu.tw/index.html
NetMiner	Transcriptomics	An ensemble pipeline for high-quality RNA-seq-based gene co-expression network inference	https://github.com/czllab/NetMiner
PlantArrayNet	Transcriptomics	It generates co-expression information between genes based on correlation coefficients from accumulated microarray data of rice	http://bioinfo.mju.ac.kr/arraynet/
COP DB	Transcriptomics	It is a database management system for understanding plant gene function by associating between co-expressed genes and biological processes	http://webs2.kazusa.or.jp/kagiana/cop0911/
PODC	Transcriptomics	It is a database providing the information of gene networks (GENs) and knowledge-based functional annotations	http://plantomics.mind.meiji.ac.jp/podc/
Plant rDNA database	Transcriptomics	It is an online resource providing information on numbers and positions of ribosomal DNA signals and their structures	https://www.plantrdnadatabase.com/
PlantcircBase	Transcriptomics	It is a repository of publicly available back-splice junction sequences and their full-length sequences of circRNAs	http://ibi.zju.edu.cn/plantcircbase/
PceRBase	Transcriptomics	It is a plant competing endogenous database	http://bis.zju.edu.cn/pcernadb/index.jsp
miRBase	Transcriptomics	the microRNA database	http://www.mirbase.org/
IsomiR Bank	Transcriptomics	Database for microRNA isoforms. It is an integrative resource that contains the sequence and expression of isomers	https://mcg.ustc.edu.cn/bsc/isomir/
PNRD	Transcriptomics	It is a plant non-coding RNA database	http://structuralbiology.cau.edu.cn/PNRD/index.php
CSRDB	Transcriptomics	It is a cereal small RNAs database	http://sundarlab.ucdavis.edu/smrnas/
PlantRNA	Transcriptomics	The PlantRNA database contains tRNA gene sequences retrieved from fully annotated plant nuclear, plastidial and mitochondrial genomes.	http://plantrna.ibmp.cnrs.fr/plantrna/
Rice Interactions Viewer	Proteomics	It is a database for protein-protein rice interaction	http://bar.utoronto.ca/interactions/cgi-bin/rice_interactions_viewer.cgi
STRING	Proteomics	It is used to study protein-protein interaction and functional enrichment analysis	https://string-db.org/
PRIN	Proteomics	It is used to predict rice interactome network	http://bis.zju.edu.cn/prin/
RiceNet	Proteomics	It is an improved network prioritization web server for rice	https://www.inetbio.org/ricenet/
MCDRP	Proteomics	It is a manually curated database for rice proteins	http://www.genomeindia.org/biocuration/whatsnewver6.php
OryzaPG-DB	Proteomics	It is a rice proteogenomics database	https://github.com/MoHelmy/oryza-PG/
Plant-PrAS	Proteomics	Database for physicochemical and structural properties	http://plant-pras.riken.jp/
OMA browser	Proteomics	It is used for ortholog analysis	https://omabrowser.org/oma/home/
POGsDB	Proteomics	It facilitates cross-species inferences about gene functions	http://pogs.uoregon.edu/#/
InParanoid8	Proteomics	To study ortholog groups with inparalogs	https://inparanoid.sbc.su.se/cgi-bin/index.cgi
GreenPhyl v4	Proteomics	It is a database for species, genomes and pan-genomes	https://www.greenphyl.org/cgi-bin/index.cgi
Panther	Proteomics	It is used to perform Protein ANalysis THrough Evolutionary Relationships	http://www.pantherdb.org/
PhosphoRice	Proteomics	It is a meta-predictor of rice-specific phosphorylation site	https://github.com/PEHGP/PhosphoRice
MassBank	Metabolomics	Metabolite annotation and MS-MS database	https://massbank.eu/MassBank/
METLIN	Metabolomics	It is a collection of MS/MS data and incorporates the NIST MS/MS library and search software	https://www.sisweb.com/software/ms/wiley-metlin.htm
KNApSAcK	Metabolomics	Comprehensive Species Metabolite Relationship Database	http://www.knapsackfamily.com/KNApSAcK/
MetaboLights	Metabolomics	It is a database for metabolomics experiments and derived information	https://www.ebi.ac.uk/metabolights/
Plant Reactome	Metabolomics	Used to find reactions and pathways	https://plantreactome.gramene.org/index.php?lang=en
OryzaCYC	Metabolomics	To study plant metabolic network	https://plantcyc.org/databases/oryzacyc/6.0
KEGG_rice	Metabolomics	To decipher pathway maps	https://www.genome.jp/kegg-bin/show_organism?menu_type=pathway_maps&org=dosa
RICECYC	Metabolomics	It is a catalog of known and/or predicted biochemical pathways from rice	http://pathway.gramene.org/gramene/ricecyc.shtml
MapMan	Metabolomics	It is used in pathway analysis	https://mapman.gabipd.org/home
Plant GSEA	Metabolomics	It is used for gene set enrichment analysis	http://structuralbiology.cau.edu.cn/PlantGSEA/index.php
AgriGo	Metabolomics	It is an ontology analysis tool	http://bioinfo.cau.edu.cn/agriGO/
KEGG Mapper	Metabolomics	It is a tool for KEGG pathway mapping	https://www.genome.jp/kegg/mapper.html
MetaboAnalyst 5.0	Metabolomics	It is a comprehensive platform dedicated for metabolomics data analysis	https://www.metaboanalyst.ca/

## Conclusion and Future Prospects

Improving rice productivity mainly depends upon functional characterization and analyses of genes that are vital to agronomic traits. In rice research, high-throughput technologies had been employed for several years to gain insights into the mechanistic details of molecular pathways. Genomics provides information regarding the most dominant or recessive genes in rice varieties, while transcriptomics aids in elucidating complex expression networks of RNA in rice that can be imperative to yield or stress responses. Similarly, proteomics leads to ascertaining major proteins contributing to rice improvement, while metabolomics provides crucial signatures of metabolites related to rice quality and yield enrichment. Bioinformatics databases assimilate the data from omics sciences to generate the complete set of information about the factors contributing to the enhancement of quality, quantity, or stress responses in rice. Thus, the omics generated datasets can expedite gene discoveries and functional characterizations in rice for crop improvement. Also, plant system biology has deepened the understanding of metabolism, stress responses, and integrative omics research. Moreover, the advent of CRISPR/Cas9 genome editing technology and its combination with omics studies has widened the horizons of rice research. Rice omics research is the new avenue that offers great potential. An integrative omics platform offering access to complete bioinformatics data will help researchers to implement new techniques in forward/reverse genetics and breeding programs. Taken together, omics-based rice research along with the cutting-edge technologies holds great potential for rice yield enhancement and stress management.

## Author Contributions

MIA conceptualized and designed the study. ZI, MSI, and MIRK compiled the data and wrote the manuscript. All authors have read the manuscript and agreed for publication.

## Conflict of Interest

The authors declare that the research was conducted in the absence of any commercial or financial relationships that could be construed as a potential conflict of interest.

## Publisher’s Note

All claims expressed in this article are solely those of the authors and do not necessarily represent those of their affiliated organizations, or those of the publisher, the editors and the reviewers. Any product that may be evaluated in this article, or claim that may be made by its manufacturer, is not guaranteed or endorsed by the publisher.

## Footnotes

1
http://www.fao.org/

2
http://rapdb.dna.affrc.go.jp/

## References

[B1] Agrawal G. K. Rakwal R. (2011). Rice proteomics: a move toward expanded proteome coverage to comparative and functional proteomics uncovers the mysteries of rice and plant biology. *Proteomics* 11 1630–1649. 10.1002/pmic.201000696 21462347

[B2] Agrawal G. K. Jwa N. S. Lebrun M. H. Job D. Rakwal R. (2010). Plant secretome: unlocking secrets of the secreted proteins. *Proteomics* 10 799–827. 19953550 10.1002/pmic.200900514

[B3] Agrawal G. K. Sarkar A. Righetti P. G. Pedreschi R. Carpentier S. Wang T. (2013). A decade of plant proteomics and mass spectrometry: translation of technical advancements to food security and safety issues. *Mass Spectromet. Rev.* 32 335–365. 10.1002/mas.21365 23315723

[B4] Ahuja I. Kissen R. Bones A. M. (2012). Phytoalexins in defense against pathogens. *Trends Plant Sci.* 17 73–90. 22209038 10.1016/j.tplants.2011.11.002

[B5] Alam R. Sazzadur Rahman M. Seraj Z. I. Thomson M. J. Ismail A. M. Tumimbang-Raiz E. (2011). Investigation of seedling-stage salinity tolerance QTLs using backcross lines derived from Oryza sativa L. Pokkali. *Plant Breed.* 130 430–437.

[B6] Alexandrov N. Tai S. Wang W. Mansueto L. Palis K. Fuentes R. R. (2015). SNP-Seek database of SNPs derived from 3000 rice genomes. *Nucleic Acids Res.* 43 D1023–D1027. 10.1093/nar/gku1039 25429973 PMC4383887

[B7] Altenhoff A. M. Glover N. M. Train C.-M. Kaleb K. Warwick Vesztrocy A. Dylus D. (2018). The OMA orthology database in 2018: retrieving evolutionary relationships among all domains of life through richer web and programmatic interfaces. *Nucleic Acids Res.* 46 D477–D485. 10.1093/nar/gkx1019 29106550 PMC5753216

[B8] Amoah N. K. A. Akromah R. Kena A. W. Manneh B. Dieng I. Bimpong I. K. (2020). Mapping QTLs for tolerance to salt stress at the early seedling stage in rice (Oryza sativa L.) using a newly identified donor ‘Madina Koyo’. *Euphytica* 216 1–23.

[B9] Anacleto R. Badoni S. Parween S. Butardo V. M. Jr. Misra G. Cuevas R. P. (2019). Integrating a genome-wide association study with a large-scale transcriptome analysis to predict genetic regions influencing the glycaemic index and texture in rice. *Plant Biotechnol. J.* 17 1261–1275. 10.1111/pbi.13051 30549178 PMC6575982

[B10] Ansari M. I. Jalil S. U. Ansari S. A. Hasanuzzaman M. (2021). GABA shunt: a key-player in mitigation of ROS during stress. *Plant Growth Regulat.* 2021 1–19.

[B11] Ansari M. I. Lee R. H. Chen S. C. G. (2005). A novel senescence-associated gene encoding γ-aminobutyric acid (GABA): pyruvate transaminase is upregulated during rice leaf senescence. *Physiol. Plant.* 123 1–8.

[B12] Ashikari M. Lin S. Yamamoto T. Takashi T. Nishimura A. Angeles E. (2007). “Isolation of a QTL gene controlling grain number and QTL pyramiding to combine loci for grain number and plant height in rice,” in *Rice Genetics V*. *World Sci.* 2007 169–175.

[B13] Ashikari M. Sakakibara H. Lin S. Yamamoto T. Takashi T. Nishimura A. (2005). Cytokinin oxidase regulates rice grain production. *Science* 309 741–745. 15976269 10.1126/science.1113373

[B14] Azizi P. Osman M. Hanafi M. M. Sahebi M. Yusop M. R. Taheri S. (2019). Adaptation of the metabolomics profile of rice after Pyricularia oryzae infection. *Plant Physiol. Biochem.* 144 466–479. 10.1016/j.plaphy.2019.10.014 31655345

[B15] Bailey T. L. Boden M. Buske F. A. Frith M. Grant C. E. Clementi L. (2009). MEME SUITE: tools for motif discovery and searching. *Nucleic Acids Res.* 37 W202–W208. 19458158 10.1093/nar/gkp335 PMC2703892

[B16] Baldrich P. San Segundo B. (2016). MicroRNAs in rice innate immunity. *Rice* 9 1–9. 26897721 10.1186/s12284-016-0078-5 PMC4761359

[B17] Bao J. (2014). *Genes and QTLs for rice grain quality improvement.* London: IntechOpen, 239–278.

[B18] Bao J. Lee S. Chen C. Zhang X. Zhang Y. Liu S. (2005). Serial analysis of gene expression study of a hybrid rice strain (LYP9) and its parental cultivars. *Plant Physiol.* 138 1216–1231. 10.1104/pp.105.060988 16009997 PMC1176396

[B19] Baranski M. (2013). *Golden rice.* Arizona, AZ: Embryo Project Encyclopedia.

[B20] Barrera-Figueroa B. E. Gao L. Wu Z. Zhou X. Zhu J. Jin H. (2012). High throughput sequencing reveals novel and abiotic stress-regulated microRNAs in the inflorescences of rice. *BMC Plant Biol.* 12:1–11. 10.1186/1471-2229-12-132 22862743 PMC3431262

[B21] Baslam M. Mitsui T. (2020). “Proteomic for Quality: Mining the Proteome as a Strategy to Elucidate the Protein Complex Applied for Quality Improvement,” in *The Future of Rice Demand: Quality Beyond Productivity*, eds de Oliveira A. C. Pegoraro C. Viana V. E. (Berlin: Springer), 473–494.

[B22] Bateman A. Martin M. O’donovan C. Magrane M. Apweiler R. Alpi E. (2015). 440 Bingley M, et al. *UniProt: A hub for protein information*. *Nucleic Acids Res.* 43 D204–D441. 10.1093/nar/gku989 25348405 PMC4384041

[B23] Bekele W. A. Wieckhorst S. Friedt W. Snowdon R. J. (2013). High-throughput genomics in sorghum: from whole-genome resequencing to a SNP screening array. *Plant Biotechnol. J.* 11 1112–1125. 10.1111/pbi.12106 23919585

[B24] Bernier J. Kumar A. Ramaiah V. Spaner D. Atlin G. (2007). A large-effect QTL for grain yield under reproductive-stage drought stress in upland rice. *Crop Sci.* 47 507–516.

[B25] Bernier J. Serraj R. Kumar A. Venuprasad R. Impa S. Rp V. G. (2009). The large-effect drought-resistance QTL qtl12. 1 increases water uptake in upland rice. *Field Crops Res.* 110 139–146.

[B26] Bimpong I. K. Manneh B. Diop B. Ghislain K. Sow A. Amoah N. K. A. (2014a). New quantitative trait loci for enhancing adaptation to salinity in rice from Hasawi, a Saudi landrace into three African cultivars at the reproductive stage. *Euphytica* 200 45–60.

[B27] Bimpong I. K. Manneh B. El-Namaky R. Diaw F. Amoah N. K. A. Sanneh B. (2014b). Mapping QTLs related to salt tolerance in rice at the young seedling stage using 384-plex single nucleotide polymorphism SNP, marker sets. *Mol. Plant Breed.* 5:009.

[B28] Bin Rahman A. R. Zhang J. (2016). Flood and drought tolerance in rice: opposite but may coexist. *Food Energy Secur.* 5 76–88.

[B29] Bizimana J. B. Luzi-Kihupi A. Murori R. W. Singh R. (2017). Identification of quantitative trait loci for salinity tolerance in rice (Oryza sativa L.) using IR29/Hasawi mapping population. *J. Genet.* 96 571–582. 10.1007/s12041-017-0803-x 28947705

[B30] Blumenberg M. (ed.) (2019). “Introductory chapter: Transcriptome analysis,” in *Transcriptome Analysis*, (London: IntechOpen).

[B31] Bonilla P. Dvorak J. Mackell D. Deal K. Gregorio G. (2002). RFLP and SSLP mapping of salinity tolerance genes in chromosome 1 of rice (Oryza sativa L.) using recombinant inbred lines. *Philipp. Agricult. Sci.* 65 68–76.

[B32] Bradbury L. M. Gillies S. A. Brushett D. J. Waters D. L. Henry R. J. (2008). Inactivation of an aminoaldehyde dehydrogenase is responsible for fragrance in rice. *Plant Mol. Biol.* 68 439–449. 10.1007/s11103-008-9381-x 18704694

[B33] Calingacion M. Boualaphanh C. Daygon V. Anacleto R. Hamilton R. S. Biais B. (2012). A genomics and multi-platform metabolomics approach to identify new traits of rice quality in traditional and improved varieties. *Metabolomics* 8 771–783.

[B34] Campo S. Peris-Peris C. Siré C. Moreno A. B. Donaire L. Zytnicki M. (2013). Identification of a novel micro RNA (mi RNA) from rice that targets an alternatively spliced transcript of the N ramp6 (N atural resistance-associated macrophage protein 6) gene involved in pathogen resistance. *N. Phytol.* 199 212–227. 10.1111/nph.12292 23627500

[B35] Cao P. Jung K.-H. Choi D. Hwang D. Zhu J. Ronald P. C. (2012). The Rice Oligonucleotide Array Database: an atlas of rice gene expression. *Rice* 5 1–9. 10.1186/1939-8433-5-17 24279809 PMC4883718

[B36] Catolos M. Sandhu N. Dixit S. Shamsudin N. A. Naredo M. E. Mcnally K. L. (2017). Genetic loci governing grain yield and root development under variable rice cultivation conditions. *Front. Plant Sci.* 8:1763. 10.3389/fpls.2017.01763 29085383 PMC5650699

[B37] Chakraborty K. Guru A. Jena P. Ray S. Guhey A. Chattopadhyay K. (2021). Rice with SUB1 QTL possesses greater initial leaf gas film thickness leading to delayed perception of submergence stress. *Ann. Bot.* 127 251–265. 10.1093/aob/mcaa171 32939540 PMC7789114

[B38] Chamam A. Sanguin H. Bellvert F. Meiffren G. Comte G. Wisniewski-Dyé F. (2013). Plant secondary metabolite profiling evidences strain-dependent effect in the Azospirillum–Oryza sativa association. *Phytochemistry* 87 65–77. 10.1016/j.phytochem.2012.11.009 23266268

[B39] Chang Q.-L. Xu H.-J. Peng Y.-L. Fan J. (2019). Subtractive hybridization-assisted screening and characterization of genes involved in the rice-Magnaporthe oryzae interaction. *Phytopathol. Res.* 1 1–13.

[B40] Chen C. J. Zhang Z. (2018). iPat: intelligent prediction and association tool for genomic research. *Bioinformatics* 34 1925–1927. 10.1093/bioinformatics/bty015 29342241

[B41] Chen C.-J. Liu Q. Zhang Y.-C. Qu L.-H. Chen Y.-Q. Gautheret D. (2011). Genome-wide discovery and analysis of microRNAs and other small RNAs from rice embryogenic callus. *RNA Biol.* 8 538–547. 10.4161/rna.8.3.15199 21525786

[B42] Chen R. Zhao X. Shao Z. Wei Z. Wang Y. Zhu L. (2007). Rice UDP-glucose pyrophosphorylase1 is essential for pollen callose deposition and its cosuppression results in a new type of thermosensitive genic male sterility. *Plant Cell* 19 847–861. 10.1105/tpc.106.044123 17400897 PMC1867369

[B43] Chen W. Gao Y. Xie W. Gong L. Lu K. Wang W. (2014). Genome-wide association analyses provide genetic and biochemical insights into natural variation in rice metabolism. *Nat. Genet.* 46 714–721. 10.1038/ng.3007 24908251

[B44] Chen X. Deng Z. Yu C. Yan C. Chen J. (2016). Secretome analysis of rice suspension-cultured cells infected by Xanthomonas oryzae pv. oryza (Xoo). *Proteome Sci.* 14 1–10. 26839515 10.1186/s12953-016-0091-z PMC4735954

[B45] Chen X. Tao Y. Ali A. Zhuang Z. Guo D. Guo Q. (2019). Transcriptome and proteome profiling of different colored rice reveals physiological dynamics involved in the flavonoid pathway. *Int. J. Mol. Sci.* 20:2463. 10.3390/ijms20102463 31109052 PMC6566916

[B46] Chi F. Yang P. Han F. Jing Y. Shen S. (2010). Proteomic analysis of rice seedlings infected by Sinorhizobium meliloti 1021. *Proteomics* 10 1861–1874. 10.1002/pmic.200900694 20213677

[B47] Chien C.-H. Chow C.-N. Wu N.-Y. Chiang-Hsieh Y.-F. Hou P.-F. Chang W.-C. (2015). EXPath: a database of comparative expression analysis inferring metabolic pathways for plants. *BMC Genom.* 16(Suppl. 2):S6. 10.1186/1471-2164-16-S2-S6 25708775 PMC4331720

[B48] Chintakovid N. Maipoka M. Phaonakrop N. Mickelbart M. V. Roytrakul S. Chadchawan S. (2017). Proteomic analysis of drought-responsive proteins in rice reveals photosynthesis-related adaptations to drought stress. *Acta Physiol. Plant.* 39 1–13.

[B49] Cho W. K. Kim J.-Y. (2009). Integrated analyses of the rice secretome. *Plant Signal. Behav.* 4 345–347. 19794859 10.4161/psb.4.4.8198 PMC2664503

[B50] Choudhary M. K. Basu D. Datta A. Chakraborty N. Chakraborty S. (2009). Dehydration-responsive nuclear proteome of rice (Oryza sativa L.) illustrates protein network, novel regulators of cellular adaptation, and evolutionary perspective. *Mol. Cell. Proteom.* 8 1579–1598. 10.1074/mcp.M800601-MCP200 19321431 PMC2709188

[B51] Chow C.-N. Zheng H.-Q. Wu N.-Y. Chien C.-H. Huang H.-D. Lee T.-Y. (2016). PlantPAN 2.0: an update of plant promoter analysis navigator for reconstructing transcriptional regulatory networks in plants. *Nucleic Acids Res.* 44 D1154–D1160. 10.1093/nar/gkv1035 26476450 PMC4702776

[B52] Chu Q. Zhang X. Zhu X. Liu C. Mao L. Ye C. (2017). PlantcircBase: a database for plant circular RNAs. *Mol. Plant* 10 1126–1128. 28315753 10.1016/j.molp.2017.03.003

[B53] Consortium U. (2019). UniProt: a worldwide hub of protein knowledge. *Nucleic Acids Res.* 47 D506–D515. 10.1093/nar/gky1049 30395287 PMC6323992

[B54] Das G. Patra J. K. Baek K.-H. (2017). Insight into MAS: a molecular tool for development of stress resistant and quality of rice through gene stacking. *Front. Plant Sci.* 8:985. 10.3389/fpls.2017.00985 28659941 PMC5469070

[B55] Dash P. K. Rai R. Rai V. Pasupalak S. (2018). Drought induced signaling in rice: delineating canonical and non-canonical pathways. *Front. Chem.* 6:264. 10.3389/fchem.2018.00264 30258837 PMC6143666

[B56] Dash S. Van Hemert J. Hong L. Wise R. P. Dickerson J. A. (2012). PLEXdb: gene expression resources for plants and plant pathogens. *Nucleic Acids Res.* 40 D1194–D1201. 22084198 10.1093/nar/gkr938 PMC3245067

[B57] Daviss B. (2005). Growing pains for metabolomics: the newest’omic science is producing results–and more data than researchers know what to do with. *Scientist* 19 25–29.

[B58] Daygon V. D. Calingacion M. Forster L. C. De Voss J. J. Schwartz B. D. Ovenden B. (2017). Metabolomics and genomics combine to unravel the pathway for the presence of fragrance in rice. *Sci. Rep.* 7 1–12. 10.1038/s41598-017-07693-9 28821745 PMC5562744

[B59] Decourcelle M. Perez-Fons L. Baulande S. Steiger S. Couvelard L. Hem S. (2015). Combined transcript, proteome, and metabolite analysis of transgenic maize seeds engineered for enhanced carotenoid synthesis reveals pleotropic effects in core metabolism. *J. Exp. Bot.* 66 3141–3150. 10.1093/jxb/erv120 25796085 PMC4449536

[B60] Delseny M. Salses J. Cooke R. Sallaud C. Regad F. Lagoda P. (2001). Rice genomics: present and future. *Plant Physiol. Biochem.* 39 323–334.

[B61] Deng C. Ye H. Fan M. Pu T. Yan J. (2017). The rice transcription factors OsICE confer enhanced cold tolerance in transgenic Arabidopsis. *Plant Signal. Behav.* 12:e1316442. 10.1080/15592324.2017.1316442 28414264 PMC5501220

[B62] Deng P. Jiang D. Dong Y. Shi X. Jing W. Zhang W. (2015a). Physiological characterisation and fine mapping of a salt-tolerant mutant in rice (Oryza sativa). *Funct. Plant Biol.* 42 1026–1035. 10.1071/FP15126 32480742

[B63] Deng P. Shi X. Zhou J. Wang F. Dong Y. Jing W. (2015b). Identification and Fine Mapping of a Mutation Conferring Salt-Sensitivity in Rice (Oryza sativa L.). *Crop Sci.* 55 219–228.

[B64] Devi S. R. Singh K. Umakanth B. Vishalakshi B. Rao K. V. S. Suneel B. (2020). Identification and characterization of a large effect QTL from Oryza glumaepatula revealed Pi68 (t) as putative candidate gene for rice blast resistance. *Rice* 13 1–13. 10.1186/s12284-020-00378-4 32166467 PMC7067966

[B65] Dhakarey R. Raorane M. L. Treumann A. Peethambaran P. K. Schendel R. R. Sahi V. P. (2017). Physiological and proteomic analysis of the rice mutant cpm2 suggests a negative regulatory role of jasmonic acid in drought tolerance. *Front. Plant Sci.* 8:1903. 10.3389/fpls.2017.01903 29250082 PMC5715382

[B66] Dhondt S. Wuyts N. Inzé D. (2013). Cell to whole-plant phenotyping: the best is yet to come. *Trends Plant Sci.* 18 428–439. 10.1016/j.tplants.2013.04.008 23706697

[B67] Ding Y. Chen Z. Zhu C. (2011). Microarray-based analysis of cadmium-responsive microRNAs in rice (Oryza sativa). *J. Exp. Bot.* 62 3563–3573. 10.1093/jxb/err046 21362738 PMC3130178

[B68] Divya D. Sahu N. Reddy P. S. Nair S. Bentur J. (2021). RNA-Sequencing Reveals Differentially Expressed Rice Genes Functionally Associated with Defense against BPH and WBPH in RILs Derived from a Cross between RP2068 and TN1. *Rice* 14 1–21. 10.1186/s12284-021-00470-3 33677774 PMC7936997

[B69] Dong C. He F. Berkowitz O. Liu J. Cao P. Tang M. (2018). Alternative splicing plays a critical role in maintaining mineral nutrient homeostasis in rice (Oryza sativa). *Plant Cell* 30 2267–2285. 10.1105/tpc.18.00051 30254029 PMC6241280

[B70] Dong M. Gu J. Zhang L. Chen P. Liu T. Deng J. (2014). Comparative proteomics analysis of superior and inferior spikelets in hybrid rice during grain filling and response of inferior spikelets to drought stress using isobaric tags for relative and absolute quantification. *J. Proteom.* 109 382–399. 10.1016/j.jprot.2014.07.001 25058577

[B71] Droc G. Ruiz M. Larmande P. Pereira A. Piffanelli P. Morel J. B. (2006). OryGenesDB: a database for rice reverse genetics. *Nucleic Acids Res.* 34 D736–D740. 16381969 10.1093/nar/gkj012 PMC1347375

[B72] Du H. Huang F. Wu N. Li X. Hu H. Xiong L. (2018). Integrative regulation of drought escape through ABA-dependent and-independent pathways in rice. *Mol. Plant* 11 584–597. 10.1016/j.molp.2018.01.004 29366830

[B73] Du J. Shen T. Xiong Q. Zhu C. Peng X. He X. (2020). Combined proteomics, metabolomics and physiological analyses of rice growth and grain yield with heavy nitrogen application before and after drought. *BMC Plant Biol.* 20:1–17. 10.1186/s12870-020-02772-y 33302870 PMC7731554

[B74] Du S. Cui M. Cai Y. Xue A. Hao Y. Huang X. (2020). Metabolomic analysis of chilling response in rice (Oryza sativa L.) seedlings by extractive electrospray ionization mass spectrometry. *Environ. Exp. Bot.* 180:104231.

[B75] Edwards J. D. Baldo A. Mueller L. A. (2016). Ricebase: a breeding and genetics platform for rice, integrating individual molecular markers, pedigrees and whole-genome-based data. *Database* 2016:baw107. 10.1093/database/baw107 27515824 PMC4980570

[B76] Emon R. M. Islam M. M. Halder J. Fan Y. (2015). Genetic diversity and association mapping for salinity tolerance in Bangladeshi rice landraces. *Crop J.* 3 440–444.

[B77] Ereful N. C. Liu L.-Y. Greenland A. Powell W. Mackay I. Leung H. (2020). RNA-seq reveals differentially expressed genes between two indica inbred rice genotypes associated with drought-yield QTLs. *Agronomy* 10:621.

[B78] Fan C. Xing Y. Mao H. Lu T. Han B. Xu C. (2006). GS3, a major QTL for grain length and weight and minor QTL for grain width and thickness in rice, encodes a putative transmembrane protein. *Theoret. Appl. Genet.* 112 1164–1171. 10.1007/s00122-006-0218-1 16453132

[B79] Feltus F. A. Wan J. Schulze S. R. Estill J. C. Jiang N. Paterson A. H. (2004). An SNP resource for rice genetics and breeding based on subspecies indica and japonica genome alignments. *Genome Res.* 14 1812–1819. 10.1101/gr.2479404 15342564 PMC515328

[B80] Fu J. Wu H. Ma S. Xiang D. Liu R. Xiong L. (2017). OsJAZ1 attenuates drought resistance by regulating JA and ABA signaling in rice. *Front. Plant Sci.* 8:2108. 10.3389/fpls.2017.02108 29312378 PMC5733117

[B81] Fukao T. Bailey-Serres J. (2008). Submergence tolerance conferred by Sub1A is mediated by SLR1 and SLRL1 restriction of gibberellin responses in rice. *PNAS* 105 16814–16819. 10.1073/pnas.0807821105 18936491 PMC2575502

[B82] Fukushima A. Kusano M. (2014). A network perspective on nitrogen metabolism from model to crop plants using integrated ‘omics’ approaches. *J. Exp. Bot.* 65 5619–5630. 25129130 10.1093/jxb/eru322

[B83] Gao P. Bai X. Yang L. Lv D. Pan X. Li Y. (2011). osa-MIR393: a salinity-and alkaline stress-related microRNA gene. *Mol. Biol. Rep.* 38 237–242. 10.1007/s11033-010-0100-8 20336383

[B84] Gao Z.-Y. Zhao S.-C. He W.-M. Guo L.-B. Peng Y.-L. Wang J.-J. (2013). Dissecting yield-associated loci in super hybrid rice by resequencing recombinant inbred lines and improving parental genome sequences. *PNAS* 110 14492–14497. 10.1073/pnas.1306579110 23940322 PMC3761582

[B85] Gayen D. Ghosh S. Paul S. Sarkar S. N. Datta S. K. Datta K. (2016). Metabolic regulation of carotenoid-enriched golden rice line. *Front. Plant Sci.* 7:1622. 10.3389/fpls.2016.01622 27840631 PMC5083848

[B86] Ghimire K. H. Quiatchon L. A. Vikram P. Swamy B. M. Dixit S. Ahmed H. (2012). Identification and mapping of a QTL (qDTY1. 1) with a consistent effect on grain yield under drought. *Field Crops Res.* 131 88–96.

[B87] Ghosh S. Kanwar P. Jha G. (2017). Alterations in rice chloroplast integrity, photosynthesis and metabolome associated with pathogenesis of Rhizoctonia solani. *Sci. Rep.* 7 1–12. 10.1038/srep41610 28165003 PMC5292701

[B88] Goff S. A. Ricke D. Lan T.-H. Presting G. Wang R. Dunn M. (2002). A draft sequence of the rice genome (Oryza sativa L. ssp. japonica). *Science* 296 92–100. 11935018 10.1126/science.1068275

[B89] Gong L. Chen W. Gao Y. Liu X. Zhang H. Xu C. (2013). Genetic analysis of the metabolome exemplified using a rice population. *PNAS* 110 20320–20325. 10.1073/pnas.1319681110 24259710 PMC3864304

[B90] Gour P. Garg P. Jain R. Joseph S. V. Tyagi A. K. Raghuvanshi S. (2014). Manually curated database of rice proteins. *Nucleic Acids Res.* 42 D1214–D1221. 24214963 10.1093/nar/gkt1072 PMC3964970

[B91] Graziano S. Marmiroli N. Gullì M. (2020). Proteomic analysis of reserve proteins in commercial rice cultivars. *Food Sci. Nutrit.* 8 1788–1797. 10.1002/fsn3.1375 32328244 PMC7174207

[B92] Gregorio G. B. (1997). *Tagging salinity tolerance genes in rice using amplified fragment length polymorphism (AFLP).* Ph. D. thesis. Los Baños: University of the Philippines.

[B93] Groen S. C. Ćalić I. Joly-Lopez Z. Platts A. E. Choi J. Y. Natividad M. (2020). The strength and pattern of natural selection on gene expression in rice. *Nature* 578 572–576. 32051590 10.1038/s41586-020-1997-2

[B94] Gu H. Zhu P. Jiao Y. Meng Y. Chen M. (2011). PRIN: a predicted rice interactome network. *BMC Bioinformatics* 12:1–13. 10.1186/1471-2105-12-161 21575196 PMC3118165

[B95] Gumi A. M. Guha P. K. Mazumder A. Jayaswal P. Mondal T. K. (2018). Characterization of OglDREB2A gene from African rice (Oryza glaberrima), comparative analysis and its transcriptional regulation under salinity stress. *3 Biotech* 8 1–16. 10.1007/s13205-018-1098-1 29430353 PMC5796934

[B96] Guo H. Wu T. Li S. He Q. Yang Z. Zhang W. (2019). The methylation patterns and transcriptional responses to chilling stress at the seedling stage in rice. *Int. J. Mol. Sci.* 20:5089. 10.3390/ijms20205089 31615063 PMC6829347

[B97] Guo S. Xu Y. Liu H. Mao Z. Zhang C. Ma Y. (2013). The interaction between OsMADS57 and OsTB1 modulates rice tillering via DWARF14. *Nat. Commun.* 4:1566. 10.1038/ncomms2542 23463009 PMC3615354

[B98] Gupta A. Rico-Medina A. Caño-Delgado A. I. (2020). The physiology of plant responses to drought. *Science* 368 266–269. 32299946 10.1126/science.aaz7614

[B99] Gupta P. De B. (2017). Metabolomics analysis of rice responses to salinity stress revealed elevation of serotonin, and gentisic acid levels in leaves of tolerant varieties. *Plant Signal. Behav.* 12:e1335845. 10.1080/15592324.2017.1335845 28594277 PMC5586353

[B100] Hall R. D. (2006). Plant metabolomics: from holistic hope, to hype, to hot topic. *N. Phytol.* 169 453–468. 10.1111/j.1469-8137.2005.01632.x 16411949

[B101] Hamada K. Hongo K. Suwabe K. Shimizu A. Nagayama T. Abe R. (2011). OryzaExpress: an integrated database of gene expression networks and omics annotations in rice. *Plant Cell Physiol.* 52 220–229. 10.1093/pcp/pcq195 21186175 PMC3037078

[B102] Hamzelou S. Pascovici D. Kamath K. S. Amirkhani A. Mckay M. Mirzaei M. (2020). Proteomic responses to drought vary widely among eight diverse genotypes of rice (Oryza sativa). *Int. J. Mol. Sci.* 21:363. 10.3390/ijms21010363 31935846 PMC6982093

[B103] Han C. He D. Li M. Yang P. (2014a). In-depth proteomic analysis of rice embryo reveals its important roles in seed germination. *Plant Cell Physiol.* 55 1826–1847. 10.1093/pcp/pcu114 25231964

[B104] Han C. Yang P. Sakata K. Komatsu S. (2014b). Quantitative proteomics reveals the role of protein phosphorylation in rice embryos during early stages of germination. *J. Proteome Res.* 13 1766–1782. 10.1021/pr401295c 24460219

[B105] Haq T. U. Gorham J. Akhtar J. Akhtar N. Steele K. A. (2010). Dynamic quantitative trait loci for salt stress components on chromosome 1 of rice. *Funct. Plant Biol.* 37 634–645.

[B106] Haug K. Salek R. M. Conesa P. Hastings J. De Matos P. Rijnbeek M. (2013). MetaboLights—an open-access general-purpose repository for metabolomics studies and associated meta-data. *Nucleic Acids Res.* 41 D781–D786. 10.1093/nar/gks1004 23109552 PMC3531110

[B107] Heather J. M. Chain B. (2016). The sequence of sequencers: The history of sequencing DNA. *Genomics* 107 1–8. 26554401 10.1016/j.ygeno.2015.11.003 PMC4727787

[B108] Helmy M. Sugiyama N. Tomita M. Ishihama Y. (2012). The rice proteogenomics database OryzaPG-DB: development, expansion, and new features. *Front. Plant Sci.* 3:65. 10.3389/fpls.2012.00065 22639657 PMC3355581

[B109] Helmy M. Tomita M. Ishihama Y. (2011). OryzaPG-DB: rice proteome database based on shotgun proteogenomics. *BMC Plant Biol.* 11:63. 10.1186/1471-2229-11-63 21486466 PMC3094275

[B110] Higo K. Ugawa Y. Iwamoto M. Korenaga T. (1999). Plant cis-acting regulatory DNA elements (PLACE) database: 1999. *Nucleic Acids Res.* 27 297–300. 10.1093/nar/27.1.297 9847208 PMC148163

[B111] Ho C.-L. Wu Y. Shen H.-B. Provart N. J. Geisler M. (2012). A predicted protein interactome for rice. *Rice* 5 1–14. 24279740 10.1186/1939-8433-5-15 PMC4883691

[B112] Hrdlickova R. Toloue M. Tian B. (2017). RNA-Seq methods for transcriptome analysis. *Wiley Interdiscipl. Rev. RNA* 8:e1364. 10.1002/wrna.1364 PMC5717752 27198714

[B113] Hruz T. Laule O. Szabo G. Wessendorp F. Bleuler S. Oertle L. (2008). Genevestigator v3: a reference expression database for the meta-analysis of transcriptomes. *Adv. Bioinformat.* 2008:420747. 10.1155/2008/420747 19956698 PMC2777001

[B114] Hu C. Shi J. Quan S. Cui B. Kleessen S. Nikoloski Z. (2014). Metabolic variation between japonica and indica rice cultivars as revealed by non-targeted metabolomics. *Sci. Rep.* 4 1–10. 10.1038/srep05067 24861081 PMC5381408

[B115] Hu H. Dai M. Yao J. Xiao B. Li X. Zhang Q. (2006). Overexpressing a NAM, ATAF, and CUC (NAC) transcription factor enhances drought resistance and salt tolerance in rice. *PNAS* 103 12987–12992. 10.1073/pnas.0604882103 16924117 PMC1559740

[B116] Hu H. You J. Fang Y. Zhu X. Qi Z. Xiong L. (2008). Characterization of transcription factor gene SNAC2 conferring cold and salt tolerance in rice. *Plant Mol. Biol.* 67 169–181. 10.1007/s11103-008-9309-5 18273684

[B117] Huang J. Yang X. Wang M.-M. Tang H.-J. Ding L.-Y. Shen Y. (2007). A novel rice C2H2-type zinc finger protein lacking DLN-box/EAR-motif plays a role in salt tolerance. *Biochim. Biophys. Acta Gene Struct. Exp.* 1769 220–227. 10.1016/j.bbaexp.2007.02.006 17434609

[B118] Huang L. Wang Y. Wang W. Zhao X. Qin Q. Sun F. (2018). Characterization of transcription factor gene OsDRAP1 conferring drought tolerance in rice. *Front. Plant Sci.* 9:94. 10.3389/fpls.2018.00094 29449862 PMC5799227

[B119] Huang X. Feng Q. Qian Q. Zhao Q. Wang L. Wang A. (2009). High-throughput genotyping by whole-genome resequencing. *Genome Res.* 19 1068–1076. 19420380 10.1101/gr.089516.108 PMC2694477

[B120] Huang X. Qian Q. Liu Z. Sun H. He S. Luo D. (2009). Natural variation at the DEP1 locus enhances grain yield in rice. *Nat. Genet.* 41 494–497. 10.1038/ng.352 19305410

[B121] Huang S. Q. Peng J. Qiu C. X. Yang Z. M. (2009). Heavy metal-regulated new microRNAs from rice. *J. Inorganic Biochem.* 103 282–287. 10.1016/j.jinorgbio.2008.10.019 19081140

[B122] Huang X. Lu T. Han B. (2013). Resequencing rice genomes: an emerging new era of rice genomics. *Trends Genet.* 29 225–232. 10.1016/j.tig.2012.12.001 23295340

[B123] Iqbal Z. Iqbal M. S. Ahmad A. Memon A. G. Ansari M. I. (2020a). New prospects on the horizon: genome editing to engineer plants for desirable traits. *Curr. Plant Biol.* 2020:100171.

[B124] Iqbal Z. Shariq Iqbal M. Singh S. P. Buaboocha T. (2020b). Ca2+/calmodulin complex triggers CAMTA transcriptional machinery under stress in plants: signaling cascade and molecular regulation. *Front. Plant Sci.* 11:598327. 10.3389/fpls.2020.598327 33343600 PMC7744605

[B125] Izawa T. Shimamoto K. (1996). Becoming a model plant: the importance of rice to plant science. *Trends Plant Sci.* 1 95–99.

[B126] Jaiswal D. K. Ray D. Choudhary M. K. Subba P. Kumar A. Verma J. (2013). Comparative proteomics of dehydration response in the rice nucleus: New insights into the molecular basis of genotype-specific adaptation. *Proteomics* 13 3478–3497. 10.1002/pmic.201300284 24133045

[B127] Jaiswal P. Ni J. Yap I. Ware D. Spooner W. Youens-Clark K. (2006). Gramene: a bird’s eye view of cereal genomes’. *Nucleic Acids Res.* 34 D717–D723. 10.1093/nar/gkj154 16381966 PMC1347516

[B128] Jan A. Maruyama K. Todaka D. Kidokoro S. Abo M. Yoshimura E. (2013). OsTZF1, a CCCH-tandem zinc finger protein, confers delayed senescence and stress tolerance in rice by regulating stress-related genes. *Plant Physiol.* 161 1202–1216. 10.1104/pp.112.205385 23296688 PMC3585590

[B129] Jasim Aljumaili S. Rafii M. Latif M. Sakimin S. Z. Arolu I. W. Miah G. (2018). Genetic diversity of aromatic rice germplasm revealed by SSR markers. *BioMed Res. Int.* 2018:7658032. 10.1155/2018/7658032 PMC5874984 29736396

[B130] Jeong J. S. Kim Y. S. Baek K. H. Jung H. Ha S.-H. Do Choi Y. (2010). Root-specific expression of OsNAC10 improves drought tolerance and grain yield in rice under field drought conditions. *Plant Physiol.* 153 185–197. 10.1104/pp.110.154773 20335401 PMC2862432

[B131] Jha G. Rajeshwari R. Sonti R. V. (2007). Functional interplay between two Xanthomonas oryzae pv. oryzae secretion systems in modulating virulence on rice. *Mol. Plant Microbe Interact.* 20 31–40. 10.1094/MPMI-20-0031 17249420

[B132] Ji L. Zhou P. Zhu Y. Liu F. Li R. Qiu Y. (2017). Proteomic analysis of Rice seedlings under cold stress. *Protein J.* 36 299–307. 28555319 10.1007/s10930-017-9721-2

[B133] Jian X. Zhang L. Li G. Zhang L. Wang X. Cao X. (2010). Identification of novel stress-regulated microRNAs from Oryza sativa L. *Genomics* 95 47–55. 10.1016/j.ygeno.2009.08.017 19796675

[B134] Jiang H. Feng Y. Qiu L. Gao G. Zhang Q. He Y. (2020). Identification of Blast Resistance QTLs Based on Two Advanced Backcross Populations in Rice. *Rice* 13 1–12. 10.1186/s12284-020-00392-6 32488495 PMC7266886

[B135] Jiang W. Jin Y.-M. Lee J. Lee K.-I. Piao R. Han L. (2011). Quantitative trait loci for cold tolerance of rice recombinant inbred lines in low temperature environments. *Mol. Cells* 32 579–587. 22080374 10.1007/s10059-011-0186-4 PMC3887680

[B136] Jiang Y. Cai Z. Xie W. Long T. Yu H. Zhang Q. (2012). Rice functional genomics research: progress and implications for crop genetic improvement. *Biotechnol. Adv.* 30 1059–1070. 21888963 10.1016/j.biotechadv.2011.08.013

[B137] Jiao Y. Wang Y. Xue D. Wang J. Yan M. Liu G. (2010). Regulation of OsSPL14 by OsmiR156 defines ideal plant architecture in rice. *Nat. Genet.* 42:541. 10.1038/ng.591 20495565

[B138] Jin Y.-M. Piao R. Yan Y.-F. Chen M. Wang L. He H. (2018). Overexpression of a new zinc finger protein transcription factor OsCTZFP8 improves cold tolerance in rice. *Int. J. Genom.* 2018:5480617. 10.1155/2018/5480617 29951522 PMC5989172

[B139] Jing W. Deng P. Cao C. Zhang W. (2017). Fine mapping of qSKC-1, a major quantitative trait locus for shoot K+ concentration, in rice seedlings grown under salt stress. *Breed. Sci.* 2017:16190. 10.1270/jsbbs.16190 28744182 PMC5515313

[B140] Johnson C. Bowman L. Adai A. T. Vance V. Sundaresan V. (2007). CSRDB: a small RNA integrated database and browser resource for cereals. *Nucleic Acids Res.* 35 D829–D833. 10.1093/nar/gkl991 17169981 PMC1781248

[B141] Joshi R. Wani S. H. Singh B. Bohra A. Dar Z. A. Lone A. A. (2016). Transcription factors and plants response to drought stress: current understanding and future directions. *Front. Plant Sci.* 7:1029. 10.3389/fpls.2016.01029 27471513 PMC4943945

[B142] Junot C. Fenaille F. (2019). “Metabolomics using Fourier transform mass spectrometry,” in *Fundamentals and Applications of Fourier Transform Mass Spectrometry*, eds Schmitt-Kopplin P. Kanawati B. (Amsterdam: Elsevier), 325–356.

[B143] Kandasamy S. Loganathan K. Muthuraj R. Duraisamy S. Seetharaman S. Thiruvengadam R. (2009). Understanding the molecular basis of plant growth promotional effect of *Pseudomonas* fluorescens on rice through protein profiling. *Proteome Sci.* 7 1–8. 10.1186/1477-5956-7-47 20034395 PMC2805620

[B144] Kandaswamy R. Ramasamy M. K. Palanivel R. Balasundaram U. (2019). Impact of *Pseudomonas* putida RRF3 on the root transcriptome of rice plants: Insights into defense response, secondary metabolism and root exudation. *J. Biosci.* 44 1–13. 31502576

[B145] Kanehisa M. Furumichi M. Tanabe M. Sato Y. Morishima K. (2017). KEGG: new perspectives on genomes, pathways, diseases and drugs. *Nucleic Acids Res.* 45 D353–D361. 27899662 10.1093/nar/gkw1092 PMC5210567

[B146] Kar M. M. Raichaudhuri A. (2021). Role of microRNAs in mediating biotic and abiotic stress in plants. *Plant Gene* 26:100277.

[B147] Karmakar S. Datta K. Molla K. A. Gayen D. Das K. Sarkar S. N. (2019). Proteo-metabolomic investigation of transgenic rice unravels metabolic alterations and accumulation of novel proteins potentially involved in defence against Rhizoctonia solani. *Sci. Rep.* 9 1–16. 10.1038/s41598-019-46885-3 31320685 PMC6639406

[B148] Kaul S. Koo H. L. Jenkins J. Rizzo M. Rooney T. Tallon L. J. (2000). Analysis of the genome sequence of the flowering plant Arabidopsis thaliana. *Nature* 408 796–815. 11130711 10.1038/35048692

[B149] Kawahara Y. De La Bastide M. Hamilton J. P. Kanamori H. Mccombie W. R. Ouyang S. (2013). Improvement of the Oryza sativa Nipponbare reference genome using next generation sequence and optical map data. *Rice* 6 1–10. 10.1186/1939-8433-6-4 24280374 PMC5395016

[B150] Kawahara Y. Oono Y. Wakimoto H. Ogata J. Kanamori H. Sasaki H. (2016). TENOR: database for comprehensive mRNA-Seq experiments in rice. *Plant Cell Physiol.* 57 e7–e7. 10.1093/pcp/pcv179 26578693

[B151] Khan M. H. Dar Z. A. Dar S. A. (2015). Breeding strategies for improving rice yield—a review. *Agricult. Sci.* 6:467.

[B152] Khan M. I. R. Jalil S. U. Chopra P. Chhillar H. Ferrante A. Khan N. A. (2021). Role of GABA in plant growth, development and senescence. *Plant Gene* 2021:100283.

[B153] Kim S. T. Kim S. G. Agrawal G. K. Kikuchi S. Rakwal R. (2014). Rice proteomics: a model system for crop improvement and food security. *Proteomics* 14 593–610. 10.1002/pmic.201300388 24323464

[B154] Kim H. Lee K. Hwang H. Bhatnagar N. Kim D.-Y. Yoon I. S. (2014). Overexpression of PYL5 in rice enhances drought tolerance, inhibits growth, and modulates gene expression. *J. Exp. Bot.* 65 453–464. 10.1093/jxb/ert397 24474809 PMC3904710

[B155] Kim S.-M. Reinke R. F. (2018). Identification of QTLs for tolerance to hypoxia during germination in rice. *Euphytica* 214 1–10.

[B156] Kim Y. J. Lee H. M. Wang Y. Wu J. Kim S. G. Kang K. Y. (2013). Depletion of abundant plant R u B is CO protein using the protamine sulfate precipitation method. *Proteomics* 13 2176–2179. 10.1002/pmic.201200555 23576416

[B157] Kim Y. Chung Y. S. Lee E. Tripathi P. Heo S. Kim K.-H. (2020). Root response to drought stress in rice (Oryza sativa L.). *Int. J. Mol. Sci.* 21:1513. 10.3390/ijms21041513 PMC7073213 32098434

[B158] Komatsu S. Tanaka N. (2005). Rice proteome analysis: a step toward functional analysis of the rice genome. *Proteomics* 5 938–949. 15627974 10.1002/pmic.200401040

[B159] Kozomara A. Birgaoanu M. Griffiths-Jones S. (2019). miRBase: from microRNA sequences to function. *Nucleic Acids Res.* 47 D155–D162. 30423142 10.1093/nar/gky1141 PMC6323917

[B160] Kremling K. A. Chen S.-Y. Su M.-H. Lepak N. K. Romay M. C. Swarts K. L. (2018). Dysregulation of expression correlates with rare-allele burden and fitness loss in maize. *Nature* 555 520–523. 10.1038/nature25966 29539638

[B161] Krishnamurthy S. Pundir P. Warraich A. S. Rathor S. Lokeshkumar B. Singh N. K. (2020). Introgressed saltol QTL lines improves the salinity tolerance in rice at seedling stage. *Front. Plant Sci.* 11:833. 10.3389/fpls.2020.00833 32595689 PMC7300257

[B162] Kudo T. Akiyama K. Kojima M. Makita N. Sakurai T. Sakakibara H. (2013). UniVIO: a multiple omics database with hormonome and transcriptome data from rice. *Plant Cell Physiol.* 54 e9–e9. 10.1093/pcp/pct003 23314752 PMC3583028

[B163] Kulkarni S. R. Balachandran S. Ulaganathan K. Balakrishnan D. Praveen M. Prasad A. H. (2020). Molecular mapping of QTLs for yield related traits in recombinant inbred line (RIL) population derived from the popular rice hybrid KRH-2 and their validation through SNP genotyping. *Sci. Rep.* 10 1–21. 10.1038/s41598-020-70637-3 32792551 PMC7427098

[B164] Kumar A. Dash P. K. (2019). “Transcriptome Analysis for Abiotic Stresses in Rice (Oryza sativa L,” in *Transcriptome Analysis*, ed. Blumenberg M. (London: IntechOpen).

[B165] Kumar A. Basu S. Ramegowda V. Pereira A. (2017). *Mechanisms of drought tolerance in rice.* Cambridgeshire: Burleigh Dodds Science Publishing Limited, 131–163.

[B166] Kumar A. Bernier J. Verulkar S. Lafitte H. Atlin G. (2008). Breeding for drought tolerance: direct selection for yield, response to selection and use of drought-tolerant donors in upland and lowland-adapted populations. *Field Crops Res.* 107 221–231.

[B167] Kumar I. S. Nadarajah K. (2020). A Meta-Analysis of Quantitative Trait Loci Associated with Multiple Disease Resistance in Rice (Oryza sativa L.). *Plants* 9:1491. 10.3390/plants9111491 33167299 PMC7694349

[B168] Kumar V. Singh A. Mithra S. A. Krishnamurthy S. Parida S. K. Jain S. (2015). Genome-wide association mapping of salinity tolerance in rice (Oryza sativa). *DNA Res.* 22 133–145. 25627243 10.1093/dnares/dsu046 PMC4401324

[B169] Kurotani A. Yamada Y. Shinozaki K. Kuroda Y. Sakurai T. (2015). Plant-PrAS: a database of physicochemical and structural properties and novel functional regions in plant proteomes. *Plant Cell Physiol.* 56 e11–e11. 10.1093/pcp/pcu176 25435546 PMC4301743

[B170] Kyndt T. Denil S. Haegeman A. Trooskens G. De Meyer T. Van Criekinge W. (2012). Transcriptome analysis of rice mature root tissue and root tips in early development by massive parallel sequencing. *J. Exp. Bot.* 63 2141–2157. 10.1093/jxb/err435 22213813

[B171] Lakra N. Kaur C. Singla-Pareek S. L. Pareek A. (2019). Mapping the ‘early salinity response’triggered proteome adaptation in contrasting rice genotypes using iTRAQ approach. *Rice* 12 1–22. 10.1186/s12284-018-0259-5 30701331 PMC6357216

[B172] Lawas L. M. F. Li X. Erban A. Kopka J. Jagadish S. K. Zuther E. (2019). Metabolic responses of rice cultivars with different tolerance to combined drought and heat stress under field conditions. *GigaScience* 8:giz050. 10.1093/gigascience/giz050 PMC6511916 31081890

[B173] Lee J. Bricker T. M. Lefevre M. Pinson S. R. Oard J. H. (2006). Proteomic and genetic approaches to identifying defence-related proteins in rice challenged with the fungal pathogen Rhizoctonia solani. *Mol. Plant Pathol.* 7 405–416. 10.1111/j.1364-3703.2006.00350.x 20507456

[B174] Lee J. Lee Y. Kim M. Ham T.-H. Jo S.-M. Kwon S.-W. (2017). Quantitative shotgun proteomic analysis of cold-stressed mature rice anthers. *Plant Biotechnol. Rep.* 11 417–427.

[B175] Lee T. Oh T. Yang S. Shin J. Hwang S. Kim C. Y. (2015). RiceNet v2: an improved network prioritization server for rice genes. *Nucleic Acids Res.* 43 W122–W127. 10.1093/nar/gkv253 25813048 PMC4489288

[B176] Lee T.-H. Kim Y.-K. Pham T. T. M. Song S. I. Kim J.-K. Kang K. Y. (2009). RiceArrayNet: a database for correlating gene expression from transcriptome profiling, and its application to the analysis of coexpressed genes in rice. *Plant Physiol.* 151 16–33. 10.1104/pp.109.139030 19605550 PMC2735985

[B177] Lescot M. Déhais P. Thijs G. Marchal K. Moreau Y. Van De Peer Y. (2002). PlantCARE, a database of plant cis-acting regulatory elements and a portal to tools for in silico analysis of promoter sequences. *Nucleic Acids Res.* 30 325–327. 10.1093/nar/30.1.325 11752327 PMC99092

[B178] Li J. Thomson M. Mccouch S. R. (2004). Fine mapping of a grain-weight quantitative trait locus in the pericentromeric region of rice chromosome 3. *Genetics* 168 2187–2195. 10.1534/genetics.104.034165 15611185 PMC1448733

[B179] Li Y. Nie Y. Zhang Z. Ye Z. Zou X. Zhang L. (2014). Comparative proteomic analysis of methyl jasmonate-induced defense responses in different rice cultivars. *Proteomics* 14 1088–1101. 10.1002/pmic.201300104 24505015

[B180] Li J.-Y. Wang J. Zeigler R. S. (2014). The 3,000 rice genomes project: new opportunities and challenges for future rice research. *Gigascience* 3 2217X–2043X. 10.1186/2047-217X-3-8 24872878 PMC4035671

[B181] Li N. Liu H. Sun J. Zheng H. Wang J. Yang L. (2018). Transcriptome analysis of two contrasting rice cultivars during alkaline stress. *Sci. Rep.* 8 1–16. 29941956 10.1038/s41598-018-27940-x PMC6018505

[B182] Li R. Chaicherdsakul T. Kunathigan V. Roytrakul S. Paemanee A. Kittisenachai S. (2020). Shotgun Proteomic Analysis of Germinated Rice (Oryza sativa L.) under Salt Stress. *Appl. Sci. Engine. Prog.* 13 76–85.

[B183] Li S. Xie K. Li W. Zou T. Ren Y. Wang S. (2012). Re-sequencing and genetic variation identification of a rice line with ideal plant architecture. *Rice* 5 1–7. 10.1186/1939-8433-5-18 27234240 PMC5520836

[B184] Li T. Li H. Zhang Y.-X. Liu J.-Y. (2011). Identification and analysis of seven H2O2-responsive miRNAs and 32 new miRNAs in the seedlings of rice (Oryza sativa L. ssp. indica). *Nucleic Acids Res.* 39 2821–2833. 10.1093/nar/gkq1047 21113019 PMC3074118

[B185] Li Y. Ye Z. Nie Y. Zhang J. Wang G.-L. Wang Z. (2015). Comparative phosphoproteome analysis of Magnaporthe oryzae-responsive proteins in susceptible and resistant rice cultivars. *J. Proteom.* 115 66–80. 10.1016/j.jprot.2014.12.007 25540933

[B186] Li Y. Zhao S.-L. Li J.-L. Hu X.-H. Wang H. Cao X.-L. (2017). Osa-miR169 negatively regulates rice immunity against the blast fungus Magnaporthe oryzae. *Front. Plant Sci.* 8:2. 10.3389/fpls.2017.00002 28144248 PMC5239796

[B187] Liang Y. Meng L. Lin X. Cui Y. Pang Y. Xu J. (2018). QTL and QTL networks for cold tolerance at the reproductive stage detected using selective introgression in rice. *PLoS One* 13:e0200846. 10.1371/journal.pone.0200846 30222760 PMC6141068

[B188] Liang Z. Wang L. Pan Q. (2016). A new recessive gene conferring resistance against rice blast. *Rice* 9 1–6. 10.1186/s12284-016-0120-7 27637926 PMC5025421

[B189] Liao J.-L. Zhou H.-W. Zhang H.-Y. Zhong P.-A. Huang Y.-J. (2014). Comparative proteomic analysis of differentially expressed proteins in the early milky stage of rice grains during high temperature stress. *J. Exp. Bot.* 65 655–671. 24376254 10.1093/jxb/ert435 PMC3904723

[B190] Lin D.-G. Chou S.-Y. Wang A. Z. Wang Y.-W. Kuo S.-M. Lai C.-C. (2014). A proteomic study of rice cultivar TNG67 and its high aroma mutant SA0420. *Plant Sci.* 214 20–28. 10.1016/j.plantsci.2013.09.010 24268160

[B191] Lin H. Zhu M. Yano M. Gao J. Liang Z. Su W. (2004). QTLs for Na+ and K+ uptake of the shoots and roots controlling rice salt tolerance. *Theoret. Appl. Genet.* 108 253–260. 10.1007/s00122-003-1421-y 14513218

[B192] Lin Z. Zhang X. Wang Z. Jiang Y. Liu Z. Alexander D. (2017). Metabolomic analysis of pathways related to rice grain chalkiness by a notched-belly mutant with high occurrence of white-belly grains. *BMC Plant Biol.* 17:39. 10.1186/s12870-017-0985-7 28166731 PMC5294873

[B193] Liu C. Mao B. Ou S. Wang W. Liu L. Wu Y. (2014). OsbZIP71, a bZIP transcription factor, confers salinity and drought tolerance in rice. *Plant Mol. Biol.* 84 19–36. 23918260 10.1007/s11103-013-0115-3

[B194] Liu C. W. Chang T. S. Hsu Y. K. Wang A. Z. Yen H. C. Wu Y. P. (2014). Comparative proteomic analysis of early salt stress responsive proteins in roots and leaves of rice. *Proteomics* 14 1759–1775. 10.1002/pmic.201300276 24841874

[B195] Liu K. Wang L. Xu Y. Chen N. Ma Q. Li F. (2007). Overexpression of OsCOIN, a putative cold inducible zinc finger protein, increased tolerance to chilling, salt and drought, and enhanced proline level in rice. *Planta* 226 1007–1016. 10.1007/s00425-007-0548-5 17549515

[B196] Liu Q. Su Y. Zhu Y. Peng K. Hong B. Wang R. (2019). Manipulating osa-MIR156f expression by D18 promoter to regulate plant architecture and yield traits both in seasonal and ratooning rice. *Biol. Proced. Online* 21 1–14. 10.1186/s12575-019-0110-4 31700499 PMC6827258

[B197] Liu Y. Lu S. Liu K. Wang S. Huang L. Guo L. (2019). Proteomics: A powerful tool to study plant responses to biotic stress. *Plant Methods* 15 1–20. 31832077 10.1186/s13007-019-0515-8 PMC6859632

[B198] Liu W.-T. Chen P.-W. Chen L.-C. Yang C.-C. Chen S.-Y. Huang G. (2017). Suppressive effect of microRNA319 expression on rice plant height. *Theoret. Appl. Genet.* 130 1507–1518. 10.1007/s00122-017-2905-5 28470512

[B199] Llorente C. Jimenez R. Brotman Y. Fernie A. R. Sreenivasulu N. (2019). “Rice Grain Quality Benchmarking Through Profiling of Volatiles and Metabolites in Grains Using Gas Chromatography Mass Spectrometry,” in *Rice Grain Quality*, ed. Nese S. (Berlin: Springer), 187–199. 10.1007/978-1-4939-8914-0_11 30397807

[B200] Lowe R. Shirley N. Bleackley M. Dolan S. Shafee T. (2017). Transcriptomics technologies. *PLoS Computat. Biol.* 13:e1005457. 10.1371/journal.pcbi.1005457 28545146 PMC5436640

[B201] Lu T. Lu G. Fan D. Zhu C. Li W. Zhao Q. (2010). Function annotation of the rice transcriptome at single-nucleotide resolution by RNA-seq. *Genome Res.* 20 1238–1249. 10.1101/gr.106120.110 20627892 PMC2928502

[B202] Lu Y. Feng Z. Liu X. Bian L. Xie H. Zhang C. (2018). MiR393 and miR390 synergistically regulate lateral root growth in rice under different conditions. *BMC Plant Biol.* 18:1–12. 10.1186/s12870-018-1488-x 30373525 PMC6206659

[B203] Luo Y.-C. Zhou H. Li Y. Chen J.-Y. Yang J.-H. Chen Y.-Q. (2006). Rice embryogenic calli express a unique set of microRNAs, suggesting regulatory roles of microRNAs in plant post-embryogenic development. *FEBS Lett.* 580 5111–5116. 10.1016/j.febslet.2006.08.046 16959252

[B204] Lv D.-K. Bai X. Li Y. Ding X.-D. Ge Y. Cai H. (2010). Profiling of cold-stress-responsive miRNAs in rice by microarrays. *Gene* 459 39–47. 10.1016/j.gene.2010.03.011 20350593

[B205] Ma Z. Wang L. Zhao M. Gu S. Wang C. Zhao J. (2020). iTRAQ proteomics reveals the regulatory response to Magnaporthe oryzae in durable resistant vs. susceptible rice genotypes. *PLoS One* 15:e0227470. 10.1371/journal.pone.0227470 31923921 PMC6954073

[B206] Ma H. Sheng C. Qiao L. Zhao H. Niu D. (2020). A comparative proteomic approach to identify defence-related proteins between resistant and susceptible rice cultivars challenged with the fungal pathogen Rhizoctonia solani. *Plant Growth Regulat.* 90 73–88.

[B207] Ma X. Xia H. Liu Y. Wei H. Zheng X. Song C. (2016). Transcriptomic and metabolomic studies disclose key metabolism pathways contributing to well-maintained photosynthesis under the drought and the consequent drought-tolerance in rice. *Front. Plant Sci.* 7:1886. 10.3389/fpls.2016.01886 28066455 PMC5174129

[B208] Maksup S. Pongpakpian S. Roytrakul S. (2020). Proteomics of seed nutrition-associated proteins in germinated brown rice in four Thai rice cultivars analyzed by GeLC-MS/MS. *Walailak J. Sci. Technol.* 18:6953.

[B209] Mallikarjuna G. Mallikarjuna K. Reddy M. Kaul T. (2011). Expression of OsDREB2A transcription factor confers enhanced dehydration and salt stress tolerance in rice (Oryza sativa L.). *Biotechnol. Lett.* 33 1689–1697. 10.1007/s10529-011-0620-x 21528404

[B210] Matsaunyane L. B. Dubery I. A. (2018). “Molecular Approaches to Address Intended and Unintended Effects and Substantial Equivalence of Genetically Modified Crops,” in *Transgenic Crops-Emerging Trends and Future Perspectives*, eds Khan M. S. Malik K. A. (London: IntechOpen).

[B211] McCouch S. R. Wright M. H. Tung C.-W. Maron L. G. Mcnally K. L. Fitzgerald M. (2016). Open access resources for genome-wide association mapping in rice. *Nat. Commun.* 7 1–14. 10.1038/ncomms10532 PMC4742900 26842267

[B212] McCouch S. R. Zhao K. Wright M. Tung C.-W. Ebana K. Thomson M. (2010). Development of genome-wide SNP assays for rice. *Breed. Sci.* 60 524–535.

[B213] McNally K. L. Childs K. L. Bohnert R. Davidson R. M. Zhao K. Ulat V. J. (2009). Genomewide SNP variation reveals relationships among landraces and modern varieties of rice. *PNAS* 106 12273–12278. 10.1073/pnas.0900992106 19597147 PMC2718348

[B214] Mei H. Xu J. Li Z. Yu X. Guo L. Wang Y. (2006). QTLs influencing panicle size detected in two reciprocal introgressive line (IL) populations in rice (Oryza sativa L.). *Theoret. Appl. Genet.* 112 648–656. 10.1007/s00122-005-0167-0 16331475

[B215] Meng Q. Gupta R. Min C. W. Kwon S. W. Wang Y. Je B. I. (2019). Proteomics of Rice—Magnaporthe oryzae interaction: what have we learned so far? *Front. Plant Sci.* 10:1383. 10.3389/fpls.2019.01383 31737011 PMC6828948

[B216] Mi H. Huang X. Muruganujan A. Tang H. Mills C. Kang D. (2017). PANTHER version 11: expanded annotation data from Gene Ontology and Reactome pathways, and data analysis tool enhancements. *Nucleic Acids Res.* 45 D183–D189. 10.1093/nar/gkw1138 27899595 PMC5210595

[B217] Mi H. Muruganujan A. Thomas P. D. (2012). PANTHER in 2013: modeling the evolution of gene function, and other gene attributes, in the context of phylogenetic trees. *Nucleic Acids Res.* 41 D377–D386. 10.1093/nar/gks1118 23193289 PMC3531194

[B218] Mishra K. K. Vikram P. Yadaw R. B. Swamy B. M. Dixit S. Cruz M. T. S. (2013). qDTY 12.1: a locus with a consistent effect on grain yield under drought in rice. *BMC Genet.* 14:1–10. 10.1186/1471-2156-14-12 23442150 PMC3616849

[B219] Miura K. Ikeda M. Matsubara A. Song X.-J. Ito M. Asano K. (2010). OsSPL14 promotes panicle branching and higher grain productivity in rice. *Nat. Genet.* 42 545–549. 10.1038/ng.592 20495564

[B220] Mohammadinezhad G. Singh R. Arzani A. Rezaei A. Sabouri H. Gregorio G. (2010). Evaluation of salinity tolerance in rice genotypes. *Int. J. Plant Product.* 4 199–208.

[B221] Mohan V. Spiegelman D. Sudha V. Gayathri R. Hong B. Praseena K. (2014). Effect of brown rice, white rice, and brown rice with legumes on blood glucose and insulin responses in overweight Asian Indians: a randomized controlled trial. *Diabet. Technol. Therapeut.* 16 317–325. 10.1089/dia.2013.0259 24447043 PMC3996977

[B222] Mondal S. Khan M. I. R. Entila F. Dixit S. Cruz P. C. S. Ali M. P. (2020). Responses of AG1 and AG2 QTL introgression lines and seed pre-treatment on growth and physiological processes during anaerobic germination of rice under flooding. *Sci. Rep.* 10 1–15. 10.1038/s41598-020-67240-x 32576897 PMC7311552

[B223] Mondal T. K. Panda A. K. Rawal H. C. Sharma T. R. (2018). Discovery of microRNA-target modules of African rice (Oryza glaberrima) under salinity stress. *Sci. Rep.* 8 1–11. 10.1038/s41598-017-18206-z 29330361 PMC5766505

[B224] Moon S.-J. Min M. K. Kim J. Kim D. Y. Yoon I. S. Kwon T. R. (2019). Ectopic expression of OsDREB1G, a member of the OsDREB1 subfamily, confers cold stress tolerance in rice. *Front. Plant Sci.* 10:297. 10.3389/fpls.2019.00297 30984209 PMC6447655

[B225] Moradi F. Ismail A. Egdane J. Gregorio G. (2003). Salinity tolerance of rice during reproductive development and association with tolerance at the seedling stage. *Ind. J. Plant Physiol.* 8 105–116. 10.1093/aob/mcm052 17428832 PMC3243573

[B226] Morris R. T. O’connor T. R. Wyrick J. J. (2008). Osiris: an integrated promoter database for Oryza sativa L. *Bioinformatics* 24 2915–2917. 10.1093/bioinformatics/btn537 18922805

[B227] Moustafa K. Cross J. (2016). Genetic approaches to study plant responses to environmental stresses: an overview. *Biology* 5:20. 10.3390/biology5020020 PMC4929534 27196939

[B228] Nadarajah K. Kumar I. S. (2019). Drought response in rice: The miRNA story. *Int. J. Mol. Sci.* 20:3766. 10.3390/ijms20153766 31374851 PMC6696311

[B229] Nagaki K. Cheng Z. Ouyang S. Talbert P. B. Kim M. Jones K. M. (2004). Sequencing of a rice centromere uncovers active genes. *Nat. Genet.* 36 138–145. 14716315 10.1038/ng1289

[B230] Naithani S. Gupta P. Preece J. D’eustachio P. Elser J. L. Garg P. (2020). Plant Reactome: a knowledgebase and resource for comparative pathway analysis. *Nucleic Acids Res.* 48 D1093–D1103. 31680153 10.1093/nar/gkz996 PMC7145600

[B231] Naithani S. Preece J. D’eustachio P. Gupta P. Amarasinghe V. Dharmawardhana P. D. (2016). Plant Reactome: a resource for plant pathways and comparative analysis. *Nucleic Acids Res.* 2016:gkw932. 10.1093/nar/gkw932 PMC5210633 27799469

[B232] Nakashima K. Tran L. S. P. Van Nguyen D. Fujita M. Maruyama K. Todaka D. (2007). Functional analysis of a NAC-type transcription factor OsNAC6 involved in abiotic and biotic stress-responsive gene expression in rice. *Plant J.* 51 617–630. 10.1111/j.1365-313X.2007.03168.x 17587305

[B233] Nam K. H. Shin H. J. Pack I. S. Park J. H. Kim H. B. Kim C. G. (2016). Metabolomic changes in grains of well-watered and drought-stressed transgenic rice. *J. Sci. Food Agricult.* 96 807–814. 10.1002/jsfa.7152 25716954

[B234] Niones J. M. (2004). *Five mapping of the salinity tolerance gene on chromosome 1 of rice (Oryza sativa L.) using near-isogenic lines.* Geneva: FAO.

[B235] Norvienyeku J. Lin L. Waheed A. Chen X. Bao J. Aliyu S. R. (2021). Bayogenin 3-O-cellobioside confers non-cultivar-specific defence against the rice blast fungus Pyricularia oryzae. *Plant Biotechnol. J.* 19:589. 10.1111/pbi.13488 33043566 PMC7955875

[B236] Obayashi T. Aoki Y. Tadaka S. Kagaya Y. Kinoshita K. (2018). ATTED-II in 2018: a plant coexpression database based on investigation of the statistical property of the mutual rank index. *Plant Cell Physiol.* 59 e3–e3. 29216398 10.1093/pcp/pcx191 PMC5914358

[B237] Ogata Y. Suzuki H. Sakurai N. Shibata D. (2010). CoP: a database for characterizing co-expressed gene modules with biological information in plants. *Bioinformatics* 26 1267–1268. 10.1093/bioinformatics/btq121 20305269

[B238] Ohyanagi H. Ebata T. Huang X. Gong H. Fujita M. Mochizuki T. (2016). OryzaGenome: genome diversity database of wild Oryza species. *Plant Cell Physiol.* 57 e1–e1. 26578696 10.1093/pcp/pcv171 PMC4722174

[B239] Ohyanagi H. Tanaka T. Sakai H. Shigemoto Y. Yamaguchi K. Habara T. (2006). The Rice Annotation Project Database (RAP-DB): hub for Oryza sativa ssp. japonica genome information. *Nucleic Acids Res.* 34 D741–D744. 10.1093/nar/gkj094 16381971 PMC1347456

[B240] Oikawa A. Matsuda F. Kusano M. Okazaki Y. Saito K. (2008). Rice metabolomics. *Rice* 1 63–71.

[B241] Okazaki Y. Saito K. (2016). Integrated metabolomics and phytochemical genomics approaches for studies on rice. *GigaScience* 5 s13742–s13016. 10.1186/s13742-016-0116-7 PMC4774183 26937280

[B242] Ouyang S. Zhu W. Hamilton J. Lin H. Campbell M. Childs K. (2007). The TIGR rice genome annotation resource: improvements and new features. *Nucleic Acids Res.* 35 D883–D887. 10.1093/nar/gkl976 17145706 PMC1751532

[B243] Pandey A. Sonti R. V. (2010). Role of the FeoB protein and siderophore in promoting virulence of Xanthomonas oryzae pv. oryzae on rice. *J. Bacteriol.* 192 3187–3203. 10.1128/JB.01558-09 20382771 PMC2901680

[B244] Pandey A. Rajamani U. Verma J. Subba P. Chakraborty N. Datta A. (2010). Identification of extracellular matrix proteins of rice (Oryza sativa L.) involved in dehydration-responsive network: a proteomic approach. *J. Proteome Res.* 9 3443–3464. 10.1021/pr901098p 20433195

[B245] Pandit A. Rai V. Bal S. Sinha S. Kumar V. Chauhan M. (2010). Combining QTL mapping and transcriptome profiling of bulked RILs for identification of functional polymorphism for salt tolerance genes in rice (Oryza sativa L.). *Mol. Genet. Genom.* 284 121–136. 10.1007/s00438-010-0551-6 20602115

[B246] Pantalião G. F. Vianello R. P. Bueno L. G. Mendonça J. A. Coelho A. S. G. Cordeiro A. C. C. (2020). Development of SNP markers for grain yield screening of Brazilian rice cultivars. *Pesquisa Agropecuária Brasileira* 55:1643.

[B247] Papatheodorou I. Fonseca N. A. Keays M. Tang Y. A. Barrera E. Bazant W. (2018). Expression Atlas: gene and protein expression across multiple studies and organisms. *Nucleic Acids Res.* 46 D246–D251. 29165655 10.1093/nar/gkx1158 PMC5753389

[B248] Park J.-R. Yang W.-T. Kwon Y.-S. Kim H.-N. Kim K.-M. Kim D.-H. (2019). Assessment of the Genetic Diversity of Rice Germplasms Characterized by Black-Purple and Red Pericarp Color Using Simple Sequence Repeat Markers. *Plants* 8:471. 10.3390/plants8110471 31689922 PMC6918417

[B249] Parkinson J. Blaxter M. (2009). Expressed sequence tags: an overview. *Expressed Sequence Tags* 2009 1–12. 10.1007/978-1-60327-136-3_1 19277571

[B250] Parmar S. Gharat S. A. Tagirasa R. Chandra T. Behera L. Dash S. K. (2020). Identification and expression analysis of miRNAs and elucidation of their role in salt tolerance in rice varieties susceptible and tolerant to salinity. *PLoS One* 15:e0230958. 10.1371/journal.pone.0230958 32294092 PMC7159242

[B251] Peng Yuan D. Xu X. F. Hong W.-J. Wang S. T. Jia X. T. Liu Y. (2020). Transcriptome analysis of rice leaves in response to Rhizoctonia solani infection and reveals a novel regulatory mechanism. *Plant Biotechnol. Rep.* 14 559–573.

[B252] Qi L. Sun Y. Li J. Su L. Zheng X. Wang X. (2017). Identify QTLs for grain size and weight in common wild rice using chromosome segment substitution lines across six environments. *Breed. Sci.* 2017:16082. 10.1270/jsbbs.16082 29398941 PMC5790038

[B253] Que S. Li K. Chen M. Wang Y. Yang Q. Zhang W. (2012). Phospho Rice: a meta-predictor of rice-specific phosphorylation sites. *Plant Methods* 8 1–9. 10.1186/1746-4811-8-5 22305189 PMC3395875

[B254] Quek X. C. Thomson D. W. Maag J. L. Bartonicek N. Signal B. Clark M. B. (2015). lncRNAdb v2. 0: expanding the reference database for functional long noncoding RNAs. *Nucleic Acids Res.* 43 D168–D173. 10.1093/nar/gku988 25332394 PMC4384040

[B255] Rahman H. Ramanathan V. Nallathambi J. Duraialagaraja S. Muthurajan R. (2016). Over-expression of a NAC 67 transcription factor from finger millet (Eleusine coracana L.) confers tolerance against salinity and drought stress in rice. *BMC Biotechnol.* 16:7–20. 10.1186/s12896-016-0261-1 27213684 PMC4896240

[B256] Raineri J. Wang S. Peleg Z. Blumwald E. Chan R. L. (2015). The rice transcription factor OsWRKY47 is a positive regulator of the response to water deficit stress. *Plant Mol. Biol.* 88 401–413. 10.1007/s11103-015-0329-7 25957211

[B257] Ranawake A. L. Manangkil O. E. Yoshida S. Ishii T. Mori N. Nakamura C. (2014). Mapping QTLs for cold tolerance at germination and the early seedling stage in rice (Oryza sativa L.). *Biotechnol. Biotechnol. Equipm.* 28 989–998. 10.1080/13102818.2014.978539 PMC4684067 26740779

[B258] Rao V. S. Srinivas K. Sujini G. Kumar G. (2014). Protein-protein interaction detection: methods and analysis. *Int. J. Proteom.* 2014:147648. 10.1155/2014/147648 PMC3947875 24693427

[B259] Raza A. Razzaq A. Mehmood S. S. Zou X. Zhang X. Lv Y. (2019). Impact of climate change on crops adaptation and strategies to tackle its outcome: A review. *Plants* 8:34. 10.3390/plants8020034 30704089 PMC6409995

[B260] Raza Q. Riaz A. Saher H. Bibi A. Raza M. A. Ali S. S. (2020). Grain Fe and Zn contents linked SSR markers based genetic diversity in rice. *PLoS One* 15:e0239739. 10.1371/journal.pone.0239739 32986755 PMC7521695

[B261] Reig-Valiente J. L. Marqués L. Talón M. Domingo C. (2018). Genome-wide association study of agronomic traits in rice cultivated in temperate regions. *BMC Genom.* 19:1–11. 10.1186/s12864-018-5086-y 30253735 PMC6156875

[B262] Ren Z. Qi D. Pugh N. Li K. Wen B. Zhou R. (2019). Improvements to the rice genome annotation through large-scale analysis of RNA-Seq and proteomics data sets. *Mol. Cell. Proteom.* 18 86–98. 10.1074/mcp.RA118.000832 30293062 PMC6317475

[B263] Ren Z.-H. Gao J.-P. Li L.-G. Cai X.-L. Huang W. Chao D.-Y. (2005). A rice quantitative trait locus for salt tolerance encodes a sodium transporter. *Nat. Genet.* 37 1141–1146. 16155566 10.1038/ng1643

[B264] Rouard M. Guignon V. Aluome C. Laporte M.-A. Droc G. Walde C. (2011). GreenPhylDB v2. 0: comparative and functional genomics in plants. *Nucleic Acids Res.* 39 D1095–D1102. 10.1093/nar/gkq811 20864446 PMC3013755

[B265] Roy S. C. Lachagari V. B. R. (2017). Assessment of SNP and InDel Variations Among Rice Lines of Tulaipanji x Ranjit. *Rice Sci.* 24 336–348.

[B266] Sabot F. Picault N. El-Baidouri M. Llauro C. Chaparro C. Piegu B. (2011). Transpositional landscape of the rice genome revealed by paired-end mapping of high-throughput re-sequencing data. *Plant J.* 66 241–246. 10.1111/j.1365-313X.2011.04492.x 21219509

[B267] Sahebi M. Hanafi M. M. Azizi P. Hakim A. Ashkani S. Abiri R. (2015). Suppression subtractive hybridization versus next-generation sequencing in plant genetic engineering: challenges and perspectives. *Mol. Biotechnol.* 57 880–903. 10.1007/s12033-015-9884-z 26271955

[B268] Sakai H. Lee S. S. Tanaka T. Numa H. Kim J. Kawahara Y. (2013). Rice Annotation Project Database (RAP-DB): an integrative and interactive database for rice genomics. *Plant Cell Physiol.* 54 e6–e6. 10.1093/pcp/pcs183 23299411 PMC3583025

[B269] Sapkota A. Liu X. Zhao X.-M. Cao Y. Liu J. Liu Z.-P. (2011). DIPOS: database of interacting proteins in Oryza sativa. *Mol. BioSyst.* 7 2615–2621. 10.1039/c1mb05120b 21713282

[B270] Sasaki T. (2005). The map-based sequence of the rice genome. *Nature* 436 793–800. 16100779 10.1038/nature03895

[B271] Sato Y. Namiki N. Takehisa H. Kamatsuki K. Minami H. Ikawa H. (2013a). RiceFREND: a platform for retrieving coexpressed gene networks in rice. *Nucleic Acids Res.* 41 D1214–D1221. 10.1093/nar/gks1122 23180784 PMC3531108

[B272] Sato Y. Takehisa H. Kamatsuki K. Minami H. Namiki N. Ikawa H. (2013b). RiceXPro version 3.0: expanding the informatics resource for rice transcriptome. *Nucleic Acids Res.* 41 D1206–D1213. 10.1093/nar/gks1125 23180765 PMC3531122

[B273] Schläpfer P. Zhang P. Wang C. Kim T. Banf M. Chae L. (2017). Genome-wide prediction of metabolic enzymes, pathways, and gene clusters in plants. *Plant Physiol.* 173 2041–2059. 28228535 10.1104/pp.16.01942 PMC5373064

[B274] Septiningsih E. Prasetiyono J. Lubis E. Tai T. Tjubaryat T. Moeljopawiro S. (2003). Identification of quantitative trait loci for yield and yield components in an advanced backcross population derived from the Oryza sativa variety IR64 and the wild relative O. rufipogon. *Theoret. Appl. Genet.* 107 1419–1432. 10.1007/s00122-003-1373-2 14513215

[B275] Sew Y. S. Aizat W. M. Ab Razak M. S. F. Zainal-Abidin R.-A. Simoh S. Abu-Bakar N. (2020). Comprehensive proteomics data on whole rice grain of selected pigmented and non-pigmented rice varieties using SWATH-MS approach. *Data Brief* 31:105927. 10.1016/j.dib.2020.105927 32642524 PMC7334386

[B276] Shah T. R. Misra A. (2011). “Proteomics,” in *Challenges in Delivery of Therapeutic Genomics and Proteomics*, ed. Misra A. (Amsterdam: Elsevier), 387–427.

[B277] Shanmugavadivel P. Sv A. M. Prakash C. Ramkumar M. Tiwari R. Mohapatra T. (2017). High resolution mapping of QTLs for heat tolerance in rice using a 5K SNP array. *Rice* 10 1–11. 10.1186/s12284-017-0167-0 28584974 PMC5459777

[B278] Shen Y.-J. Jiang H. Jin J.-P. Zhang Z.-B. Xi B. He Y.-Y. (2004). Development of genome-wide DNA polymorphism database for map-based cloning of rice genes. *Plant Physiol.* 135 1198–1205. 10.1104/pp.103.038463 15266053 PMC519040

[B279] Sheteiwy M. S. Shao H. Qi W. Hamoud Y. A. Shaghaleh H. Khan N. U. (2019). GABA-alleviated oxidative injury induced by salinity, osmotic stress and their combination by regulating cellular and molecular signals in rice. *Int. J. Mol. Sci.* 20:5709. 10.3390/ijms20225709 31739540 PMC6888568

[B280] Shomura A. Izawa T. Ebana K. Ebitani T. Kanegae H. Konishi S. (2008). Deletion in a gene associated with grain size increased yields during rice domestication. *Nat. Genet.* 40 1023–1028. 10.1038/ng.169 18604208

[B281] Song S. Tian D. Zhang Z. Hu S. Yu J. (2018). Rice genomics: over the past two decades and into the future. *Genomics Proteom. Bioinformat.* 16 397–404. 10.1016/j.gpb.2019.01.001 PMC6411948 30771506

[B282] Song E.-H. Jeong J. Park C. Y. Kim H.-Y. Kim E.-H. Bang E. (2018). Metabotyping of rice (Oryza sativa L.) for understanding its intrinsic physiology and potential eating quality. *Food Res. Int.* 111 20–30. 10.1016/j.foodres.2018.05.003 30007677

[B283] Song J.-M. Lei Y. Shu C.-C. Ding Y. Xing F. Liu H. (2018). Rice information GateWay: a comprehensive bioinformatics platform for Indica Rice genomes. *Mol. Plant* 11 505–507. 10.1016/j.molp.2017.10.003 29042268

[B284] Song X.-J. Huang W. Shi M. Zhu M.-Z. Lin H.-X. (2007). A QTL for rice grain width and weight encodes a previously unknown RING-type E3 ubiquitin ligase. *Nat. Genet.* 39 623–630. 10.1038/ng2014 17417637

[B285] Sonnhammer E. L. Östlund G. (2015). InParanoid 8: orthology analysis between 273 proteomes, mostly eukaryotic. *Nucleic Acids Res.* 43 D234–D239. 10.1093/nar/gku1203 25429972 PMC4383983

[B286] Stein J. C. Yu Y. Copetti D. Zwickl D. J. Zhang L. Zhang C. (2018). Genomes of 13 domesticated and wild rice relatives highlight genetic conservation, turnover and innovation across the genus Oryza. *Nat. Genet.* 50 285–296. 29358651 10.1038/s41588-018-0040-0

[B287] Suharti W. S. Nose A. Zheng S.-H. (2016). Metabolite profiling of sheath blight disease resistance in rice: in the case of positive ion mode analysis by CE/TOF-MS. *Plant Product. Sci.* 19 279–290.

[B288] Sun C. Hu Z. Zheng T. Lu K. Zhao Y. Wang W. (2017). RPAN: rice pan-genome browser for∼ 3000 rice genomes. *Nucleic Acids Res.* 45 597–605. 10.1093/nar/gkw958 27940610 PMC5314802

[B289] Sun L. Wang J. Song K. Sun Y. Qin Q. Xue Y. (2019). Transcriptome analysis of rice (Oryza sativa L.) shoots responsive to cadmium stress. *Sci. Rep.* 9 1–10. 31308454 10.1038/s41598-019-46684-w PMC6629703

[B290] Sun S.-J. Guo S.-Q. Yang X. Bao Y.-M. Tang H.-J. Sun H. (2010). Functional analysis of a novel Cys2/His2-type zinc finger protein involved in salt tolerance in rice. *J. Exp. Bot.* 61 2807–2818. 10.1093/jxb/erq120 20460361 PMC2882275

[B291] Sun Y. Shi Y. Liu G. Yao F. Zhang Y. Yang C. (2020). Natural variation in the OsbZIP18 promoter contributes to branched-chain amino acid levels in rice. *N. Phytol.* 228 1548–1558. 10.1111/nph.16800 32654152

[B292] Swamy B. M. Vikram P. Dixit S. Ahmed H. Kumar A. (2011). Meta-analysis of grain yield QTL identified during agricultural drought in grasses showed consensus. *BMC Genomics* 12:319. 10.1186/1471-2164-12-319 21679437 PMC3155843

[B293] Szklarczyk D. Morris J. H. Cook H. Kuhn M. Wyder S. Simonovic M. (2016). The STRING database in 2017: quality-controlled protein–protein association networks, made broadly accessible. *Nucleic Acids Res.* 2016:gkw937. 10.1093/nar/gkw937 27924014 PMC5210637

[B294] Takasaki H. Maruyama K. Kidokoro S. Ito Y. Fujita Y. Shinozaki K. (2010). The abiotic stress-responsive NAC-type transcription factor OsNAC5 regulates stress-inducible genes and stress tolerance in rice. *Mol. Genet. Genom.* 284 173–183. 10.1007/s00438-010-0557-0 20632034

[B295] Takeda T. Suwa Y. Suzuki M. Kitano H. Ueguchi-Tanaka M. Ashikari M. (2003). The OsTB1 gene negatively regulates lateral branching in rice. *Plant J.* 33 513–520. 12581309 10.1046/j.1365-313x.2003.01648.x

[B296] Takehisa H. Sato Y. Igarashi M. Abiko T. Antonio B. A. Kamatsuki K. (2012). Genome-wide transcriptome dissection of the rice root system: implications for developmental and physiological functions. *Plant J.* 69 126–140. 10.1111/j.1365-313X.2011.04777.x 21895812

[B297] Tang L. Zhang F. Liu A. Sun J. Mei S. Wang X. (2019). Genome-Wide Association Analysis Dissects the Genetic Basis of the Grain Carbon and Nitrogen Contents in Milled Rice. *Rice* 12 1–16. 10.1186/s12284-019-0362-2 31889226 PMC6937365

[B298] Tang N. Zhang H. Li X. Xiao J. Xiong L. (2012). Constitutive activation of transcription factor OsbZIP46 improves drought tolerance in rice. *Plant Physiol.* 158 1755–1768. 22301130 10.1104/pp.111.190389 PMC3320183

[B299] Tello-Ruiz M. K. Naithani S. Gupta P. Olson A. Wei S. Preece J. (2021). Gramene 2021: harnessing the power of comparative genomics and pathways for plant research. *Nucleic Acids Res.* 49 D1452–D1463. 10.1093/nar/gkaa979 33170273 PMC7779000

[B300] Thomas P. D. Campbell M. J. Kejariwal A. Mi H. Karlak B. Daverman R. (2003). PANTHER: a library of protein families and subfamilies indexed by function. *Genome Res.* 13 2129–2141. 10.1101/gr.772403 12952881 PMC403709

[B301] Thomson M. J. De Ocampo M. Egdane J. Rahman M. A. Sajise A. G. Adorada D. L. (2010). Characterizing the Saltol quantitative trait locus for salinity tolerance in rice. *Rice* 3 148–160.

[B302] Tian D. Yang L. Chen Z. Chen Z. Wang F. Zhou Y. (2018). Proteomic analysis of the defense response to Magnaporthe oryzae in rice harboring the blast resistance gene Piz-t. *Rice* 11 1–13. 10.1186/s12284-018-0240-3 30112588 PMC6093832

[B303] Tian T. Liu Y. Yan H. You Q. Yi X. Du Z. (2017). agriGO v2. 0: a GO analysis toolkit for the agricultural community, 2017 update. *Nucleic Acids Res.* 45 W122–W129. 10.1093/nar/gkx382 28472432 PMC5793732

[B304] Tomcal M. Stiffler N. Barkan A. (2013). POGs2: a web portal to facilitate cross-species inferences about protein architecture and function in plants. *PLoS One* 8:e82569. 10.1371/journal.pone.0082569 24340041 PMC3858315

[B305] Tulpan D. Leger S. (2017). The Plant Orthology Browser: An Orthology and Gene-Order Visualizer for Plant Comparative Genomics. *Plant Genome* 10:0078. 10.3835/plantgenome2016.08.0078 28464063

[B306] Uawisetwathana U. Karoonuthaisiri N. (2019). Metabolomics for rice quality and traceability: feasibility and future aspects. *Curr. Opin. Food Sci.* 28 58–66.

[B307] Upadhyaya H. Panda S. K. (2019). “Drought stress responses and its management in rice,” in *Advances in Rice Research for Abiotic Stress Tolerance*, eds Hasanuzzaman M. Fujita M. Nahar K. Biswas J. K. (Amsterdam: Elsevier), 177–200.

[B308] Usadel B. Obayashi T. Mutwil M. Giorgi F. M. Bassel G. W. Tanimoto M. (2009a). Co-expression tools for plant biology: opportunities for hypothesis generation and caveats. *Plant Cell Environ.* 32 1633–1651. 10.1111/j.1365-3040.2009.02040.x 19712066

[B309] Usadel B. Poree F. Nagel A. Lohse M. Czedik-Eysenberg A. Stitt M. (2009b). A guide to using MapMan to visualize and compare Omics data in plants: a case study in the crop species, Maize. *Plant Cell Environ.* 32 1211–1229. 10.1111/j.1365-3040.2009.01978.x 19389052

[B310] Usman B. Nawaz G. Zhao N. Liu Y. Li R. (2020). Generation of high yielding and fragrant rice (Oryza sativa L.) Lines by CRISPR/Cas9 targeted mutagenesis of three homoeologs of cytochrome P450 gene family and OsBADH2 and transcriptome and proteome profiling of revealed changes triggered by mutations. *Plants* 9:788. 10.3390/plants9060788 32586052 PMC7355857

[B311] Valette M. Rey M. Gerin F. Comte G. Wisniewski-Dyé F. (2020). A common metabolomic signature is observed upon inoculation of rice roots with various rhizobacteria. *J. Integrat. Plant Biol.* 62 228–246. 10.1111/jipb.12810 30920733

[B312] Venuprasad R. Bool M. Quiatchon L. Atlin G. (2012a). A QTL for rice grain yield in aerobic environments with large effects in three genetic backgrounds. *Theoret. Appl. Genet.* 124 323–332. 10.1007/s00122-011-1707-4 21938473

[B313] Venuprasad R. Bool M. Quiatchon L. Cruz M. S. Amante M. Atlin G. (2012b). A large-effect QTL for rice grain yield under upland drought stress on chromosome 1. *Mol. Breed.* 30 535–547.

[B314] Venuprasad R. Dalid C. Del Valle M. Zhao D. Espiritu M. Cruz M. S. (2009). Identification and characterization of large-effect quantitative trait loci for grain yield under lowland drought stress in rice using bulk-segregant analysis. *Theoret. Appl. Genet.* 120 177–190. 10.1007/s00122-009-1168-1 19841886

[B315] Venuprasad R. Lafitte H. R. Atlin G. N. (2007). Response to direct selection for grain yield under drought stress in rice. *Crop Sci.* 47 285–293.

[B316] Vikram P. Swamy B. M. Dixit S. Ahmed H. U. Cruz M. T. S. Singh A. K. (2011). qDTY 1.1, a major QTL for rice grain yield under reproductive-stage drought stress with a consistent effect in multiple elite genetic backgrounds. *BMC Genet.* 12:89. 10.1186/1471-2156-12-89 22008150 PMC3234187

[B317] Vikram P. Swamy B. M. Dixit S. Trinidad J. Sta Cruz M. T. Maturan P. C. (2016). Linkages and interactions analysis of major effect drought grain yield QTLs in rice. *PLoS One* 11:e0151532. 10.1371/journal.pone.0151532 27018583 PMC4809569

[B318] Vo K. T. X. Rahman M. M. Rahman M. M. Trinh K. T. T. Kim S. T. Jeon J.-S. (2021). Proteomics and Metabolomics Studies on the Biotic Stress Responses of Rice: an Update. *Rice* 14 1–16. 10.1186/s12284-021-00461-4 33721115 PMC7960847

[B319] Volante A. Tondelli A. Desiderio F. Abbruscato P. Menin B. Biselli C. (2020). Genome wide association studies for japonica rice resistance to blast in field and controlled conditions. *Rice* 13 1–17. 10.1186/s12284-020-00431-2 33030605 PMC7544789

[B320] Wan X. Wan J. Jiang L. Wang J. Zhai H. Weng J. (2006). QTL analysis for rice grain length and fine mapping of an identified QTL with stable and major effects. *Theoret. Appl. Genet.* 112 1258–1270. 16477428 10.1007/s00122-006-0227-0

[B321] Wang J. Wang J. Wang X. Li R. Chen B. (2018). Proteomic response of hybrid wild rice to cold stress at the seedling stage. *PLoS One* 13:e0198675. 10.1371/journal.pone.0198675 29879216 PMC5991693

[B322] Wang Z. Xia Y. Lin S. Wang Y. Guo B. Song X. (2018). Osa-miR164a targets Os NAC 60 and negatively regulates rice immunity against the blast fungus Magnaporthe oryzae. *Plant J.* 95 584–597. 10.1111/tpj.13972 29775494

[B323] Wang D. R. Agosto-Pérez F. J. Chebotarov D. Shi Y. Marchini J. Fitzgerald M. (2018). An imputation platform to enhance integration of rice genetic resources. *Nat. Commun.* 9 1–10. 10.1038/s41467-018-05538-1 30158584 PMC6115364

[B324] Wang L. Xie W. Chen Y. Tang W. Yang J. Ye R. (2010). A dynamic gene expression atlas covering the entire life cycle of rice. *Plant J.* 61 752–766. 10.1111/j.1365-313X.2009.04100.x 20003165

[B325] Wang M. Lu X. Xu G. Yin X. Cui Y. Huang L. (2016). OsSGL, a novel pleiotropic stress-related gene enhances grain length and yield in rice. *Sci. Rep.* 6 1–12. 10.1038/srep38157 27917884 PMC5137154

[B326] Wang Q. Guan Y. Wu Y. Chen H. Chen F. Chu C. (2008). Overexpression of a rice OsDREB1F gene increases salt, drought, and low temperature tolerance in both Arabidopsis and rice. *Plant Mol. Biol.* 67 589–602. 10.1007/s11103-008-9340-6 18470484

[B327] Wang S. Wu K. Yuan Q. Liu X. Liu Z. Lin X. (2012). Control of grain size, shape and quality by OsSPL16 in rice. *Nat. Genet.* 44:950. 10.1038/ng.2327 22729225

[B328] Wang S.-T. Sun X.-L. Hoshino Y. Yu Y. Jia B. Sun Z.-W. (2014). MicroRNA319 positively regulates cold tolerance by targeting OsPCF6 and OsTCP21 in rice (Oryza sativa L.). *PLoS One* 9:e91357. 10.1371/journal.pone.0091357 24667308 PMC3965387

[B329] Wang Y. Huang L. Du F. Wang J. Zhao X. Li Z. (2021). Comparative transcriptome and metabolome profiling reveal molecular mechanisms underlying OsDRAP1-mediated salt tolerance in rice. *Sci. Rep.* 11 1–11. 10.1038/s41598-021-84638-3 33664392 PMC7933422

[B330] Wang Y. Wang J. Zhao X. Yang S. Huang L. Du F. (2020). Overexpression of the Transcription Factor Gene OsSTAP1 Increases Salt Tolerance in Rice. *Rice* 13 1–12. 10.1186/s12284-020-00405-4 32705427 PMC7378131

[B331] Wang Y.-J. Zhang Z.-G. He X.-J. Zhou H.-L. Wen Y.-X. Dai J.-X. (2003). A rice transcription factor OsbHLH1 is involved in cold stress response. *Theoret. Appl. Genet.* 107 1402–1409. 10.1007/s00122-003-1378-x 12920519

[B332] Wang Z. Gerstein M. Snyder M. (2009). RNA-Seq: a revolutionary tool for transcriptomics. *Nat. Rev. Genet.* 10 57–63. 19015660 10.1038/nrg2484 PMC2949280

[B333] Wanichthanarak K. Boonchai C. Kojonna T. Chadchawan S. Sangwongchai W. Thitisaksakul M. (2020). Deciphering rice metabolic flux reprograming under salinity stress via in silico metabolic modeling. *Computat. Struct. Biotechnol. J.* 18 3555–3566. 10.1016/j.csbj.2020.11.023 33304454 PMC7708941

[B334] Wei J. Wang A. Li R. Qu H. Jia Z. (2018). Metabolome-wide association studies for agronomic traits of rice. *Heredity* 120 342–355. 10.1038/s41437-017-0032-3 29225351 PMC5842221

[B335] Winter D. Vinegar B. Nahal H. Ammar R. Wilson G. V. Provart N. J. (2007). An “Electronic Fluorescent Pictograph” browser for exploring and analyzing large-scale biological data sets. *PLoS One* 2:e718. 10.1371/journal.pone.0000718 17684564 PMC1934936

[B336] Wongpia A. Roytrakul S. Nomura M. Tajima S. Lomthaisong K. Mahatheeranont S. (2016). Proteomic Analysis of Isogenic Rice Reveals Proteins Correlated with Aroma Compound Biosynthesis at Different Developmental Stages. *Mol. Biotechnol.* 58 117–129. 10.1007/s12033-015-9906-x 26646386

[B337] Wu J. Wang Y. Park S.-Y. Kim S. G. Yoo J. S. Park S. (2016). Secreted alpha-N-Arabinofuranosidase B protein is required for the full virulence of Magnaporthe oryzae and triggers host Defences. *PLoS One* 11:e0165149. 10.1371/journal.pone.0165149 27764242 PMC5072668

[B338] Wu J. Yamagata H. Hayashi-Tsugane M. Hijishita S. Fujisawa M. Shibata M. (2004). Composition and structure of the centromeric region of rice chromosome 8. *Plant Cell* 16 967–976. 15037733 10.1105/tpc.019273 PMC412870

[B339] Xia K. Wang R. Ou X. Fang Z. Tian C. Duan J. (2012). OsTIR1 and OsAFB2 downregulation via OsmiR393 overexpression leads to more tillers, early flowering and less tolerance to salt and drought in rice. *PLoS One* 7:e30039. 10.1371/journal.pone.0030039 22253868 PMC3254625

[B340] Xia L. Zou D. Sang J. Xu X. Yin H. Li M. (2017). Rice Expression Database (RED): An integrated RNA-Seq-derived gene expression database for rice. *J. Genet. Genom.* 44 235–241. 10.1016/j.jgg.2017.05.003 28529082

[B341] Xiao B. Huang Y. Tang N. Xiong L. (2007). Over-expression of a LEA gene in rice improves drought resistance under the field conditions. *Theoret. Appl. Genet.* 115 35–46. 10.1007/s00122-007-0538-9 17426956

[B342] Xie K. Shen J. Hou X. Yao J. Li X. Xiao J. (2012). Gradual increase of miR156 regulates temporal expression changes of numerous genes during leaf development in rice. *Plant Physiol.* 158 1382–1394. 10.1104/pp.111.190488 22271747 PMC3291253

[B343] Xie K. Wu C. Xiong L. (2006). Genomic organization, differential expression, and interaction of SQUAMOSA promoter-binding-like transcription factors and microRNA156 in rice. *Plant Physiol.* 142 280–293. 10.1104/pp.106.084475 16861571 PMC1557610

[B344] Xie Z. Wang C. Zhu S. Wang W. Xu J. Zhao X. (2020). Characterizing the metabolites related to rice salt tolerance with introgression lines exhibiting contrasting performances in response to saline conditions. *Plant Growth Regulat.* 92 157–167.

[B345] Xu D. Mou G. Wang K. Zhou G. (2014). MicroRNAs responding to southern rice black-streaked dwarf virus infection and their target genes associated with symptom development in rice. *Virus Res.* 190 60–68. 10.1016/j.virusres.2014.07.007 25038403

[B346] Xu D.-Q. Huang J. Guo S.-Q. Yang X. Bao Y.-M. Tang H.-J. (2008). Overexpression of a TFIIIA-type zinc finger protein gene ZFP252 enhances drought and salt tolerance in rice (Oryza sativa L.). *FEBS Lett.* 582 1037–1043. 10.1016/j.febslet.2008.02.052 18325341

[B347] Xu E. Chen M. He H. Zhan C. Cheng Y. Zhang H. (2017). Proteomic analysis reveals proteins involved in seed imbibition under salt stress in rice. *Front. Plant Sci.* 7:2006. 10.3389/fpls.2016.02006 28105039 PMC5213780

[B348] Xu K. Xu X. Fukao T. Canlas P. Maghirang-Rodriguez R. Heuer S. (2006). Sub1A is an ethylene-response-factor-like gene that confers submergence tolerance to rice. *Nature* 442 705–708. 16900200 10.1038/nature04920

[B349] Xue L.-J. Zhang J.-J. Xue H.-W. (2009). Characterization and expression profiles of miRNAs in rice seeds. *Nucleic Acids Res.* 37 916–930. 19103661 10.1093/nar/gkn998 PMC2647296

[B350] Yadav A. K. Kumar A. Grover N. Ellur R. K. Krishnan S. G. Bollinedi H. (2020). Marker aided introgression of ‘Saltol’, a major QTL for seedling stage salinity tolerance into an elite Basmati rice variety ‘Pusa Basmati 1509’. *Sci. Rep.* 10 1–15. 10.1038/s41598-020-70664-0 32887905 PMC7474085

[B351] Yadav M. K. Ngangkham U. Subudhi H. Bag M. K. Adak T. Munda S. (2017). Correction: Use of molecular markers in identification and characterization of resistance to rice blast in India. *PLoS One* 12:e0179467. 10.1371/journal.pone.0179467 28591200 PMC5462471

[B352] Yadav S. Sandhu N. Singh V. K. Catolos M. Kumar A. (2019). Genotyping-by-sequencing based QTL mapping for rice grain yield under reproductive stage drought stress tolerance. *Sci. Rep.* 9 1–12. 31586108 10.1038/s41598-019-50880-z PMC6778106

[B353] Yamamoto Y. Y. Obokata J. (2007). PPDB: a plant promoter database. *Nucleic Acids Res.* 36 D977–D981. 17947329 10.1093/nar/gkm785 PMC2238996

[B354] Yan B. Liu R. Li Y. Wang Y. Gao G. Zhang Q. (2014). QTL analysis on rice grain appearance quality, as exemplifying the typical events of transgenic or backcrossing breeding. *Breed. Sci.* 64 231–239. 25320558 10.1270/jsbbs.64.231 PMC4154612

[B355] Yang A. Dai X. Zhang W.-H. (2012). A R2R3-type MYB gene, OsMYB2, is involved in salt, cold, and dehydration tolerance in rice. *J. Exp. Bot.* 63 2541–2556. 10.1093/jxb/err431 22301384 PMC3346221

[B356] Yang C. Li D. Mao D. Liu X. Ji C. Li X. (2013). Overexpression of micro RNA 319 impacts leaf morphogenesis and leads to enhanced cold tolerance in rice (O ryza sativa L.). *Plant Cell Environ.* 36 2207–2218. 10.1111/pce.12130 23651319

[B357] Yang J. Li D. Liu H. Liu Y. Huang M. Wang H. (2020). Identification of QTLs involved in cold tolerance during the germination and bud stages of rice (Oryza sativa L.) via a high-density genetic map. *Breed. Sci.* 70 292–302. 10.1270/jsbbs.19127 32714051 PMC7372033

[B358] Yang Y. Ma H. (2009). Western blotting and ELISA techniques. *Researcher* 1 67–86.

[B359] Yano K. Yamamoto E. Aya K. Takeuchi H. Lo P.-C. Hu L. (2016). Genome-wide association study using whole-genome sequencing rapidly identifies new genes influencing agronomic traits in rice. *Nat. Genet.* 48:927. 10.1038/ng.3596 27322545

[B360] Yasmin S. Hafeez F. Y. Mirza M. S. Rasul M. Arshad H. M. Zubair M. (2017). Biocontrol of bacterial leaf blight of rice and profiling of secondary metabolites produced by rhizospheric *Pseudomonas aeruginosa* BRp3. *Front. Microbiol.* 8:1895. 10.3389/fmicb.2017.01895 29018437 PMC5622989

[B361] Ye X. Al-Babili S. Klöti A. Zhang J. Lucca P. Beyer P. (2000). Engineering the provitamin A (β-carotene) biosynthetic pathway into (carotenoid-free) rice endosperm. *Science* 287 303–305. 10.1126/science.287.5451.303 10634784

[B362] Yi X. Du Z. Su Z. (2013). PlantGSEA: a gene set enrichment analysis toolkit for plant community. *Nucleic Acids Res.* 41 W98–W103. 10.1093/nar/gkt281 23632162 PMC3692080

[B363] Yi X. Zhang Z. Ling Y. Xu W. Su Z. (2015). PNRD: a plant non-coding RNA database. *Nucleic Acids Res.* 43 D982–D989. 25398903 10.1093/nar/gku1162 PMC4383960

[B364] Yim W. C. Yu Y. Song K. Jang C. S. Lee B.-M. (2013). PLANEX: the plant co-expression database. *BMC Plant Biol.* 13:83. 10.1186/1471-2229-13-83 23688397 PMC3663717

[B365] Yonemaru J.-I. Ebana K. Yano M. (2014). HapRice, an SNP haplotype database and a web tool for rice. *Plant Cell Physiol.* 55 e9–e9. 10.1093/pcp/pct188 24334415

[B366] Yoshino K. Numajiri Y. Teramoto S. Kawachi N. Tanabata T. Tanaka T. (2019). Towards a deeper integrated multi-omics approach in the root system to develop climate-resilient rice. *Mol. Breed.* 39 1–19.

[B367] Yu H. Jiao B. Lu L. Wang P. Chen S. Liang C. (2018). NetMiner-an ensemble pipeline for building genome-wide and high-quality gene co-expression network using massive-scale RNA-seq samples. *PLoS One* 13:e0192613. 10.1371/journal.pone.0192613 29425247 PMC5806890

[B368] Yu J. Hu S. Wang J. Wong G. K.-S. Li S. Liu B. (2002). A draft sequence of the rice genome (Oryza sativa L. ssp. indica). *Science* 296 79–92. 11935017 10.1126/science.1068037

[B369] Yu X. Yang T. Qi Q. Du Y. Shi J. Liu X. (2021). Comparison of the contents of phenolic compounds including flavonoids and antioxidant activity of rice (Oryza sativa) and Chinese wild rice (Zizania latifolia). *Food Chem.* 344:128600. 10.1016/j.foodchem.2020.128600 33221101

[B370] Yuan C. Meng X. Li X. Illing N. Ingle R. A. Wang J. (2017). PceRBase: a database of plant competing endogenous RNA. *Nucleic Acids Res.* 45 D1009–D1014. 10.1093/nar/gkw916 28053167 PMC5210625

[B371] Zainal-Abidin R.-A. Abu-Bakar N. Sew Y.-S. Simoh S. Mohamed-Hussein Z.-A. (2019). Discovery of Functional SNPs via Genome-Wide Exploration of Malaysian Pigmented Rice Varieties. *Int. J. Genom.* 2019:4168045. 10.1155/2019/4168045 31687375 PMC6811786

[B372] Zainal-Abidin R.-A. Zainal Z. Mohamed-Hussein Z.-A. Abu-Bakar N. Ab Razak M. S. F. Simoh S. (2020). RNA-seq data from whole rice grains of pigmented and non-pigmented Malaysian rice varieties. *Data Brief* 30:105432. 10.1016/j.dib.2020.105432 32280737 PMC7138961

[B373] Zarei I. Luna E. Leach J. E. Mcclung A. Vilchez S. Koita O. (2018). Comparative rice bran metabolomics across diverse cultivars and functional rice gene–bran metabolite relationships. *Metabolites* 8:63. 10.3390/metabo8040063 30304872 PMC6315861

[B374] Zhang J. Zhou Z. Bai J. Tao X. Wang L. Zhang H. (2020). Disruption of MIR396e and MIR396f improves rice yield under nitrogen-deficient conditions. *Natl. Sci. Rev.* 7 102–112. 34692021 10.1093/nsr/nwz142 PMC8288854

[B375] Zhang F. Huang L. Zeng D. Cruz C. V. Li Z. Zhou Y. (2020). Comparative proteomic analysis reveals novel insights into the interaction between rice and Xanthomonas oryzae pv. oryzae. *BMC Plant Biol.* 20:1–14. 10.1186/s12870-020-02769-7 33317452 PMC7734852

[B376] Zhang H. Ma H. Xie X. Ji J. Dong Y. Du Y. (2014). Comparative proteomic analyses reveal that the regulators of G-protein signaling proteins regulate amino acid metabolism of the rice blast fungus Magnaporthe oryzae. *Proteomics* 14 2508–2522. 10.1002/pmic.201400173 25236475

[B377] Zhang J. Zhang H. Srivastava A. K. Pan Y. Bai J. Fang J. (2018). Knockdown of rice microRNA166 confers drought resistance by causing leaf rolling and altering stem xylem development. *Plant Physiol.* 176 2082–2094. 10.1104/pp.17.01432 29367235 PMC5841683

[B378] Zhang X. Yin F. Xiao S. Jiang C. Yu T. Chen L. (2019). Proteomic analysis of the rice (Oryza officinalis) provides clues on molecular tagging of proteins for brown planthopper resistance. *BMC Plant Biol.* 19:1–11. 10.1186/s12870-018-1622-9 30658570 PMC6339371

[B379] Zhang Y. Zang Q. Xu B. Zheng W. Ban R. Zhang H. (2016). IsomiR Bank: a research resource for tracking IsomiRs. *Bioinformatics* 32 2069–2071. 10.1093/bioinformatics/btw070 27153728

[B380] Zhang Y.-C. Yu Y. Wang C.-Y. Li Z.-Y. Liu Q. Xu J. (2013). Overexpression of microRNA OsmiR397 improves rice yield by increasing grain size and promoting panicle branching. *Nat. Biotechnol.* 31 848–852. 10.1038/nbt.2646 23873084

[B381] Zhao B. Ge L. Liang R. Li W. Ruan K. Lin H. (2009). Members of miR-169 family are induced by high salinity and transiently inhibit the NF-YA transcription factor. *BMC Mol. Biol.* 10:1–10. 10.1186/1471-2199-10-29 19351418 PMC2670843

[B382] Zhao B. Liang R. Ge L. Li W. Xiao H. Lin H. (2007). Identification of drought-induced microRNAs in rice. *Biochem. Biophys. Res. Commun.* 354 585–590. 10.1016/j.bbrc.2007.01.022 17254555

[B383] Zhao H. Yao W. Ouyang Y. Yang W. Wang G. Lian X. (2015). RiceVarMap: a comprehensive database of rice genomic variations. *Nucleic Acids Res.* 43 D1018–D1022. 10.1093/nar/gku894 25274737 PMC4384008

[B384] Zhao X.-Q. Wang W.-S. Zhang F. Zhang T. Zhao W. Fu B.-Y. (2013). Temporal profiling of primary metabolites under chilling stress and its association with seedling chilling tolerance of rice (Oryza sativa L.). *Rice* 6 1–13. 10.1186/1939-8433-6-23 24280004 PMC4883686

[B385] Zhao Y. F. Peng T. Sun H. Z. Teotia S. Wen H. L. Du Y. X. (2019). miR1432-Os ACOT (Acyl-CoA thioesterase) module determines grain yield via enhancing grain filling rate in rice. *Plant Biotechnol. J.* 17 712–723. 10.1111/pbi.13009 30183128 PMC6419572

[B386] Zhao Y. Wen H. Teotia S. Du Y. Zhang J. Li J. (2017). Suppression of microRNA159 impacts multiple agronomic traits in rice (Oryza sativa L.). *BMC Plant Biol.* 17:1–13. 10.1186/s12870-017-1171-7 29162059 PMC5699021

[B387] Zheng H. Zhao H. Liu H. Wang J. Zou D. (2015). QTL analysis of Na+ and K+ concentrations in shoots and roots under NaCl stress based on linkage and association analysis in japonica rice. *Euphytica* 201 109–121.

[B388] Zheng W. Ma L. Zhao J. Li Z. Sun F. Lu X. (2013). Comparative transcriptome analysis of two rice varieties in response to rice stripe virus and small brown planthoppers during early interaction. *PLoS One* 8:e82126. 10.1371/journal.pone.0082126 24358146 PMC3864904

[B389] Zhou J. Wang F. Deng P. Jing W. Zhang W. (2013). Characterization and mapping of a salt-sensitive mutant in rice (Oryza sativa L.). *J. Integrat. Plant Biol.* 55 504–513. 10.1111/jipb.12048 23480486

[B390] Zhou L. Liu Y. Liu Z. Kong D. Duan M. Luo L. (2010). Genome-wide identification and analysis of drought-responsive microRNAs in Oryza sativa. *J. Exp. Bot.* 61 4157–4168. 10.1093/jxb/erq237 20729483

[B391] Zhou Y. Tang J. Walker M. G. Zhang X. Wang J. Hu S. (2003). Gene identification and expression analysis of 86,136 Expressed Sequence Tags (EST) from the rice genome. *Genomics Proteom. Bioinformat.* 1 26–42. 10.1016/s1672-0229(03)01005-2 PMC5172415 15626331

[B392] Zhu Q.-H. Upadhyaya N. M. Gubler F. Helliwell C. A. (2009). Over-expression of miR172 causes loss of spikelet determinacy and floral organ abnormalities in rice (Oryza sativa). *BMC Plant Biol.* 9:149. 10.1186/1471-2229-9-149 20017947 PMC2803185

